# Facile synthesis of *trans* racemic 1,4-diaryl substituted, proline esters *via* Ugi multicomponent reaction

**DOI:** 10.1039/d6ra07669f

**Published:** 2026-10-06

**Authors:** Siva Krishna Polineni, P. Sathya Shanker, Tony Kurissery Anthappan, Vipin A. Nair

**Affiliations:** a Syngene International Limited Biocon Park, Bommasandra IV Phase, Jigani Link Road Bangalore 560099 Karnataka India; b Department of Chemistry, REVA University Kattigenahalli, Yelahanka Bangalore 560064 Karnataka India; c School of Biotechnology, Amrita Vishwa Vidyapeetham, Amritapuri Campus Clappana Kollam 690525 Kerala India vn74nr@gmail.com

## Abstract

Diaryl-substituted pyrrolidine derivatives are prevalent in numerous biologically active compounds and represent important scaffolds in medicinal chemistry. However, their synthesis remains challenging due to limitations associated with direct functionalization of the pyrrolidine ring. This work describes a facile synthesis of 1,4-diaryl-substituted pyrrolidine derivatives *via* Ugi multicomponent coupling reaction. The strategy enables the convergent assembly of highly functionalized pyrrolidine frameworks from readily available aromatic building blocks, thereby providing an effective alternative to conventional functionalization approaches. The operational simplicity and modularity of this method offer a practical and versatile route to pyrrolidine scaffolds of relevance to both synthetic and medicinal chemistry.

## Abstract 2

An efficient and concise Ugi multicomponent strategy for the synthesis of functionalized pyrrolidine derivatives using hemiacetals as aldehyde surrogates.

## Graphical abstract

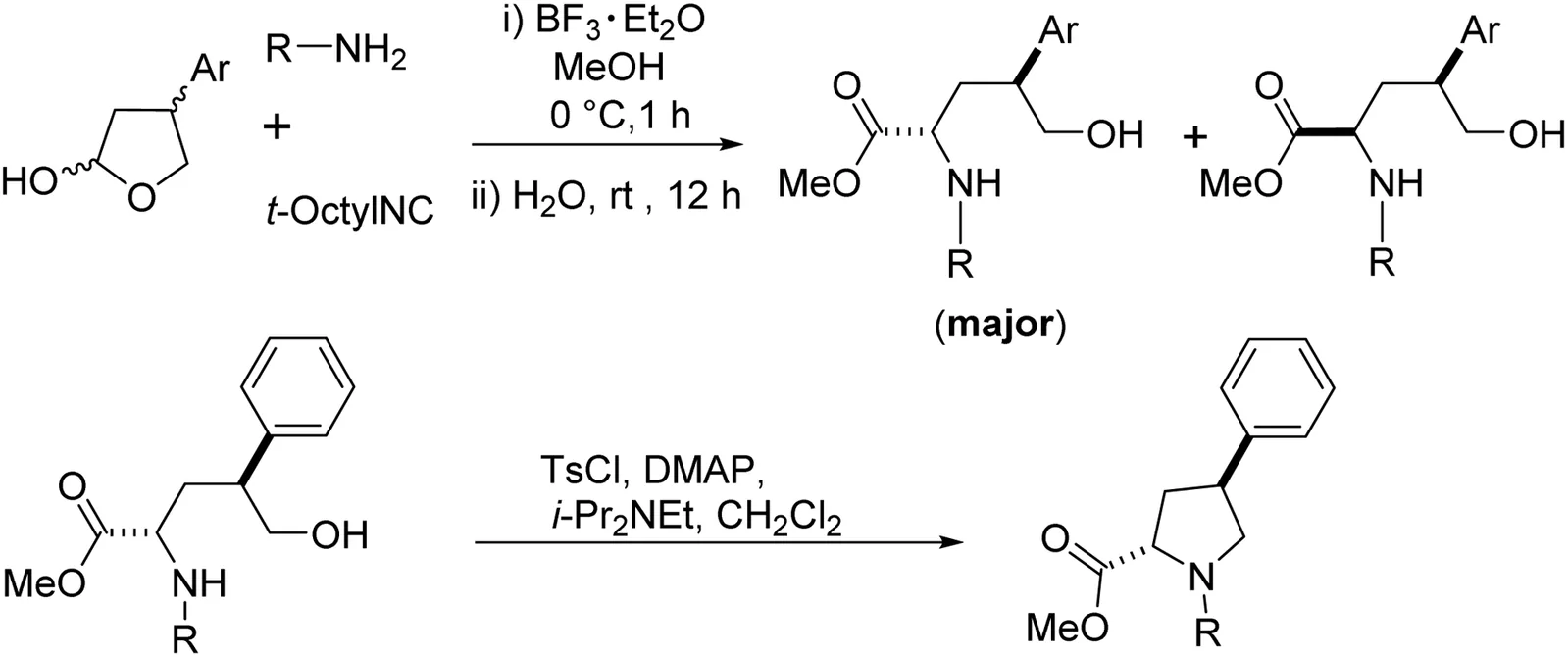

## Introduction

Pyrrolidine motifs are important due to their presence in proline, a cyclic amino acid that enables peptide functionalization and precise conformational control. Their structural features and ease of modification make proline derivatives valuable in molecular design.^1^ Accordingly, they have been widely applied in biological and chemical processes such as ion-gating and asymmetric catalysis.^2–4^ Considerable effort has been devoted to the development of selective functionalization strategies for pyrrolidines. Ru- and Pd-catalyzed transformations have enabled efficient C(2)- and C(3)-functionalization, providing access to enantiomerically enriched and biologically relevant derivatives.^5–9^ Complementary approaches, such as silver-catalyzed cyclization of propargylglycine derivatives, allow rapid construction of 2,5-disubstituted pyrrolidines.^10^

Functionalization at the C(4) position is equally significant, as this site is frequently substituted in bioactive molecules. Proline derivatives bearing C(4) heteroatom or alkyl substituents are common motifs in natural products and pharmaceuticals, including lincomycin analogues and the ACE inhibitor fosinopril.^11^ Synthetic access to 4-methylprolines has been achieved through a range of transformations, including radical cyclization, asymmetric hydrogenation, and diastereoselective alkylation and ring-forming processes.^12–14^ (Fig. 1). Despite these advances, many established methods rely on expensive transition-metal catalysts or hazardous reagents, which can limit their practicality, particularly for large-scale synthesis.^15^

**Fig. 1:**
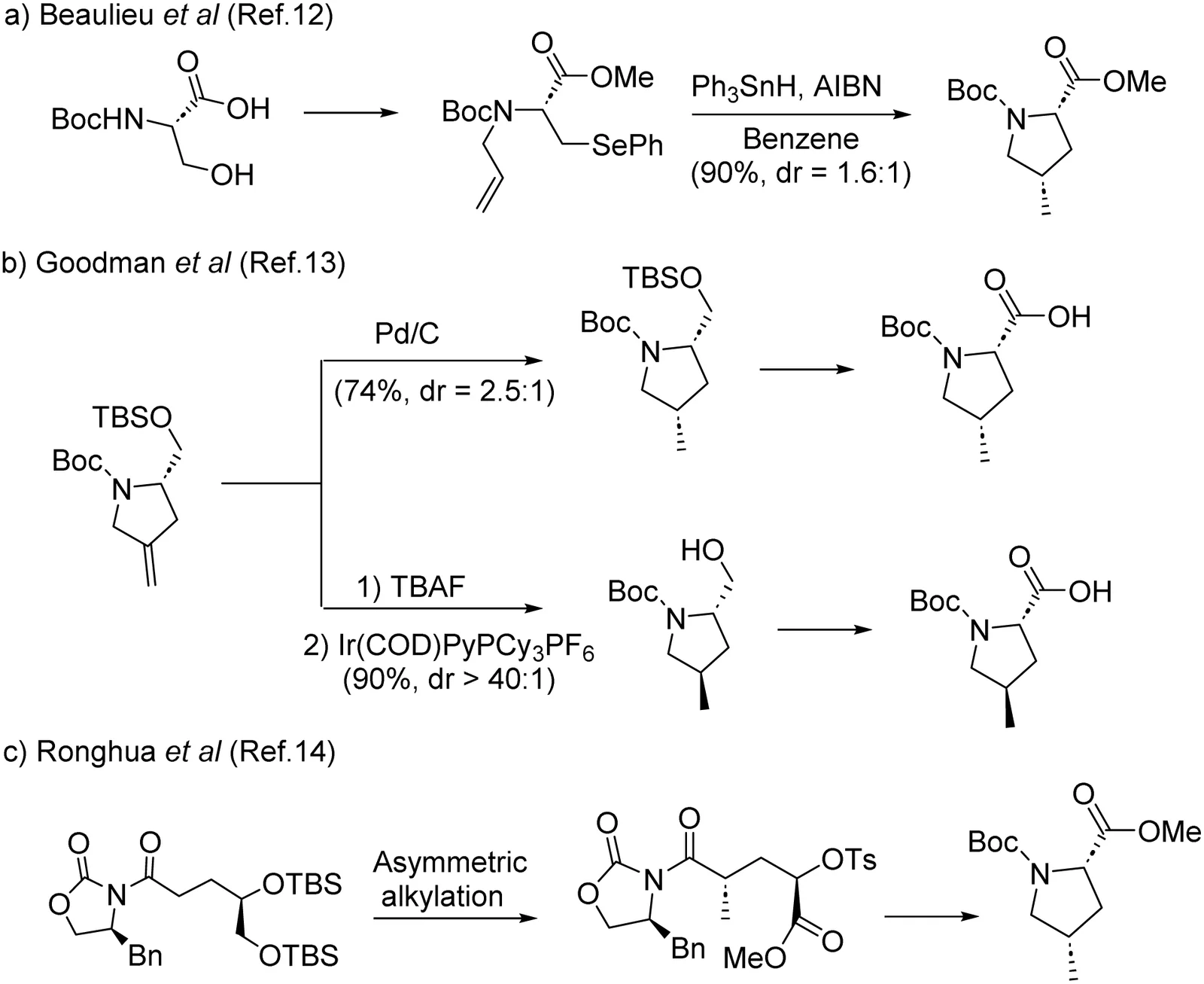
Functionalization approaches to 4-methyl pyrrolidines (a) radical cyclization, (b) asymmetric hydrogenation, (c) diastereoselective alkylation.

Among substituted pyrrolidines, 1,4-disubstituted proline derivatives, especially 4-aryl *N*-phenyl pyrrolidine esters represent an important class of bioactive molecules. These structures combine the conformational rigidity of the pyrrolidine ring^16^ with precise stereochemical control,^17–21^ while aryl substitution at the C(4) position enhances π–π interactions and improves binding affinity toward biological targets.^22^ The ester functionality contributes to lipophilicity and membrane permeability, whereas *N*-aryl substitution further enhances molecular stability and biological performance.^23^ Despite their significance, efficient synthetic routes to these frameworks remain limited. Convergent strategies enabling the simultaneous installation of *N*-aryl and C(4)-aryl substituents remain underdeveloped, presenting a notable synthetic challenge.^11^

The synthesis of 1,4-disubstituted proline derivatives therefore continues to attract considerable attention. The development of efficient, selective, and broadly applicable methodologies is essential for expanding chemical space and facilitating the discovery of new biologically active molecules. In this context, multicomponent reactions offer a powerful platform for rapid and convergent molecular assembly. Among these, the Ugi reaction has emerged as a versatile and efficient strategy for the construction of structurally diverse and highly functionalized molecules from simple starting materials. Recent advances have further highlighted the utility of this reaction in natural product synthesis, demonstrating its ability to rapidly generate molecular complexity and provide access to biologically relevant scaffolds.^24^ Herein, we report the synthesis of 1,4-diaryl-substituted pyrrolidine derivatives *via* the Ugi multicomponent reaction, enabling simultaneous incorporation of structural diversity at both the nitrogen and C(4) positions. This approach provides access to structurally diverse pyrrolidine scaffolds under operationally simple conditions (Fig. 2).

**Fig. 2:**
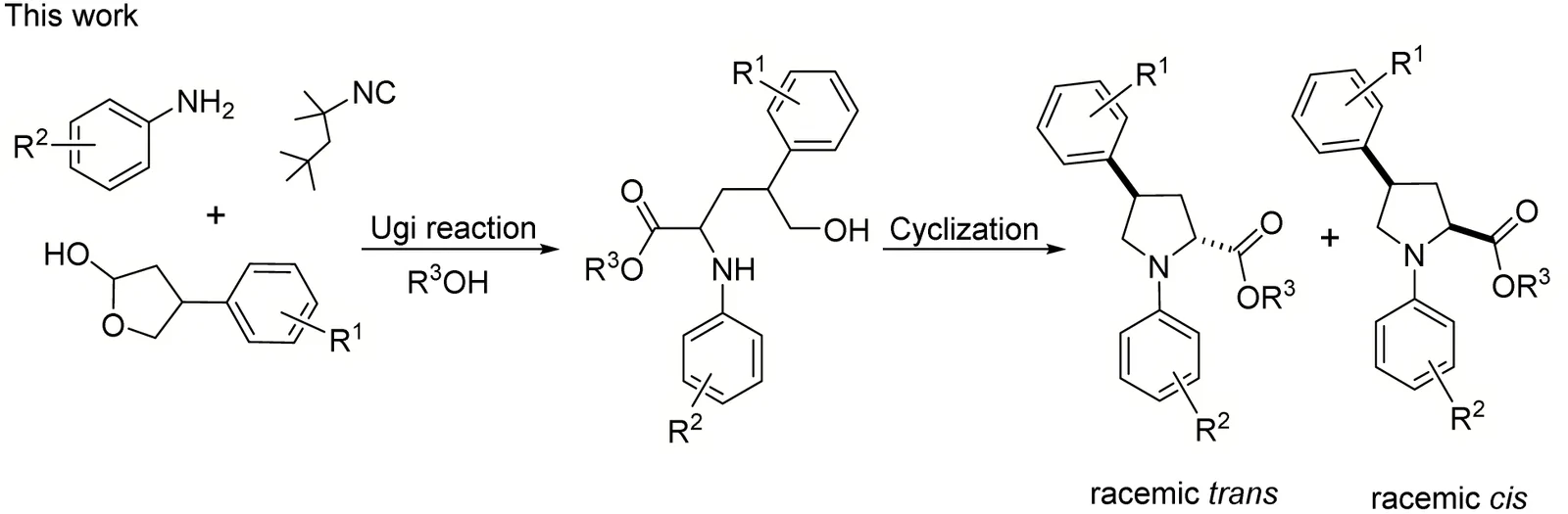
Synthesis of 1,4-disubstituted proline derivatives.

## Results and discussion

For constructing substituted pyrrolidines, we devised a strategic approach which involves the versatility of the Ugi multicomponent reaction, a powerful synthetic tool known for assembling complex molecules in a one-pot process. Accordingly, hemiacetals, aryl amines, and isonitriles were chosen as the starting materials, taking advantage of their reactive potential to form the desired pyrrolidine scaffolds efficiently. Hemiacetals, despite being considered unstable was utilized for their equilibrium behaviour, wherein they can reversibly interconvert with aldehydes under suitable conditions. The aldehydes then participate in the formation of an imine, which is a necessary intermediate for the subsequent Ugi multicomponent reaction. To realize this concept, we first synthesized aryl-substituted hemiacetals by an efficient redox-relay Heck reaction.^25^ This approach enabled rapid assembly of cyclic hemiacetals 3(a–d) from aryl iodides 1(a–d) and the alkene 2 (Scheme 1). This methodology not only provided a straightforward route to our key reaction intermediates but also ensured that the resulting hemiacetals possessed the appropriate structural features and stability required for further transformations. We synthesized four different hemiacetals, each a mixture of *cis* and *trans* isomers. Attempts to prepare 4-nitrophenyl or pyridine substituted hemiacetals were unsuccessful.

**Scheme 1:**
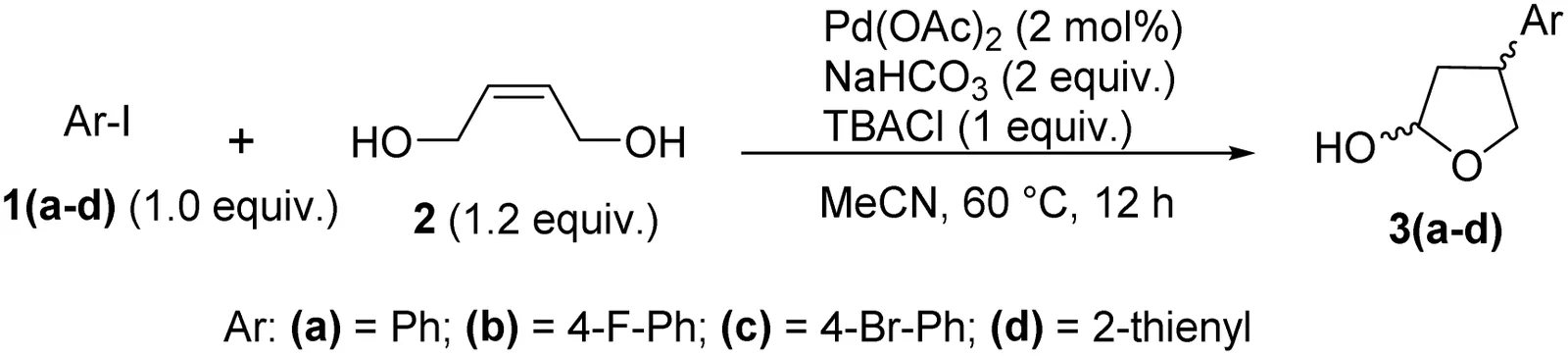
Synthesis of aryl substituted hemiacetals.

Our investigation commenced with the application of the Ugi multicomponent reaction utilizing the substrate 3(a) (Scheme 2). To optimize the reaction conditions, we conducted studies to identify the reaction parameters that govern efficiency and chemoselectivity of the Ugi transformation, especially the choice of catalyst and its role in promoting ester formation over the conventional amide pathway. The outcomes of these investigations are summarized in Table 1. Following the previously reported procedure,^26^ the synthesis afforded a glycine derivative 6(a)(i), which was obtained in low yield (entry 1, Table 1). To improve the reaction efficiency, screening of various catalysts was carried out. Initial attempts involved the use of indium trichloride (3.0 equiv.), and *p*-toluenesulfonic acid (3.0 equiv.). These catalysts are known for their ability to facilitate multicomponent reactions;^27,28^ unfortunately, neither of them led to a notable enhancement in the formation of the desired ester product 6(a)(i). The yields and selectivity remained poor, affirming that a wider search is necessary. Subsequently, investigation into metal triflates was conducted, evaluating scandium triflate (Sc(OTf)_3_), magnesium triflate (Mg(OTf)_2_), copper triflate (Cu(OTf)_2_), and indium triflate (In(OTf)_2_). Despite their efficacy in other multicomponent reactions, these triflates also failed to produce better results for the study herein. The reaction predominantly yielded the conventional Ugi product, the amide derivative 7(a)(i) rather than the desired ester derivative 6(a)(i). This indicated that the standard conditions and common catalysts were inefficient for steering the reaction towards the ester formation, highlighting the challenges associated with this reaction.

**Scheme 2:**
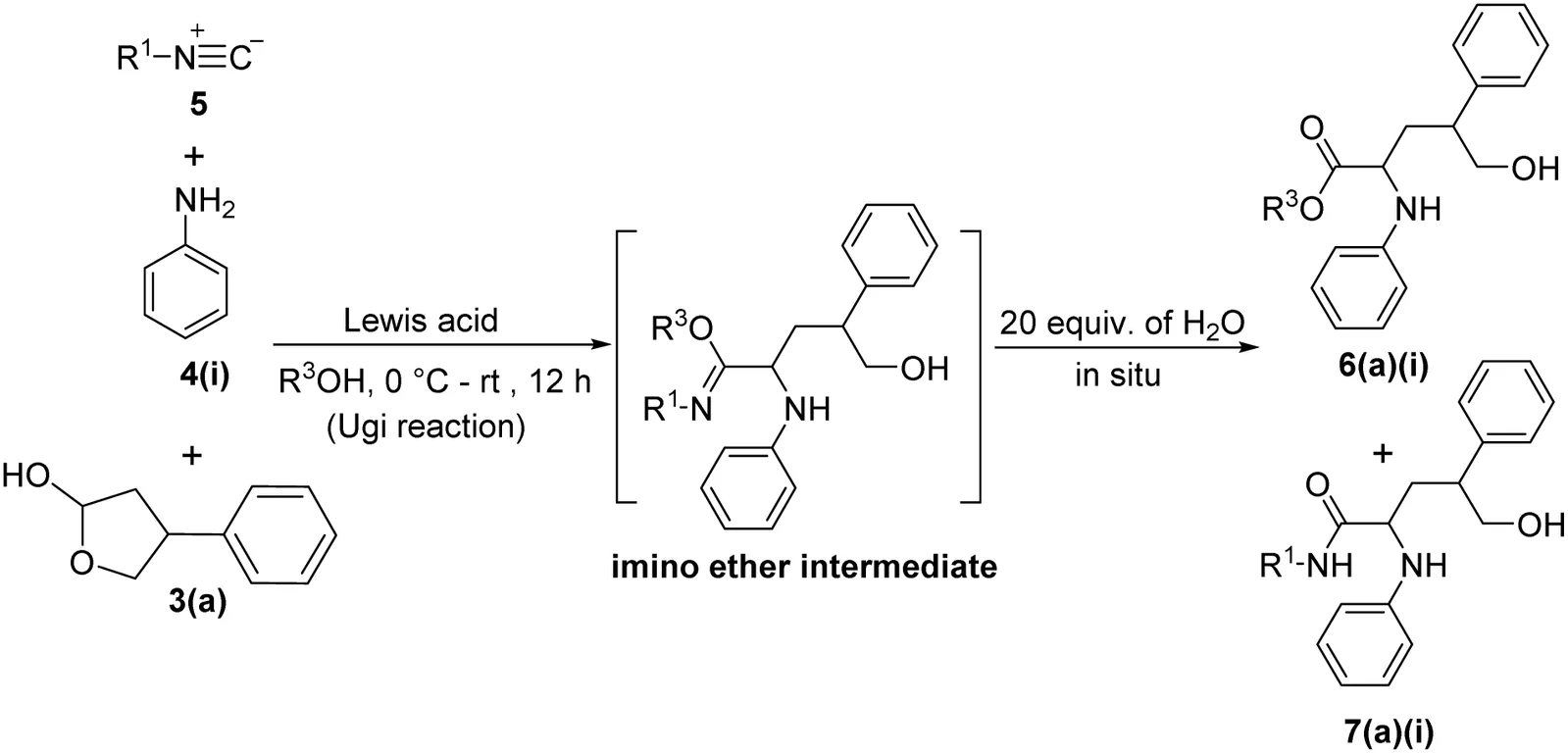
Standardization of Ugi multicomponent reaction using hemiacetal.

**Table 1:** Optimization of the conditions for Ugi multicomponent reaction^a^

No.	Lewis acid	Isocyanide, *R*^1^	Solvent^b^	Yield,^d^6(a)(i)	Yield,^d^7(a)(i)
1	BF_3_·Et_2_O	Bn	MeOH	21	—
2	InCl_3_	Bn	MeOH	—	11
3	*p*-TsOH	Bn	MeOH	5	1
4	Sc(OTf)_3_	Bn	MeOH	—	8
5	Mg(OTf)_2_	Bn	MeOH	—	10
6	Cu(OTf)_2_	Bn	MeOH	2	6
7	In(OTf)_2_	Bn	MeOH	5	11
8	BF_3_·Et_2_O	*t*-Octyl	MeOH	67	—
9	BF_3_·Et_2_O	*t*-Butyl	MeOH	11	—
10	BF_3_·Et_2_O	*t*-Octyl	EtOH	32	10
11	BF_3_·Et_2_O	*t*-Octyl	*t*-BuOH	24	18
12	BF_3_·Et_2_O	*t*-Octyl	MeOH^c^	44	—
13	BF_3_·Et_2_O	*t*-Butyl	EtOH	31	7

a 3(a), 1.0 equiv.; 4(i), 1.0 equiv.; 5, 1.0 equiv.; Lewis acid, 3.0 equiv.; solvent, 10 mL.

b Temp., RT.

c Temp., 60 °C.

d Diastereomeric mixture.

Based on these findings, our focus shifted back to optimizing the conditions using boron trifluoride diethyl etherate (BF_3_·OEt_2_) (3.0 equiv.) as the catalyst. We now varied the isocyanide component, which plays a critical role in the Ugi reaction. Alternative isocyanides such as 2-isocyano-2,4,4-trimethylpentane (*t*-OctylNC) and 2-isocyanopropane (*t*-ButylNC) were selected for their differing steric and electronic properties, aiming to enhance the reaction's efficiency and selectivity. The results indicated that the most favourable conversion to the desired product was achieved when BF_3_·OEt_2_ was used along with the *t*-OctylNC in methanol, (entry 8, Table 1). This combination provided the best catalyst activity and solvation effects necessary for ester formation. Additional attempts to improve the reaction by increasing the temperature did not yield significant outcomes (entry 12, Table 1). The observation underscored the limited effect of thermal variation under the conditions employed. Thus, the synthesis of the glycine ester, a key intermediate in the overall reaction pathway was achieved through the multicomponent Ugi reaction. Though this method efficiently combines multiple reactants in a single step to generate glycine esters, it also produced a mixture of stereoisomeric forms of the products (Scheme 3). To explore the scope of the methodology, a diverse array of substituted anilines was employed in the Ugi reaction, allowing an evaluation of substituent effects (Fig. 3). In all the cases, the reaction facilitated the formation of *cis* and the *trans* isomers in an approximate ratio of 1 : 8, respectively.

**Scheme 3:**
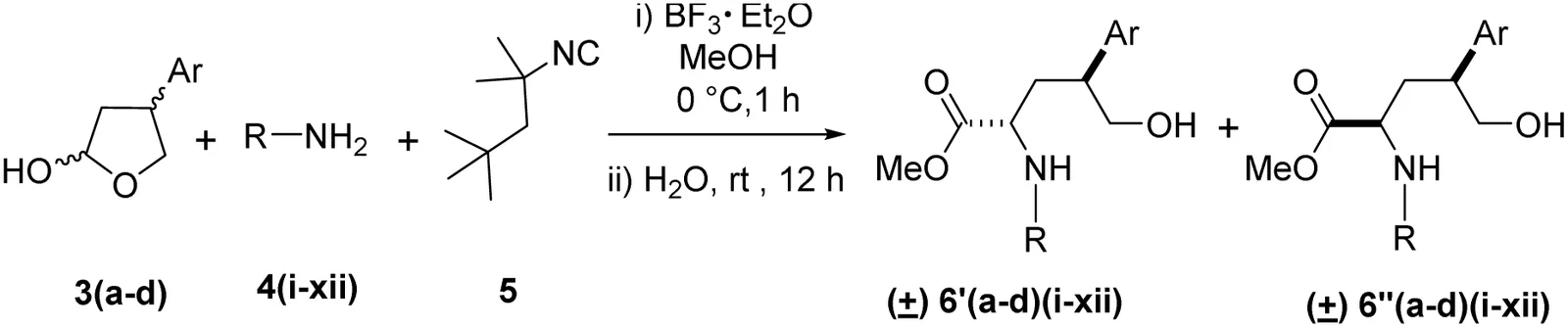
Ugi reaction of hemiacetals.

**Fig. 3:**
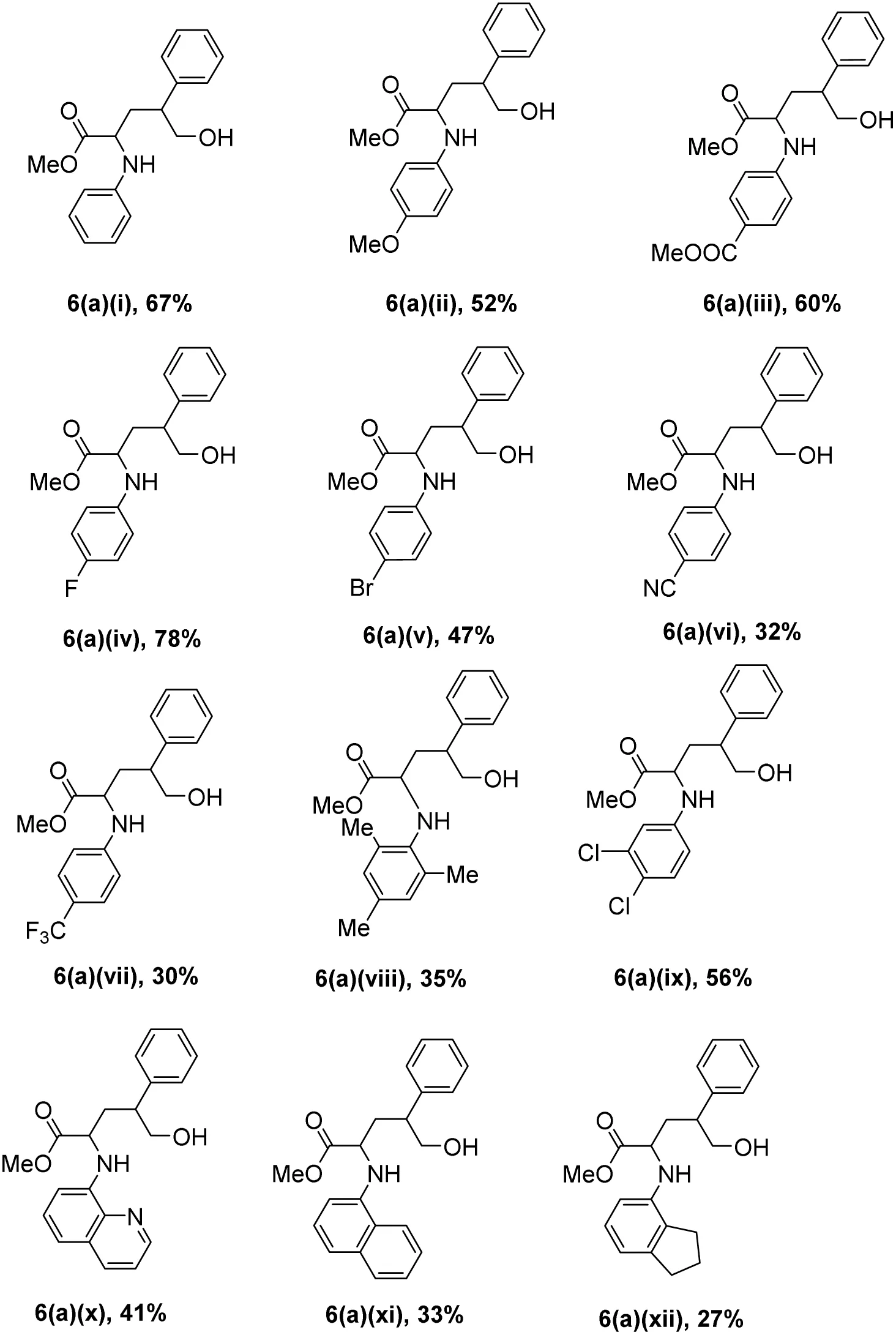
Synthesized Ugi multicomponent reaction products * yield of the product calculated from the mixture of diastereomers.

In terms of substrate compatibility, amine derivatives demonstrated tolerance under the reaction conditions. The electronic and steric effects of various substituents on the aromatic ring did not substantially hinder the formation of the Ugi reaction products. Interestingly, the reaction involving 4-amino methyl benzoate was found to proceed differently; the reaction primarily afforded the conventional Ugi product. This product was hydrolysed by heating with methanol, effectively converting it into the corresponding glycine ester. This deviation highlights the versatility of the reaction condition in enabling post-reaction modification and provides a strategic route for accessing amino acid derivatives. Attempts to isolate glycine esters derived from aliphatic amines, proved significantly more challenging. Aliphatic amines including methylamine, cyclopropylamine, cyclopentylamine, and cyclohexylamine were examined under the optimized reaction conditions. Nevertheless, these substrates exhibited poor compatibility because of their greater polarity. The generation of complex reaction mixtures hindered efficient reaction control and product purification. Consequently, the corresponding glycine esters could not be isolated in analytically pure form, highlighting the superior suitability of aromatic amines for this transformation.

Following the formation of the Ugi reaction product 6(a)(i), the major *trans* diastereomer 6′(a)(i) was isolated in racemic form from the reaction mixture and subjected to cyclization to obtain substituted pyrrolidine (Scheme 4). The intramolecular cyclization was achieved by using tosyl chloride, in the presence of *N*,*N*-dimethyl aminopyridine and diisopropyl ethyl amine to generate the racemic *trans* 1,4-disubstituted pyrrolidine derivative 8′(a)(i). This cyclization occurred with high efficiency, thereby demonstrating the robustness of the reaction sequence. To unambiguously confirm the stereochemical configuration of substituted pyrrolidine, Nuclear Overhauser Effect Spectroscopy (NOESY) was employed on the *trans* and *cis* isomers, 8'a(i) and 8″a(i) respectively (Fig. 4). This allowed for the determination of the spatial relationships between the substituents at positions 2 and 4 on the pyrrolidine ring, thereby providing essential stereochemical information. The NOESY analysis also validated the stereochemical preferences underlying the cyclization step.

**Scheme 4:**
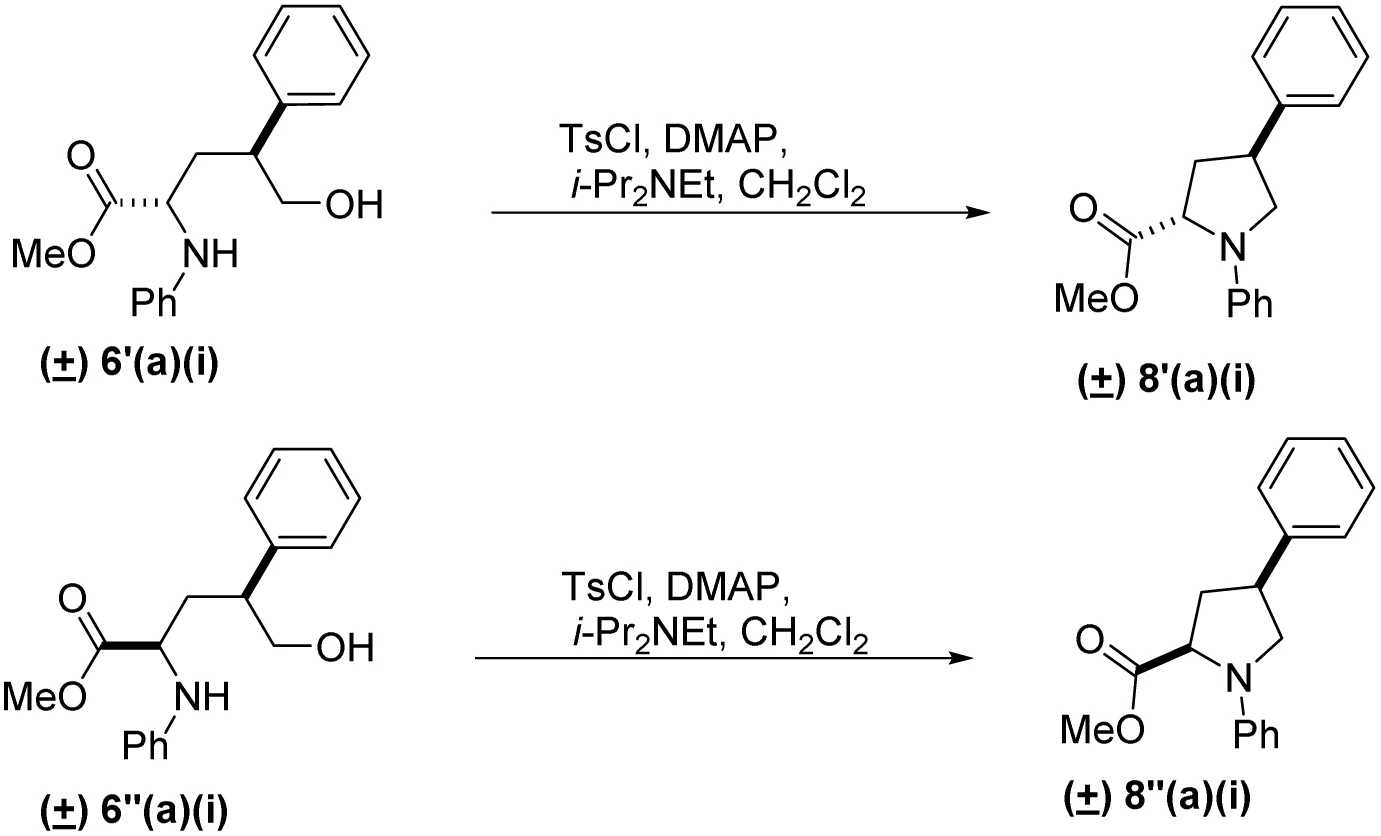
Intramolecular cyclization of Ugi reaction products.

**Fig. 4:**
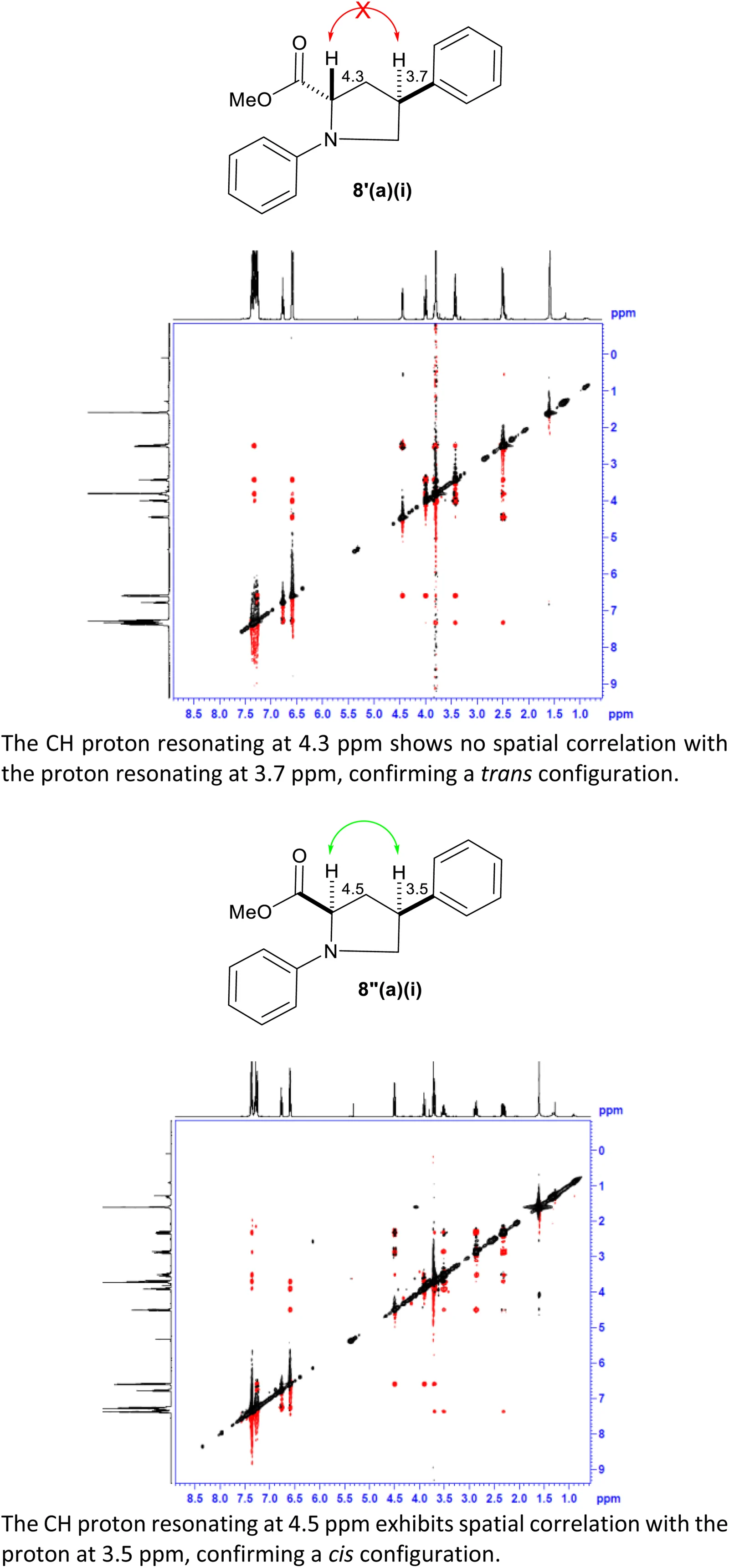
NOESY spectra to determine the spatial correlation in *trans* and *cis* isomers, 8′(a)(i) and 8″(a)(i)respectively.

The generation of both the isomers provides scope for stereoselective synthesis of desired pyrrolidine derivatives. However, the *trans* isomer being the major product from the Ugi reactions was isolated in all the cases and cyclized to obtain the racemic *trans* isomers of substituted pyrrolidines, 8′(a)(i)–8′(a)(xiv) (Fig. 5). Structural confirmation of these synthesized pyrrolidine derivatives was achieved through ^1^H & ^13^C NMR spectra, and high-resolution mass spectrometry (see SI). The reaction afforded a range of functionalized pyrrolidines with moderate to high yields which demonstrates the broad substrate scope. The general applicability of the methodology was further demonstrated through a diverse array of substrates, including various hemiacetals, and amines (Fig. 6). This also underscores its potential utility in synthesizing structurally diverse pyrrolidine frameworks. Unlike the conventional Ugi multicomponent reaction, where aldehydes typically lead to high yielding product formation, the use of hemiacetals as aldehyde surrogates diminished the overall yield of the Ugi products. This notable reduction in efficiency can be attributed to the stereochemical features of hemiacetals, which have a chiral centre at the carbon atom bearing the hydroxyl group. This caused an uneven distribution of geometric isomers, since hemiacetals exist in equilibrium between different stereoisomers.

**Fig. 5:**
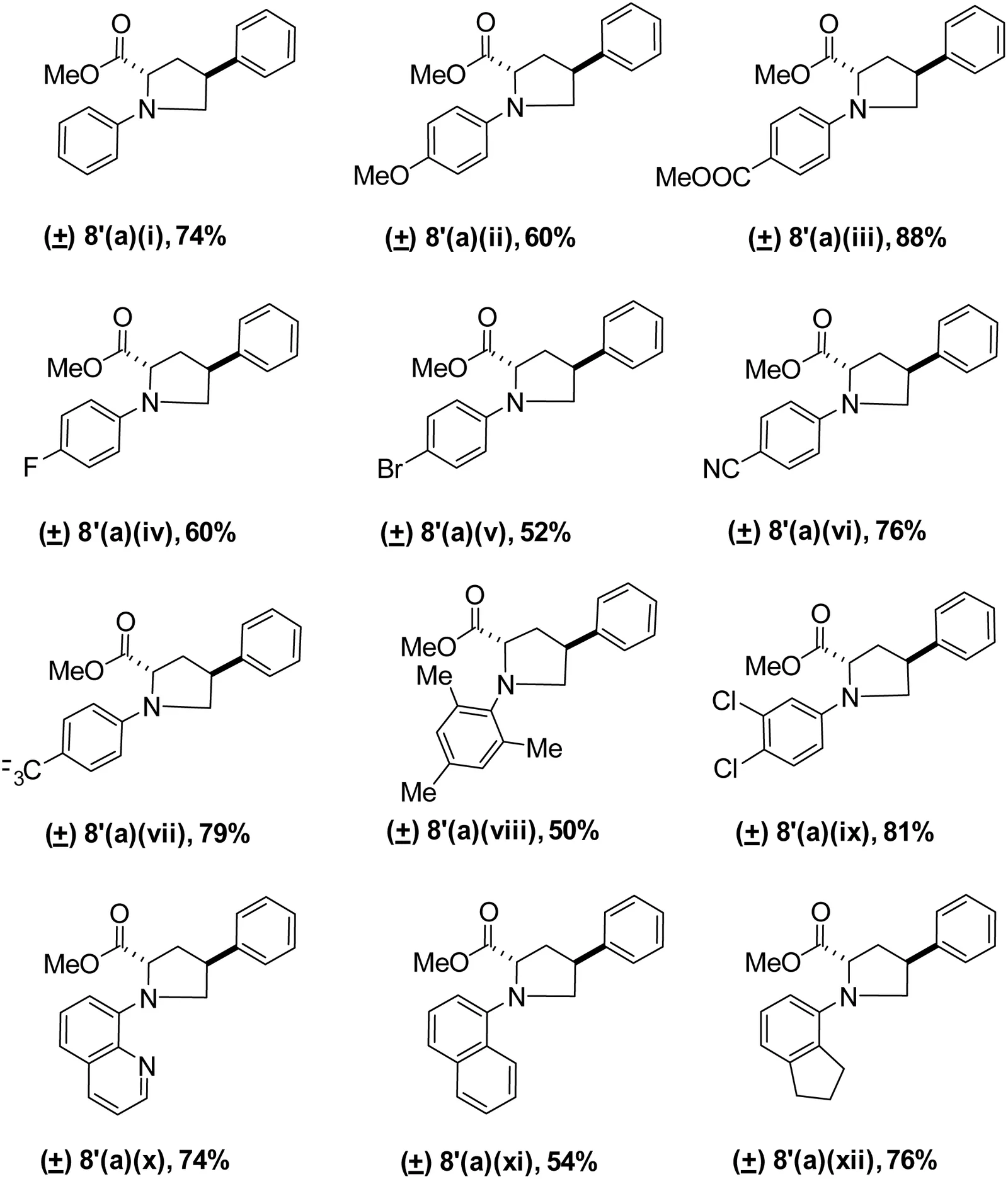
Synthesized pyrrolidine derivatives from various amines.

**Fig. 6:**
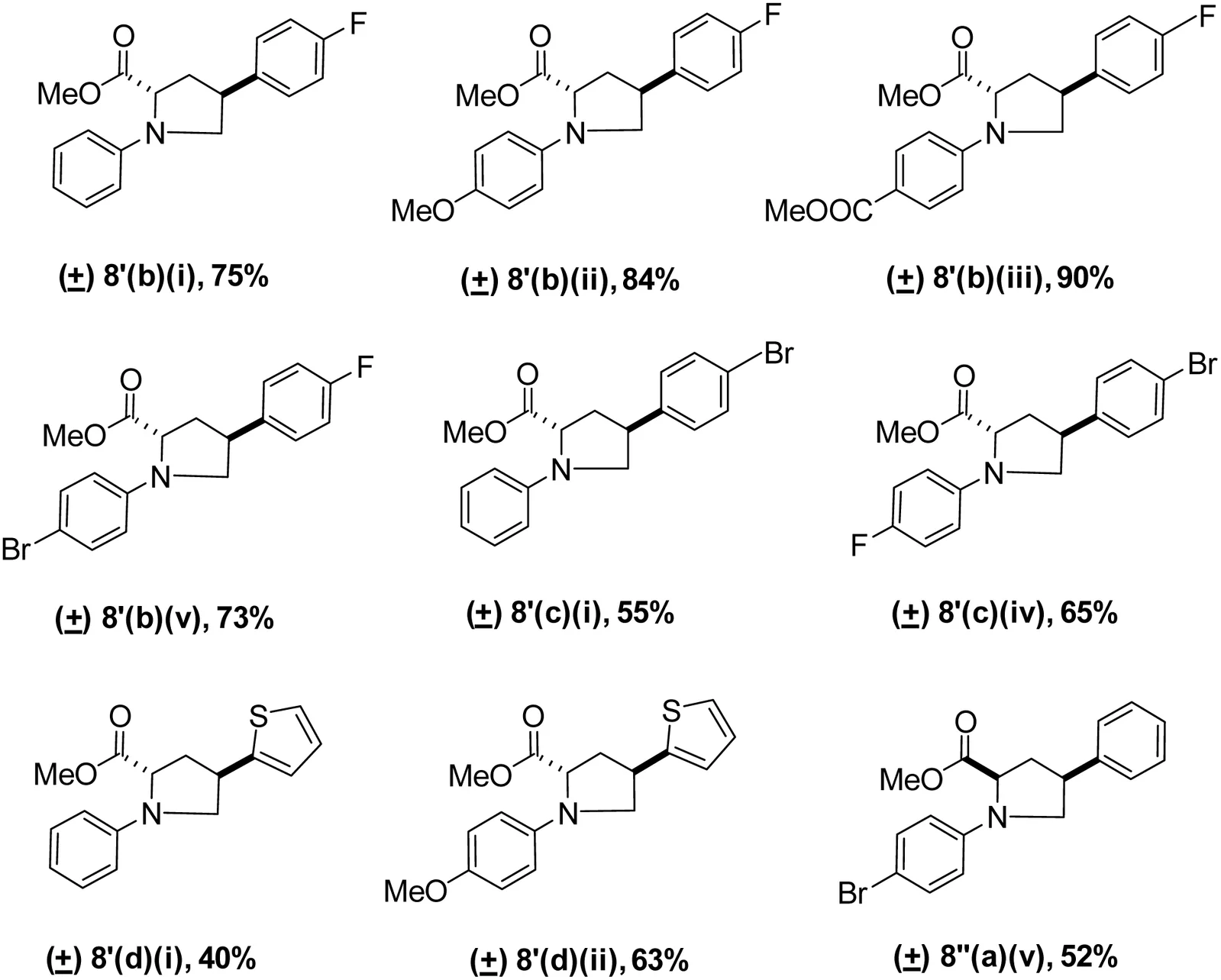
Synthesized pyrrolidine derivatives from various amines and different hemiacetals.

A plausible mechanism for the reaction is provided in Scheme 5. BF_3_·OEt_2_ activates the hemiacetal, 3(a) toward nucleophilic attack by coordinating with the oxygen atom, making the carbon more electrophilic. The amine, 4(i) attacks the activated hemiacetal, eliminates a molecule of water, and yields the iminium intermediate, A. The *in situ* generated iminium intermediate is susceptible to reaction with the isocyanide, 5. The activated imine undergoes nucleophilic attack by the isocyanide to form a stable nitrilium intermediate, B. Thereafter methanol attacks the electrophilic centre, and forms the imino ether intermediate, C, which upon hydrolysis with excess water yields the corresponding ester, 6(a)(i). Though the possibility of intramolecular cyclization of the nitrilium intermediate B in Scheme 5 is mechanistically relevant, no cyclized product was detected.

**Scheme 5:**
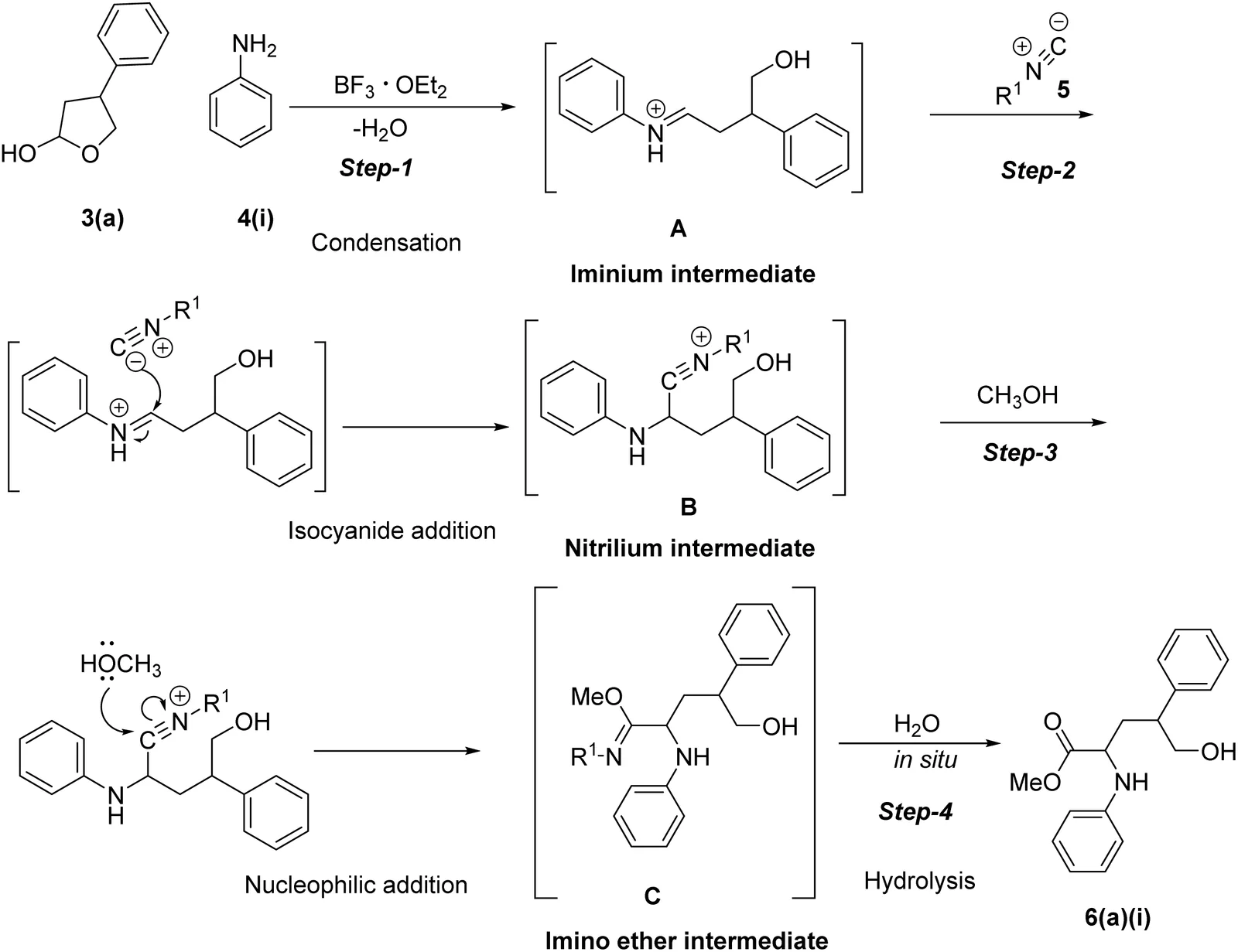
Plausible reaction mechanism.

We further extended our studies by performing derivatization reactions on selected compounds. The subsequent transformation involved functional group modification of (±) 8′(a)(v), by a cross-coupling reaction, which further embellished the molecular structures. The derivatizations highlight the methodological flexibility to serve as versatile intermediates for further synthetic elaboration. A synthetic protocol that involves the Suzuki–Miyaura cross-coupling of (±) 8′(a)(v) with 4-fluorophenylboronic acid was tried. The reaction was catalysed by 5 mol% of Pd(dppf)Cl_2_ in the presence of potassium phosphate as base, in dioxane-water solvent system (Scheme 6). The reaction was conducted at 80 °C for 3 hours.

**Scheme 6:**
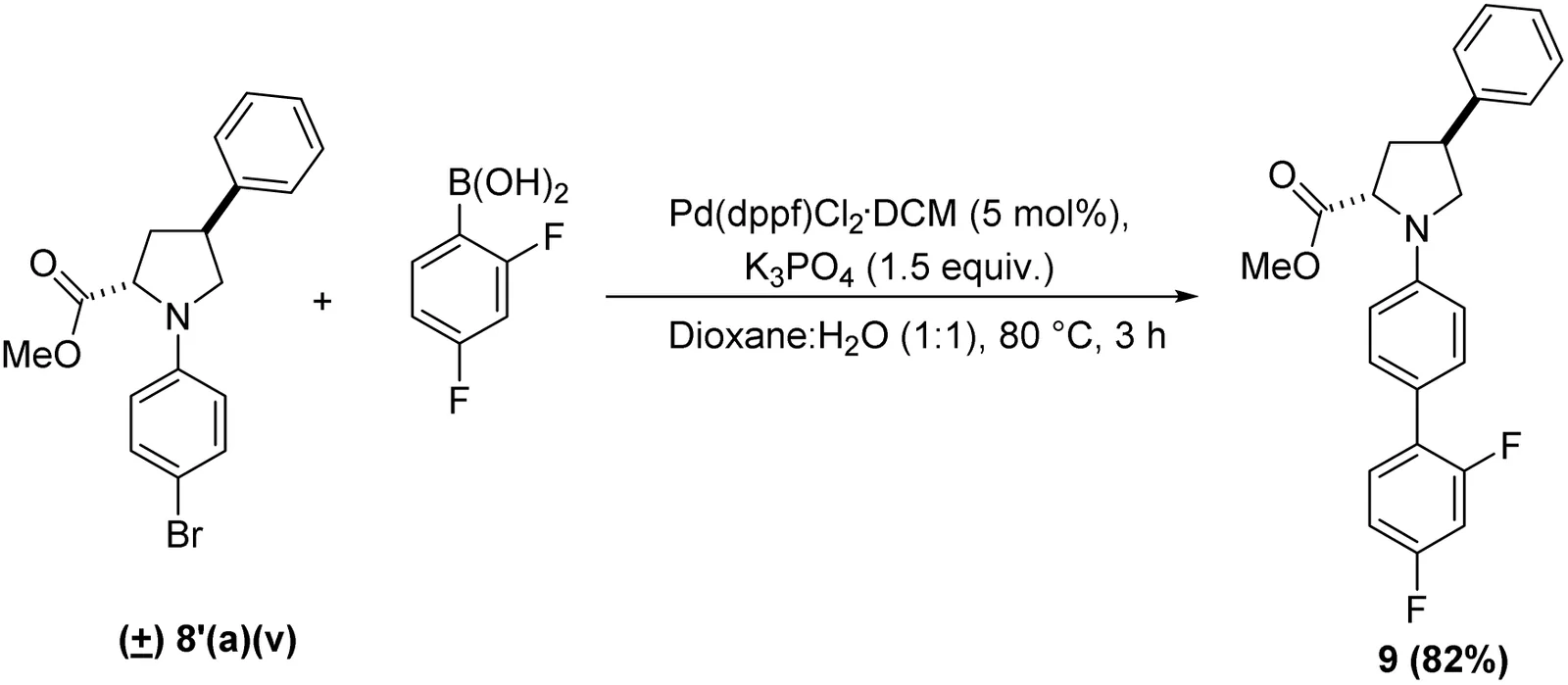
Functionalization of pyrrolidine derivative by Suzuki–Miyaura cross-coupling.

The process facilitated an efficient formation of the biaryl product 9 with isolated yield of 82%. This methodology offers a mild and selective approach to construct complex biaryl frameworks on functionalized pyrrolidines, for pharmaceutical and material applications.

In another reaction the hydrolysis of (±) 8′(a)(iv) was facilitated using lithium hydroxide in THF-water mixture (1 : 1), to afford the corresponding carboxylic acid 10 (Scheme 7). Subsequently, the acid was coupled with benzylamine using HATU as the coupling reagent and DIPEA as the base in DMF. This peptide bond formation proceeds smoothly to deliver the benzylamide derived pyrrolidine 11 in 71% yield. The sequence demonstrates an efficient two-step transformation involving ester hydrolysis followed by amide bond formation under mild condition for the synthesis of potential bioactive molecules.

**Scheme 7:**
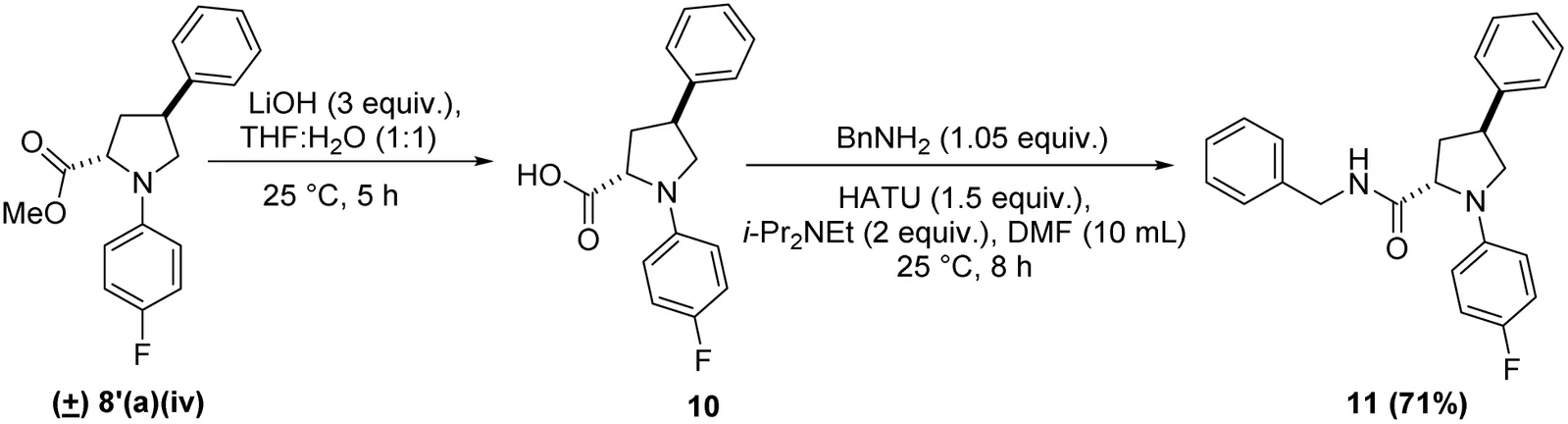
Functionalization of pyrrolidine derivative by acid–amine coupling.

## Experimental

All non-aqueous reactions were run under nitrogen atmosphere with dried glass wares using standard techniques for handling air-sensitive compounds. Chemicals were purchased from commercial sources in analytical grade and were used as supplied. All other chemicals were either commercially available or prepared according to the cited references. The reactions were carried out under nitrogen/argon atmosphere using oven-dried reaction vials. Dry solvents were prepared by distilling over sodium metal with benzophenone, calcium hydride, and stored over molecular sieves 4 Å under nitrogen atmosphere. Tetrahydrofuran (THF) (>98.5%, Sigma-Aldrich) was stored under argon atmosphere and over 4 Å molecular sieves. Analytical thin-layer chromatography (TLC) was performed on precoated (Merck TLC Silica Gel 60 F_254_). Visualization of the developed chromatogram was performed by UV light (254 nm) and/or using potassium permanganate stain. Flash chromatography was performed on silica gel (Merck, 230–400 mesh) with the indicated eluent. Common solvents for chromatography [dichloromethane (DCM), ethyl acetate (EtOAc), hexanes, methanol (MeOH)] were directly used from the commercial source without further purification. The nuclear magnetic resonance spectrum was recorded on a Bruker 400 MHz spectrometer. Chemical shifts for ^1^H NMR spectra are recorded in parts per million with the solvent resonance as the reference (CDCl_3_*δ* = 7.26 ppm). Data are reported as follows: chemical shift, multiplicity (s = singlet, d = doublet, t = triplet, q = quartet, m = multiplet, and brs = broad or combinations thereof), and coupling constant in Hz. Chemical shifts for ^13^C NMR spectra are recorded in parts per million using the solvent (CDCl_3_*δ* = 77.16 ppm) as the reference. High-resolution mass spectrometry experiments were operated on an Orbitrap Exploris-260 Mass Spectrometer from Thermo Scientific. Liquid chromatography/mass spectrometry (LC/MS) experiments were operated on an Agilent LC-MS single quadrupole spectrometer. Melting points were recorded on the Buchi melting point M-565 system.

### General procedure for hemiacetals: (redox-relay Heck approach)

Hemiacetals were prepared according to a known procedure^27^ with slight modifications. To a stirred solution of NaHCO_3_ (2.0 equiv.), Pd(OAc)_2_ (0.02 equiv.), *n*Bu_4_NCl (1.0 equiv.), and aryl iodide, 1 (1.0 equiv.) in MeCN (0.40 M) was added (*Z*)-but-2-ene-1,4-diol, 2 (1.2 equiv.) at room temperature. The reaction mixture was heated to 60 °C and stirred for 12 h. It was cooled to room temperature, filtered through a Celite bed and washed with EtOAc, and the filtrate was concentrated *in vacuo*. The crude obtained was diluted with water and extracted using EtOAc (3 × 20 mL). The combined organic extracts were washed with brine, dried over Na_2_SO_4_, and concentrated *in vacuo* to obtain the product. Purification by flash column chromatography with silica gel as the stationary phase and EtOAc : Petroleum ether (4 : 1) as the mobile phase afforded the corresponding hemiacetals as diastereomeric mixtures.

4-Phenyltetrahydrofuran-2-ol 3(a): isolated as a mixture of diastereomers. Colorless liquid (6.66 g, 39.8 mmol, 81%): TLC (30% EtOAc in pet-ether): *R*_f_ = 0.5; ^1^H NMR (400 MHz, CDCl_3_): *δ* 7.38–7.30 (m, 4H, Ar), 7.28–7.23 (m, 4H, Ar), 5.74–5.71 (m, 2H, Ar), 4.47 (t, *J* = 8.00 Hz, 1H, CH), 4.26–4.21 (m, 1H, CH), 4.00 (t, *J* = 8.40 Hz, 1H, CH), 3.86 (t, *J* = 8.00 Hz, 1H, CH), 3.77–3.73 (m, 1H, CH), 2.68–2.63 (m, 1H, CH), 2.41–2.36 (m, 1H, CH), 2.17–2.11 (m, 1H, CH), 2.06–2.00 (m, 1H, CH); GC-MS (EI) *m*/*z* calculated for C_10_H_12_O_2_: 164.08 (M^+^) found 164.1.

### Typical procedure for the Ugi multicomponent reaction

The Ugi multicomponent reaction was performed as per the slightly modified literature procedure.^28^ To a solution of hemiacetal, 3(a) (1.0 equiv.), isocyanide, 5 (1.0 equiv.), aryl amine, 4(i) (1.0 equiv.) in methanol (10 mL), maintained at 0 °C, BF_3_·OEt_2_ (3.0 equiv.) was added dropwise over 2 minutes and stirred for 1 h. Thereafter, water (20 mL) was added *in situ*, and the reaction mixture was stirred for 12 h at room temperature. It was concentrated *in vacuo*, and the crude obtained was diluted with water. The aqueous phase was extracted with DCM (3 × 20 mL). The combined organic extracts were washed with brine, dried over Na_2_SO_4_, and concentrated *in vacuo* to afford a pale-brown liquid. Purification was done by flash column chromatography using silica gel as the stationary phase and EtOAc : Petroleum ether (7 : 3) as the mobile phase to afford the corresponding diastereomers.

(±) *Trans*6′(a)(i), colorless gum; TLC (70% ethyl acetate in hexanes): *R*_f_ = 0.6; ^1^H NMR (400 MHz, CDCl_3_): *δ* 7.41–7.13 (m, 7H, Ar), 6.75–6.71 (m, 1H, Ar), 6.52–6.49 (m, 2H, Ar), 4.06–4.03 (m, 1H, CH), 3.80–3.72 (m, 2H, CH), 3.65 (s, 3H, OCH_3_), 3.07–2.95 (m, 1H, CH), 2.35–2.28 (m, 1H, CH), 2.20–2.13 (m, 1H, CH); ^13^C NMR (100 MHz, CDCl_3_): *δ* 174.7, 146.9, 140.2, 129.2, 128.9, 127.5, 122.1, 114.0, 67.4, 60.5, 52.1, 43.3, 35.9; LCMS (ESI) *m*/*z* calculated for C_18_H_21_NO_3_ [M + H]^+^: 300.16, found: 300.3; RT-0.989 min.

(±) *Cis*6″(a)(i), colorless gum; TLC (70% ethyl acetate in hexanes): *R*_f_ = 0.6; ^1^H NMR (400 MHz, CDCl_3_): *δ* 7.39–7.13 (m, 7H, Ar), 6.72 (t, *J* = 7.4 Hz, 1H, Ar), 6.52–6.49 (m, 2H, Ar), 3.94–3.91 (m, 1H, CH), 3.85–3.77 (m, 2H, CH), 3.65 (s, 3H, OCH_3_), 3.20–3.16 (m, 1H, CH), 2.20–2.17 (m, 2H, CH); ^13^C NMR (100 MHz, CDCl_3_): *δ* 174.2, 146.4, 140.6, 129.2, 129.0, 127.6, 126.8, 118.5, 113.5, 67.2, 52.1, 44.9, 35.5; LCMS (ESI) *m*/*z* calculated for C_18_H_21_NO_3_ [M + H]^+^: 300.16, found: 300.3; RT-0.987 min.

### Typical procedure for the synthesis of substituted pyrrolidines

To a solution of the trans isomer of the Ugi reaction product (±) 6′(a)(i) (1.0 equiv.) in DCM (20 mL) maintained at 0 °C was added DMAP (1.0 equiv.), DIPEA (1.0 equiv.) followed by tosyl chloride (1.5 equiv.). The reaction mixture was stirred for 2 h at the same temperature. Upon completion of the reaction, it was diluted with water and extracted using DCM (3 × 20 mL). The organic extracts were combined, washed with brine, dried over Na_2_SO_4_, and concentrated *in vacuo* to afford the crude product. Purification by flash column chromatography using silica gel as the stationary phase and DCM : MeOH (9 : 1) mixture as the mobile phase afforded the pure product as a racemic mixture.

#### (1) (±) *Trans* methyl 1,4-diphenylpyrrolidine-2-carboxylate, 8′(a)(i)

White solid; M. P. = 71–72 °C; TLC (25% ethyl acetate in hexanes): *R*_f_ = 0.6; ^1^H NMR (400 MHz, CDCl_3_): *δ* 7.40–7.32 (m, 5H, Ar), 7.30–7.24 (m, 2H, Ar), 6.79 (t, *J* = 0.80 Hz, 1H, Ar), 6.59 (dd, *J* = 0.80, 8.80 Hz, 2H, Ar), 4.43 (m, 1H, CH), 3.99 (t, *J* = 2.40 Hz, 1H, CH), 3.85–3.80 (m, 4H, CH), 3.42 (t, *J* = 8.80 Hz, 1H, CH), 2.54–2.44 (m, 2H, CH); ^13^C NMR (100 MHz, CDCl_3_): *δ* 174.6, 146.4, 141.1, 129.3, 128.7, 127.2, 127.0, 117.0, 111.9, 77.3, 77.2, 77.0, 76.7, 61.4, 55.3, 52.3, 42.4, 38.1; HRMS (ESI) *m*/*z* calculated for C_18_H_19_NO_2_ [M + H]^+^: 282.1489, found: 282.1486.

#### (2) (±) *Trans* methyl 1-(4-methoxyphenyl)-4-phenylpyrrolidine-2-carboxylate 8′(a)(ii)

Brown liquid; TLC (25% ethyl acetate in hexanes): *R*_f_ = 0.6; ^1^H NMR (400 MHz, CDCl_3_): *δ* 7.38–7.27 (m, 5H, Ar), 6.88–6.85 (m, 2H, Ar), 6.56–6.53 (m, 2H, Ar), 4.40–4.37 (m, 1H, CH), 3.99–3.95 (m, 1H, CH), 3.82–3.71 (m, 7H, CH), 3.16 (m, 1H, CH), 2.50–2.46 (m, 2H, CH); ^13^C NMR (100 MHz, CDCl_3_): *δ* 174.8, 151.8, 141.2, 141.1, 128.8, 128.4, 127.5, 127.2, 127.0, 125.2, 115.2, 113.8, 112.9, 61.9, 55.8, 55.9, 52.3, 44.5, 38.1; HRMS (ESI) *m*/*z* calculated for C_19_H_21_NO_3_ [M + H]^+^: 312.1594, found: 312.1592.

#### (3) (±) *Trans* methyl 1-(4-(methoxycarbonyl)phenyl)-4-phenylpyrrolidine-2-carboxylate 8′(a)(iii)

White solid; M. P. = 73–74 °C; TLC (25% ethyl acetate in hexanes): *R*_f_ = 0.6; ^1^H NMR (400 MHz, DMSO-*d*_6_): *δ* 7.81–7.78 (m, 2H, Ar), 7.41–7.30 (m, 4H, Ar), 7.29–7.25 (m, 1H, Ar), 6.60–6.58 (m, 2H, Ar), 4.63–4.59 (m, 1H, CH), 4.03–3.96 (m, 1H, CH), 3.77 (s, 3H, OCH_3_), 3.72–3.62 (m, 1H, CH), 3.61 (s, 3H, OCH_3_), 3.57–3.47 (m, 1H, CH), 3.39–2.88 (m, 1H, CH), 2.16–2.13 (m, 1H, CH); ^13^C NMR (100 MHz, DMSO-*d*_6_): *δ* 173.7, 166.7, 150.4, 141.1, 131.3, 129.0, 128.5, 127.8, 127.5, 117.9, 111.8, 60.1, 55.4, 52.7, 51.9, 42.0, 40.7, 39.8, 38.0, 37.5; HRMS (ESI) *m*/*z* calculated for C_20_H_21_NO_4_ [M + H]^+^: 340.1541, found: 340.1539.

#### (4) (±) *Trans* methyl 1-(4-fluorophenyl)-4-phenylpyrrolidine-2-carboxylate 8′(a)(iv)

Brown solid; M. P. = 87–88 °C; TLC (25% ethyl acetate in hexanes): *R*_f_ = 0.4; ^1^H NMR (400 MHz, CDCl_3_): *δ* 7.39–7.35 (m, 3H, Ar), 7.33–7.30 (m, 2H, Ar), 7.00–6.94 (m, 2H, Ar), 6.51–6.47 (m, 2H, Ar), 4.41–4.38 (m, 1H, CH), 3.97 (t, *J* = 8.00 Hz, 1H, CH), 3.82 (s, 4H, CH), 3.39 (t, *J* = 8.80 Hz, 1H, CH), 2.52–2.45 (m, 2H, CH); ^13^C NMR (100 MHz, CDCl_3_): *δ* 174.5, 156.8, 143.1, 141.0, 128.2, 128.1, 127.4, 126.6, 125.2, 116.2, 115.5, 112.3, 61.7, 58.3, 55.7, 51.3, 42.5, 38.2; ^19^F NMR (376 MHz, CDCl_3_): *δ* −117.1 (m, 1F); HRMS (ESI) *m*/*z* calculated for C_18_H_18_FNO_2_ [M + H]^+^: 300.1392, found: 300.1391.

#### (5) (±) *Trans* methyl 1-(4-bromophenyl)-4-phenylpyrrolidine-2-carboxylate 8′(a)(v)

Brown solid; M. P. = 84–85 °C; TLC (20% ethyl acetate in hexanes): *R*_f_ = 0.4; ^1^H NMR (400 MHz, CDCl_3_): *δ* 7.39–7.31 (m, 7H, Ar), 6.47–6.42 (m, 2H, Ar), 4.42–4.39 (m, 1H, CH), 3.96 (t, *J* = 8.40 Hz, 1H, CH), 3.83–3.72 (m, 4H, CH), 3.38 (t, *J* = 8.80 Hz, 1H, CH), 2.55–2.45 (m, 2H, CH); ^13^C NMR (100 MHz, CDCl_3_): *δ* 174.2, 145.4, 140.0, 133.8, 128.8, 127.9, 127.2, 126.8, 125.6, 120.5, 116.5, 113.4, 61.3, 58.4, 52.4, 49.4, 42.3, 38.0; HRMS (ESI) *m*/*z* calculated for C_18_H_18_BrNO_2_ [M + H]^+^: 360.0594, found: 360.0591.

#### (6) (±) *Trans* methyl 1-(4-cyanophenyl)-4-phenylpyrrolidine-2-carboxylate 8′(a)(vi)

White solid; M. P. = 77–78 °C; TLC (25% ethyl acetate in hexanes): *R*_f_ = 0.6; ^1^H NMR (400 MHz, CDCl_3_): *δ* 7.53–7.50 (m, 2H, Ar), 7.40–7.37 (m, 2H, Ar), 7.33–7.29 (m, 3H, Ar), 6.56–6.54 (m, 2H, Ar), 4.52–4.49 (m, 1H, CH), 4.02 (t, *J* = 0.80 Hz, 1H, CH), 3.82 (s, 3H, OCH_3_), 3.78–3.75 (m, 1H, CH), 3.46 (t, *J* = 9.60 Hz, 1H, CH), 2.56–2.52 (m, 2H, CH); ^13^C NMR (100 MHz, CDCl_3_): *δ* 173.2, 149.0, 133.7, 133.6, 129.0, 128.9, 128.8, 127.3, 125.8, 120.4, 112.9, 112.1, 111.9, 61.0, 60.8, 55.4, 52.6, 43.3, 38.4; HRMS (ESI) *m*/*z* calculated for C_19_H_18_N_2_O_2_ [M + H]^+^: 307.1438, found: 307.1438.

#### (7) (±) *Trans* methyl 4-phenyl-1-(4-(trifluoromethyl) phenyl) pyrrolidine-2-carboxylate 8′(a)(vii)

White solid; M. P. = 80–81 °C; TLC (20% ethyl acetate in hexanes): *R*_f_ = 0.5; ^1^H NMR (400 MHz, CDCl_3_): *δ* 7.50–7.48 (m, 2H, Ar), 7.40–7.37 (m, 2H, Ar), 7.34–7.29 (m, 3H, Ar), 6.60–6.58 (m, 2H, Ar), 4.51–4.48 (m, 1H, CH), 3.98 (t, *J* = 5.80 Hz, 1H, CH), 3.84–3.76 (m, 4H, CH), 3.46 (t, *J* = 9.20 Hz, 1H, CH), 2.58–2.48 (m, 2H, CH); ^13^C NMR (100 MHz, CDCl_3_): *δ* 173.8, 148.5, 140.5, 129.1, 128.8, 127.2, 126.7, 126.6, 126.4, 123.7, 118.8, 111.3, 61.1, 58.4, 55.2, 52.5, 42.2, 38.0; ^19^F NMR (376 MHz, CDCl_3_): *δ* −61.7 (3F); HRMS (ESI) *m*/*z* calculated for C_19_H_18_F_3_NO_2_ [M + H]^+^: 350.1360, found: 350.1363.

#### (8) (±) *Trans* methyl 1-mesityl-4-phenylpyrrolidine-2-carboxylate 8′(a)(viii)

Colorless gum; TLC (25% ethyl acetate in hexanes): *R*_f_ = 0.5; ^1^H NMR (400 MHz, CDCl_3_): *δ* 7.41–7.32 (m, 4H, Ar), 7.26–7.24 (m, 1H, Ar), 6.89–6.87 (m, 2H, Ar), 4.25 (d, *J* = 8.40 Hz, 1H, CH), 3.91–3.82 (m, 1H, CH), 3.72–3.63 (m, 4H, CH), 3.24 (t, *J* = 8.40 Hz, 1H, CH), 2.58–2.51 (m, 2H, CH), 2.34 (s, 6H, CH), 2.27 (s, 3H, CH); ^13^C NMR (100 MHz, CDCl_3_): *δ* 175.4, 141.4, 141.1, 135.4, 133.4, 131.9, 129.6, 128.5, 127.3, 127.1, 126.6, 125.9, 63.4, 58.6, 55.3, 51.7, 43.9, 39.8, 21.4, 20.9, 20.3; HRMS (ESI) *m*/*z* calculated for C_21_H_25_NO_2_ [M + H]^+^: 324.1955, found: 324.1956.

#### (9) (±) *Trans* methyl 1-(3,4-dichlorophenyl)-4-phenylpyrrolidine-2-carboxylate 8′(a)(ix)

Pale brown gum; TLC (20% ethyl acetate in hexanes): *R*_f_ = 0.4; ^1^H NMR (400 MHz, CDCl_3_): *δ* 7.40–7.29 (m, 5H, Ar), 7.25 (t, *J* = 4.40 Hz, 1H, Ar), 6.66–6.64 (m, 1H, Ar), 6.39 (m, 1H, Ar), 4.42–4.39 (m, 1H, CH), 3.94 (t, *J* = 8.40 Hz, 1H, CH), 3.86–3.76 (m, 3H, OCH_3_), 3.55–3.53 (m, 1H, CH), 2.90–2.87 (m, 1H, CH), 2.54–2.49 (m, 1H, CH), 2.35–2.30 (m, 1H, CH); ^13^C NMR (100 MHz, CDCl_3_): *δ* 174.0, 146.0, 139.9, 131.3, 129.1, 128.9, 127.3, 127.2, 126.8, 126.0, 125.3, 119.9, 119.8, 111.4, 51.5, 38.5, 38.0; LCMS (ESI): *m*/*z* calculated for C_18_H_17_Cl_2_NO_2_ [M + H]^+^: 350.07, found: 350.3.

#### (10) (±) *Trans* methyl 4-phenyl-1-(quinolin-8-yl) pyrrolidine-2-carboxylate 8′(a)(x)

Pale green gum; TLC (50% ethyl acetate in hexanes): *R*_f_ = 0.2; ^1^H NMR (400 MHz, CDCl_3_): *δ* 8.70–8.60 (m, 1H, Ar), 8.07–8.03 (m, 1H, Ar), 7.44–7.36 (m, 4H, Ar), 7.33–7.27 (m, 2H, Ar), 7.21–7.16 (m, 1H, Ar), 6.91–6.81 (m, 1H, Ar), 5.86–5.32 (m, 1H, Ar), 4.08–4.04 (m, 1H, CH), 3.98–3.91 (m, 1H, CH), 3.86 (s, 3H, OCH_3_), 3.63 (t, *J* = 9.60 Hz, 1H, CH), 3.54 (s, 1H, CH), 2.93–2.26 (m, 2H, CH); ^13^C NMR (100 MHz, CDCl_3_): *δ* 175.6, 146.7, 144.8, 139.9, 138.6, 129.9, 128.7, 127.7, 127.0, 121.5, 120.7, 117.0, 116.4, 111.7, 68.5, 65.6, 64.2, 51.8, 42.9, 38.8, 38.5; HRMS (ESI) *m*/*z* calculated for C_21_H_20_N_2_O_2_ [M + H]^+^: 333.1595, found: 333.1592.

#### (11) (±) *Trans* methyl 1-(naphthalen-1-yl)-4-phenylpyrrolidine-2-carboxylate 8′(a)(xi)

White solid; M. P. = 80–81 °C; TLC (20% ethyl acetate in hexanes): *R*_f_ = 0.5; ^1^H NMR (400 MHz, CDCl_3_): *δ* 8.31 (d, *J* = 7.60 Hz, 1H, Ar), 7.85 (d, *J* = 2.00 Hz, 1H, Ar), 7.58 (d, *J* = 8.00 Hz, 1H, Ar), 7.51–7.48 (m, 2H, Ar), 7.45–7.40 (m, 1H, Ar), 7.37–7.32 (m, 4H, Ar), 7.27–7.23 (m, 2H, Ar), 4.73–4.70 (m, 1H, CH), 4.35 (t, *J* = 7.60 Hz, 1H, CH), 3.85 (t, *J* = 7.20 Hz, 1H, CH), 3.58 (m, 3H, OCH_3_), 3.34–3.30 (m, 1H, CH), 2.74–2.68 (m, 1H, CH), 2.59–2.52 (m, 1H, CH); ^13^C NMR (100 MHz, CDCl_3_): *δ* 173.9, 145.1, 143.6, 135.7, 134.9, 129.6, 129.0, 128.6, 128.3, 127.2, 126.6, 125.8, 125.2, 124.2, 123.6, 115.8, 63.5, 60.5, 51.8, 42.9, 38.4; HRMS (ESI) *m*/*z* calculated for C_22_H_21_NO_2_ [M + H]^+^: 332.1642, found: 332.1641.

#### (12) (±) *Trans* methyl 1-(2,3-dihydro-1*H*-inden-4-yl)-4-phenylpyrrolidine-2-carboxylate 8′(a)(xii)

White solid; M. P. = 73–74 °C; TLC (20% ethyl acetate in hexanes): *R*_f_ = 0.5; ^1^H NMR (400 MHz, CDCl_3_): *δ* 7.39–7.35 (m, 3H, Ar), 7.32–7.25 (m, 2H, Ar), 7.13 (d, *J* = 8.4 Hz, 1H, Ar), 6.50 (d, *J* = 2.00 Hz, 1H, Ar), 6.40–6.38 (m, 1H, Ar), 4.43–4.41 (m, 1H, CH), 3.99 (t, *J* = 8.40 Hz, 1H, CH), 3.82 (s, 3H, OCH_3_), 3.76 (m, 1H, CH), 3.40 (t, *J* = 9.20 Hz, 1H, CH), 3.01–2.82 (m, 4H, CH), 2.51–2.41 (m, 2H, CH), 2.10 (t, *J* = 7.20 Hz, 2H, CH); ^13^C NMR (100 MHz, CDCl_3_): *δ* 175.0, 145.6, 145.4, 142.6, 136.2, 128.7, 128.2, 126.9, 124.2, 124.1, 110.0, 61.7, 56.6, 52.3, 42.4, 38.3, 33.4, 32.4, 31.9, 28.4, 25.6; HRMS (ESI) *m*/*z* calculated for C_21_H_23_NO_2_ [M + H]^+^: 322.1799, found: 322.1795.

#### (13) (±) *Trans* methyl 4-(4-fluorophenyl)-1-phenylpyrrolidine-2-carboxylate 8′(b)(i)

Pale green gum; TLC (50% ethyl acetate in hexanes): *R*_f_ = 0.2; ^1^H NMR (400 MHz, CDCl_3_): *δ* 7.30–7.25 (m, 3H, Ar), 7.09–7.03 (m, 2H, Ar), 6.78 (t, *J* = 0.80 Hz, 2H, Ar), 6.60–6.57 (m, 2H, Ar), 4.46–4.43 (m, 1H, CH), 3.99 (t, *J* = 8.40 Hz, 1H, CH), 3.81 (m, 4H, CH), 3.38 (t, *J* = 9.20 Hz, 1H, CH), 2.53–2.39 (m, 2H, CH); ^13^C NMR (100 MHz, CDCl_3_): *δ* 174.6, 163.0, 146.4, 136.7, 129.4, 128.7, 128.1, 126.8, 126.3, 117.1, 116.0, 115.6, 111.9, 61.3, 55.3, 52.3, 41.7, 38.2; ^19^F NMR (376 MHz, CDCl_3_): *δ* −115.0 (m, 1F); HRMS (ESI) *m*/*z* calculated for C_18_H_18_FNO_2_ [M + H]^+^: 300.1392, found: 300.1390.

#### (14) (±) *Trans* methyl 4-(4-fluorophenyl)-1-(4-methoxyphenyl)pyrrolidine-2-carboxylate 8′(b)(ii)

Brown liquid; TLC (30% ethyl acetate in hexanes): *R*_f_ = 0.4; ^1^H NMR (400 MHz, CDCl_3_): *δ* 7.33–7.24 (m, 2H, Ar), 7.09–7.03 (m, 2H, Ar), 6.78–6.76 (m, 2H, Ar), 6.60–6.57 (m, 2H, Ar), 4.38–4.36 (m, 1H, CH), 3.96 (t, *J* = 8.4 Hz, 1H, CH), 3.81 (s, 3H, OCH_3_), 3.77 (s, 3H, OCH_3_), 3.43–3.40 (m, 1H, CH), 2.89–2.81 (m, 1H, CH), 2.52–2.43 (m, 2H, CH); ^13^C NMR (100 MHz, CDCl_3_): *δ* 174.8, 160.6, 151.7, 141.2, 137.0, 128.7, 128.6, 127.5, 115.6, 115.4, 115.1, 113.8, 112.7, 61.7, 55.9, 55.5, 52.3, 41.9, 38.3; ^19^F NMR (376 MHz, CDCl_3_): *δ* −115.6 (m, 1F); HRMS (ESI) *m/z* calculated for C_19_H_20_FNO_3_ [M + H]^+^: 330.1497, found: 330.1494.

#### (15) (±) *Trans* methyl 4-(4-fluorophenyl)-1-(4-(methoxycarbonyl)phenyl)pyrrolidine-2-carboxylate 8′(b)(iii)

White solid; M. P. = 77–78 °C TLC (30% ethyl acetate in hexanes): *R*_f_ = 0.4; ^1^H NMR (400 MHz, CDCl_3_): *δ* 7.96–7.92 (m, 2H, Ar), 7.32–7.26 (m, 2H, Ar), 7.08–7.02 (m, 2H, Ar), 6.56–6.53 (m, 2H, Ar), 4.56–4.51 (m, 1H, CH), 4.03–3.94 (m, 1H, CH), 3.91 (s, 3H, CH), 3.81 (s, 2H, CH), 3.76 (s, 1H, CH), 3.73–3.69 (m, 1H, CH), 3.43 (t, *J* = 9.20 Hz, 1H, CH), 2.54–2.49 (m, 2H, CH); ^13^C NMR (100 MHz, CDCl_3_): *δ* 173.8, 167.3, 149.8, 149.6, 136.1, 131.5, 128.8, 128.7, 128.6, 118.4, 115.8, 115.6, 115.5, 111.3, 61.0, 55.5, 55.2, 52.5, 41.5, 38.3; ^19^F NMR (376 MHz, CDCl_3_): *δ* −114.6 (m, 1F); HRMS (ESI) *m*/*z* calculated for C_20_H_20_FNO_4_ [M + H]^+^: 358.1446, found: 358.1444.

#### (16) (±) *Trans* methyl 1-(4-bromophenyl)-4-(4-fluorophenyl) pyrrolidine-2-carboxylate 8′(b)(v)

White solid; M. P. = 80–81 °C TLC (30% ethyl acetate in hexanes): *R*_f_ = 0.4; ^1^H NMR (400 MHz, CDCl_3_): *δ* 7.35–7.32 (m, 2H, Ar), 7.31–7.24 (m, 2H, Ar), 7.17–7.02 (m, 2H, Ar), 6.46–6.42 (m, 2H, Ar), 4.40–4.38 (m, 1H, CH), 3.93 (t, *J* = 8.40 Hz, 1H, CH), 3.82 (s, 3H, CH), 3.79 (s, 1H, CH), 3.33 (t, *J* = 9.20 Hz, 1H, CH), 2.53–2.40 (m, 2H, CH); ^13^C NMR (100 MHz, CDCl_3_): *δ* 174.1, 163.1, 160.6, 145.3, 136.4, 132.0, 128.6, 127.9, 126.3, 116.4, 115.6, 113.5, 109.1, 61.2, 55.4, 52.4, 41.7, 38.1; ^19^F NMR (376 MHz, CDCl_3_): *δ* −114.7 (m, 1F); HRMS (ESI) *m*/*z* calculated for C_18_H_17_BrFNO_2_ [M + H]^+^: 378.0497, found: 378.0495.

#### (17) (±) *Trans* methyl 4-(4-bromophenyl)-1-phenylpyrrolidine-2-carboxylate 8′(c)(i)

White solid; M. P. = 79–80 °C TLC (40% ethyl acetate in hexanes): *R*_f_ = 0.3; ^1^H NMR (400 MHz, CDCl_3_): *δ* 7.50 (d, *J* = 3.20 Hz, 2H, Ar), 7.30–7.21 (m, 2H, Ar), 7.18 (d, *J* = 3.20 Hz, 2H, Ar), 6.77 (t, *J* = 1.20 Hz, 1H, Ar), 6.58 (d, *J* = 1.20 Hz, 2H, Ar), 4.46–4.42 (m, 1H, CH), 3.98 (t, *J* = 11.20 Hz, 1H, CH), 3.81 (m, 4H, CH), 3.40 (t, *J* = 8.4 Hz, 1H, CH), 2.51–2.36 (m, 2H); ^13^C NMR (100 MHz, CDCl_3_): *δ* 174.5, 146.3, 140.1, 131.8, 129.4, 128.9, 126.9, 126.7, 122.6, 120.8, 117.1, 115.1, 111.9, 61.2, 55.0, 52.4, 41.9, 38.0; LCMS (ESI) *m*/*z* calculated for C_18_H_18_BrNO_2_ [M + 2H]^+^: 362.2 found: 362.0.

#### (18) (±) *Trans* methyl 4-(4-bromophenyl)-1-(4-fluorophenyl) pyrrolidine-2-carboxylate 8′(c)(iv)

Off white solid; M. P. = 78–79 °C TLC (40% ethyl acetate in hexanes): *R*_f_ = 0.3; ^1^H NMR (400 MHz, CDCl_3_): *δ* 7.49 (d, *J* = 3.20 Hz, 2H, Ar), 7.18 (d, *J* = 3.20 Hz, 2H, Ar), 6.96 (t, *J* = 4.80 Hz, 2H, Ar), 6.52–6.47 (m, 2H, Ar), 4.39 (d, *J* = 2.80 Hz, 1H, CH), 3.79–3.71 (m, 1H, CH), 3.94 (t, *J* = 10.80 Hz, 1H, CH), 3.80 (s, 3H, OCH_3_), 3.34 (t, *J* = 12.00 Hz, 1H, CH), 2.53–2.37 (m, 2H, CH); ^13^C NMR (100 MHz, CDCl_3_): *δ* 174.4, 142.9, 140.0, 132.4, 131.8, 128.9, 121.2, 120.8, 116.0, 115.7, 112.5, 112.4, 76.6, 61.6, 55.5, 52.4, 42.0, 38.0; ^19^F NMR (376 MHz, CDCl_3_): *δ* −123.7 (m, 1F); LCMS (ESI) *m*/*z* calculated for C_18_H_17_BrFNO_2_ [M + 2H]^+^: 380.2 found: 380.0.

#### (19) (±) *Trans* methyl 1-phenyl-4-(thiophen-2-yl) pyrrolidine-2-carboxylate 8′(d)(i)

Colorless liquid; TLC (40% ethyl acetate in hexanes): *R*_f_ = 0.5; ^1^H NMR (400 MHz, CDCl_3_): *δ* 7.27–7.21 (m, 3H, Ar), 7.01–6.96 (m, 2H, Ar), 6.77 (t, *J* = 9.60 Hz, 1H, Ar), 6.58 (m, 2H, Ar), 4.51–4.46 (m, 1H, CH), 3.95–3.92 (m, 1H, CH), 3.82–3.74 (m, 2H, CH), 3.73–3.71 (m, 3H, CH), 2.94–2.90 (m, 1H, CH), 2.36–2.32 (m, 1H, CH); ^13^C NMR (100 MHz, CDCl_3_): *δ* 174.5, 146.4, 144.1, 129.3, 126.6, 124.2, 123.7, 117.2, 112.0, 77.0, 61.0, 55.9, 52.3, 39.3, 38.5, 29.7; HRMS (ESI) *m*/*z* calculated for C_16_H_17_NO_2_S [M + H]^+^: 288.1050, found: 288.1049.

#### (20) (±) *Trans* methyl 1-(4-methoxyphenyl)-4-(thiophen-2-yl)pyrrolidine-2-carboxylate 8′(d)(ii)

Brown liquid; TLC (30% ethyl acetate in hexanes): *R*_f_ = 0.4; ^1^H NMR (400 MHz, CDCl_3_): *δ* 7.28–7.20 (m, 1H, Ar), 6.99–6.94 (m, 2H, Ar), 6.86 (d, *J* = 5.20 Hz, 2H, Ar), 6.54 (d, *J* = 3.20 Hz, 2H, Ar), 4.38–4.34 (m, 1H, CH), 4.01–3.96 (m, 2H, CH), 3.79 (s, 3H, OCH_3_), 3.78 (s, 3H, OCH_3_), 3.43–3.37 (m, 1H, CH), 2.55–2.46 (m, 2H, CH); ^13^C NMR (100 MHz, CDCl_3_): *δ* 174.7, 151.8, 144.8, 141.1, 127.7, 126.9, 124.1, 123.6, 122.8, 119.1, 116.0, 113.8, 112.7, 61.6, 55.9, 52.3, 38.3; HRMS (ESI) *m*/*z* calculated for C_17_H_19_NO_3_S [M + H]^+^: 318.1156, found: 318.1155.

#### (21) (±) *Cis* methyl 1,4-diphenylpyrrolidine-2-carboxylate 8″(a)(i)

Pale yellow solid; M. P. = 78–79 °C; TLC (20% ethyl acetate in hexanes): *R*_f_ = 0.5; ^1^H NMR (400 MHz, CDCl_3_): *δ* 7.40–7.34 (m, 4H, Ar), 7.31–7.26 (m, 1H, Ar), 7.26–7.23 (m, 2H, Ar), 6.76 (t, *J* = 7.60 Hz, 1H, Ar), 6.59 (d, *J* = 0.80 Hz, 2H, Ar), 4.50 (t, *J* = 8.00 Hz, 1H, CH), 3.92 (t, *J* = 8.80 Hz, 1H, CH), 3.72–3.67 (m, 4H, CH), 3.54–3.49 (m, 1H, CH), 2.90–2.83 (m, 1H, CH), 2.36–2.28 (m, 1H, CH); ^13^C NMR (100 MHz, CDCl_3_): *δ* 174.8, 146.6, 140.6, 129.3, 128.7, 127.4, 127.0, 117.1, 112.1, 77.4, 77.2, 77.0, 76.6, 61.1, 55.8, 52.2, 43.5, 38.6; HRMS (ESI) *m*/*z* calculated for C_18_H_19_NO_2_ [M + H]^+^: 282.1486, found: 282.1485.

#### (22) (±) *Cis* methyl 1-(4-bromophenyl)-4-phenylpyrrolidine-2-carboxylate 8″(a)(v)

Pale yellow solid; M. P. = 81–82 °C; TLC (30% ethyl acetate in hexanes): *R*_f_ = 0.5; ^1^H NMR (400 MHz, CDCl_3_): *δ* 7.40–7.28 (m, 7H, Ar), 6.45 (d, *J* = 2.80 Hz, 2H, Ar), 4.46 (t, *J* = 8.00 Hz, 1H, CH), 3.87–3.80 (m, 1H, CH), 3.72 (s, 3H, OCH_3_), 3.68–3.63 (m, 1H, CH)), 3.55–3.49 (m, 1H, CH), 2.91–2.84 (m, 1H, CH), 2.33–2.27 (m, 1H, CH); ^13^C NMR (100 MHz, CDCl_3_): *δ* 174.4, 145.5, 140.3, 131.9, 128.8, 128.7, 127.3, 127.1, 113.7, 109.1, 76.6, 61.3, 61.1, 55.8, 52.4, 43.4, 42.4, 38.6; HRMS (ESI) *m*/*z* calculated for C_18_H_18_BrNO_2_ [M + H]^+^: 360.0594, found: 360.0590.

#### Synthesis of (±) *trans* methyl 1-(2′,4′-difluoro-[1,1′-biphenyl]-4-yl)-4-phenylpyrrolidine-2-carboxylate (9)

To a solution of (±) 8′(a)(v) (1.0 equiv.), (2,4-difluorophenyl) boronic acid (1.1 equiv.), in dioxane-water mixture (1 : 1 v/v, 10 mL) were added Pd(dppf)Cl_2_.DCM (5 mol%) and K_3_PO_4_ (1.5 equiv.). The reaction mixture was degassed for 5 min under N_2_ atmosphere and stirred for 3 h at 80 °C. It was cooled, filtered through a Celite bed, and washed with EtOAc. The filtrate was washed with brine, dried over Na_2_SO_4_ and concentrated *in vacuo* to afford a gummy solid. Purification was done by flash column chromatography using silica gel as the stationary phase and DCM : MeOH (9.5 : 0.5) as the mobile phase to afford the pure product. Yield: 82%; white solid; M. P. = 87–88 °C; TLC (40% ethyl acetate in hexanes): *R*_f_ = 0.3; ^1^H NMR (400 MHz, CDCl_3_): *δ* 7.43–7.28 (m, 8H, Ar), 6.93–6.86 (m, 2H, Ar), 6.65–6.64 (m, 2H, Ar), 4.04–4.02 (m, 1H, CH), 3.92 (t, *J* = 8.40 Hz, 1H, CH), 3.80 (s, 3H, CH), 3.78 (m, 1H, CH), 2.64–2.50 (m, 2H, CH), 1.87–1.85 (m, 1H, CH); LCMS (ESI) *m*/*z* calculated for C_24_H_21_F_2_NO_2_ [M + H]^+^: 394.16 found: 394.21.

#### Synthesis of (±) *trans N*-benzyl-1-(4-fluorophenyl)-4-phenylpyrrolidine-2-carboxamide (11)

To a solution of (±) 8′(a)(iv) (1.0 equiv.), in THF-water (1 : 1 v/v, 10 mL) was added LiOH (3.0 equiv.) and the reaction mixture was stirred for 5 h at 25 °C. It was concentrated and acidified with 1.5N HCl (pH = 4) and extracted with EtOAc. The organic extracts were washed with brine, dried over Na_2_SO_4_, and concentrated *in vacuo* to afford the carboxylic acid 10 as a gummy solid. To a solution of the carboxylic acid (1.0 equiv.) in DMF (20 mL) was added benzyl amine (1.05 equiv.), DIPEA (2.0 equiv.) and HATU (1.5 equiv.). The reaction mixture was stirred for 8 h at 25 °C and then quenched with ice. The aqueous phase was extracted with DCM (3 × 20 mL). The combined organic extracts were washed with brine, dried over Na_2_SO_4_, and concentrated *in vacuo* to afford a colorless gum. Purification was done by flash column chromatography using silica gel as the stationary phase and DCM : MeOH (9.5 : 0.5) as the mobile phase to afford the pure product. Yield: 71%; white solid; M. P. = 81–82 °C; TLC (40% ethyl acetate in hexanes): *R*_f_ = 0.4; ^1^H NMR (400 MHz, CDCl_3_): *δ* 7.39–7.27 (m, 6H, Ar), 7.25–7.23 (m, 3H, Ar), 7.10–7.01 (m, 1H, Ar), 6.98 (t, *J* = 8.40 Hz, 2H, Ar), 6.61–6.57 (m, 2H, Ar), 4.59–4.48 (m, 2H, CH), 4.21 (d, *J* = 8.80 Hz, 1H, CH), 3.95–3.91 (m, 1H, CH), 3.68–3.51 (m, 3H, OCH_3_), 2.71–2.66 (m, 1H, CH), 2.55–2.47 (m, 1H, CH); LCMS (ESI) *m*/*z* calculated for C_24_H_23_FN_2_O [M + H]^+^: 375.18 found: 375.20.

## Conclusion

An efficient and concise Ugi multicomponent strategy for the synthesis of functionalized pyrrolidine derivatives using hemiacetals as aldehyde surrogates was developed. This approach circumvents conventional multistep synthetic routes and enables rapid access to structurally diverse pyrrolidine frameworks under mild conditions. Subsequent scaffold modifications further expand the chemical space, allowing the introduction of varied functional groups with high structural fidelity. The operational simplicity, modularity, and broad substrate scope of this methodology highlight its utility in complex molecule construction. Overall, this work underscores the potential of Ugi multicomponent reaction as a powerful tool for the rapid generation of bioactive, functionally rich heterocycles relevant to medicinal and chemical biology applications.

## Author contributions

S. K. P., investigation; P. S. S., conceptualization; T. K. A., data curation; V. A. N., reviewing and editing.

## Conflicts of interest

There are no conflicts of interest to declare.

## Supplementary Material

RA-016-D6RA07669F-s001

## Data Availability

The data supporting this article have been included as part of the supplementary information (SI). Supplementary information: analytical data and ^1^H, ^13^C, ^19^F NMR and mass spectra of the synthesised compounds. See DOI: https://doi.org/10.1039/d6ra07669f.
