# Supplementary material for: Facile synthesis of trans racemic 1,4-diaryl substituted, proline esters via Ugi multicomponent reaction
Source: RSC Adv. 2026 Oct 6;16(46):60292–302. doi: 10.1039/d6ra07669f (PMC13639724; doi:10.1039/d6ra07669f)
Supplement: Supplementary file 1 [file RA-016-D6RA07669F-s001.pdf]

Supplementary Information (SI) for RSC Advances.
This journal is © The Royal Society of Chemistry 2026
Supplementary Information (SI) for RSC Advances.
This journal is © The Royal Society of Chemistry

Facile Synthesis of Trans Racemic 1,4-Diaryl Substituted Proline Esters via Ugi Multicomponent Reaction

Siva Krishna Polineni, P. Sathya Shanker, Tony Kurissery Anthappan and Vipin A. Nair *

[a] *Syngene International Limited. Biocon Park, Bommasandra IV Phase, Jigani Link Road, Bangalore 560099, Karnataka, India.*
[b]*Department of Chemistry, REVA University, Kattigenahalli, Yelahanka, Bangalore 560064, Karnataka, India.*
[c]*School of Biotechnology, Amrita Vishwa Vidyapeetham, Amritapuri Campus, Clappana, Kollam 690525, Kerala, India.*

Supplementary Information

No.	Content	Page
1	**Figure S1.** ^{1}H NMR spectrum of ***(±) trans* 6'(a)(i)**	3
2	**Figure S2.** ^{13}C NMR spectrum of ***(±) trans* 6'(a)(i)**	4
3	**Figure S3.** ^{1}H NMR spectrum of ***(±) cis* 6''(a)(i)**	5
4	**Figure S4.** ^{13}C NMR spectrum of ***(±) cis* 6''(a)(i)**	6
5	**Figure S5.** ^{1}H NMR spectrum of ***(±) trans* 8'(a)(i)**	7
6	**Figure S6.** ^{13}C NMR spectrum of ***(±) trans* 8'(a)(i)**	8
7	**Figure S7.** NOESY spectrum of ***(±) trans* 8'(a)(i)**	9
8	**Figure S8.** HRMS data of ***(±) trans* 8'(a)(i)**	10
9	**Figure S9.** ^{1}H NMR spectrum of ***(±) trans* 8'(a)(ii)**	11
10	**Figure S10.** ^{13}C NMR spectrum of ***(±) trans* 8'(a)(ii)**	12
11	**Figure S11.** HRMS data of ***(±) trans* 8'(a)(ii)**	13
12	**Figure S12.** ^{1}H NMR spectrum of ***(±) trans* 8'(a)(iii)**	14
13	**Figure S13.** ^{13}C NMR spectrum of ***(±) trans* 8'(a)(iii)**	15
14	**Figure S14.** HRMS data of ***(±) trans* 8'(a)(iii)**	16
15	**Figure S15.** ^{1}H NMR spectrum of ***(±) trans* 8'(a)(iv)**	17
16	**Figure S16.** ^{13}C NMR spectrum of ***(±) trans* 8'(a)(iv)**	18
17	**Figure S17.** ^{19}F NMR spectrum of ***(±) trans* 8'(a)(iv)**	19
18	**Figure S18.** HRMS data of ***(±) trans* 8'(a)(iv)**	20
19	**Figure S19.** ^{1}H NMR spectrum of ***(±) trans* 8'(a)(v)**	21
20	**Figure S20.** ^{13}C NMR spectrum of ***(±) trans* 8'(a)(v)**	22
21	**Figure S21.** HRMS data of ***(±) trans* 8'(a)(v)**	23
22	**Figure S22.** ^{1}H NMR spectrum of ***(±) trans* 8'(a)(vi)**	24
23	**Figure S23.** ^{13}C NMR spectrum of ***(±) trans* 8'(a)(vi)**	25
24	**Figure S24.** HRMS data of ***(±) trans* 8'(a)(vi)**	26
25	**Figure S25.** ^{1}H NMR spectrum of ***(±) trans* 8'(a)(vii)**	27
26	**Figure S26.** ^{13}C NMR spectrum of ***(±) trans* 8'(a)(vii)**	28
27	**Figure S27.** ^{19}F NMR spectrum of ***(±) trans* 8'(a)(vii)**	29
28	**Figure S28.** HRMS data of ***(±) trans* 8'(a)(vii)**	30
29	**Figure S29.** ^{1}H NMR spectrum of ***(±) trans* 8'(a)(viii)**	31
30	**Figure S30.** ^{13}C NMR spectrum of ***(±) trans* 8'(a)(viii)**	32
31	**Figure S31.** HRMS data of ***(±) trans* 8'(a)(viii)**	33
32	**Figure S32.** ^{1}H NMR spectrum of ***(±) trans* 8'(a)(ix)**	34
33	**Figure S33.** ^{13}C NMR spectrum of ***(±) trans* 8'(a)(ix)**	35
34	**Figure S34.** LC-MS data of ***(±) trans* 8'(a)(ix)**	36
35	**Figure S35.** ^{1}H NMR spectrum of ***(±) trans* 8'(a)(x)**	37

36	**Figure S36.** ^{13}C NMR spectrum of ***(±) trans*** **8’(a)(x)**	38
37	**Figure S37.** HRMS data of ***(±) trans*** **8’(a)(x)**	39
38	**Figure S38.** ^{1}H NMR spectrum of ***(±) trans*** **8’(a)(xi)**	40
39	**Figure S39.** ^{13}C NMR spectrum of ***(±) trans*** **8’(a)(xi)**	41
40	**Figure S40.** HRMS data of ***(±) trans*** **8’(a)(xi)**	42
41	**Figure S41.** ^{1}H NMR spectrum of ***(±) trans*** **8’(a)(xii)**	43
42	**Figure S42.** ^{13}C NMR spectrum of ***(±) trans*** **8’(a)(xii)**	44
43	**Figure S43.** HRMS data of ***(±) trans*** **8’(a)(xii)**	45
44	**Figure S44.** ^{1}H NMR spectrum of ***(±) trans*** **8’(b)(i)**	46
45	**Figure S45.** ^{13}C NMR spectrum of ***(±) trans*** **8’(b)(i)**	47
46	**Figure S46.** ^{19}F NMR spectrum of ***(±) trans*** **8’(b)(i)**	48
47	**Figure S47.** HRMS data of ***(±) trans*** **8’(b)(i)**	49
48	**Figure S48.** ^{1}H NMR spectrum of ***(±) trans*** **8’(b)(ii)**	50
49	**Figure S49.** ^{13}C NMR spectrum of ***(±) trans*** **8’(b)(ii)**	51
50	**Figure S50.** ^{19}F NMR spectrum of ***(±) trans*** **8’(b)(ii)**	52
51	**Figure S51.** HRMS data of ***(±) trans*** **8’(b)(ii)**	53
52	**Figure S52.** ^{1}H NMR spectrum of ***(±) trans*** **8’(b)(iii)**	54
53	**Figure S53.** ^{13}C NMR spectrum of ***(±) trans*** **8’(b)(iii)**	55
54	**Figure S54.** ^{19}F NMR spectrum of ***(±) trans*** **8’(b)(iii)**	56
55	**Figure S55.** HRMS data of ***(±) trans 8’*** **(b)(iii)**	57
56	**Figure S56.** ^{1}H NMR spectrum of ***(±) trans*** **8’(b)(v)**	58
57	**Figure S57.** ^{13}C NMR spectrum of ***(±) trans*** **8’(b)(v)**	59
58	**Figure S58.** ^{19}F NMR spectrum of ***(±) trans*** **8’(b)(v)**	60
59	**Figure S59.** HRMS data of ***(±) trans*** **8’(b)(v)**	61
60	**Figure S60.** ^{1}H NMR spectrum of ***(±) trans*** **8’(c)(i)**	62
61	**Figure S61.** ^{13}C NMR spectrum of ***(±) trans 8’*** **(c)(i)**	63
62	**Figure S62.** LC-MS data of ***(±) trans*** **8’(c)(i)**	64
63	**Figure S63.** ^{1}H NMR spectrum of ***(±) trans*** **8’(c)(iv)**	65
64	**Figure S64.** ^{13}C NMR spectrum of ***(±) trans*** **8’(c)(iv)**	66
65	**Figure S65.** ^{19}F NMR spectrum of ***(±) trans*** **8’(c)(iv)**	67
66	**Figure S66.** LC-MS data of ***(±) trans*** **8’(c)(iv)**	68
67	**Figure S67.** ^{1}H NMR spectrum of ***(±) trans*** **8’(d)(i)**	69
68	**Figure S68.** ^{13}C NMR spectrum of ***(±) trans*** **8’(d)(i)**	70
69	**Figure S69.** HRMS data of ***(±) trans*** **8’(d)(i)**	71
70	**Figure S70.** ^{1}H NMR spectrum of ***(±) trans*** **8’(d)(ii)**	72
71	**Figure S71.** ^{13}C NMR spectrum of ***(±) trans*** **8’(d)(ii)**	73
72	**Figure S72.** HRMS data of ***(±) trans*** **8’(d)(ii)**	74
73	**Figure S73.** ^{1}H NMR spectrum of ***(±) cis*** **8’’(a)(i)**	75
74	**Figure S74.** ^{13}C NMR spectrum of ***(±) cis*** **8’’(a)(i)**	76
75	**Figure S75.** HRMS data of ***(±) cis*** **8’’(a)(i)**	77
76	**Figure S76.** ^{1}H NMR spectrum of ***(±) cis*** **8’’(a)(v)**	78
77	**Figure S77.** ^{13}C NMR spectrum of ***(±) cis*** **8’’(a)(v)**	79
78	**Figure S78.** HRMS data of ***(±) cis*** **8’’(a)(v)**	80
79	**Figure S79.** ^{1}H NMR spectrum of ***(±) trans (9)***	81
80	**Figure S80.** LC-MS data of ***(±) trans (9)***	82
81	**Figure S81.** ^{1}H NMR spectrum of ***(±) trans (11)***	83
82	**Figure S82.** LC-MS data of ***(±) trans (11)***	84

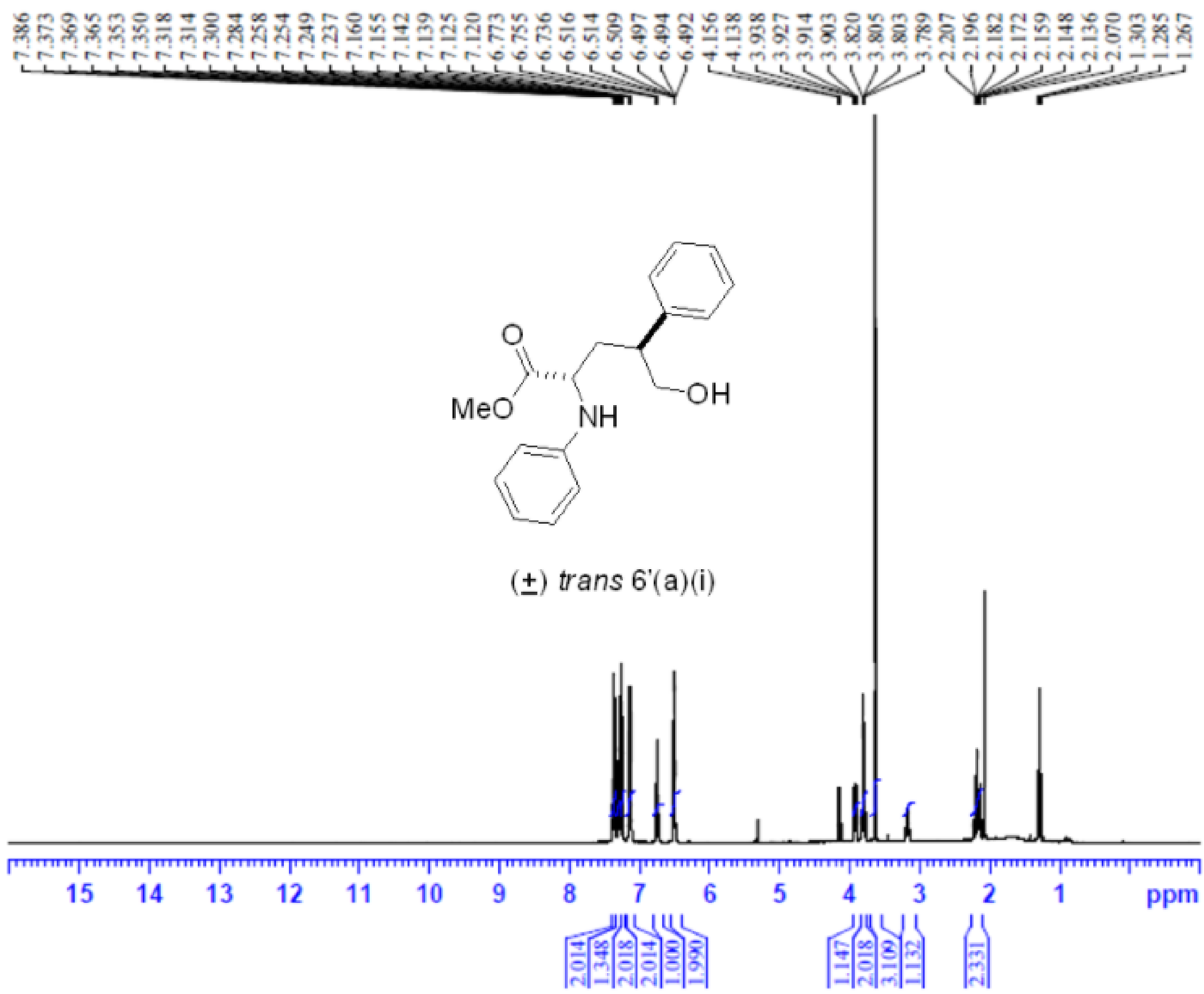

Figure S1. ^{1}H NMR spectrum of *(±) trans* 6’(a)(i)

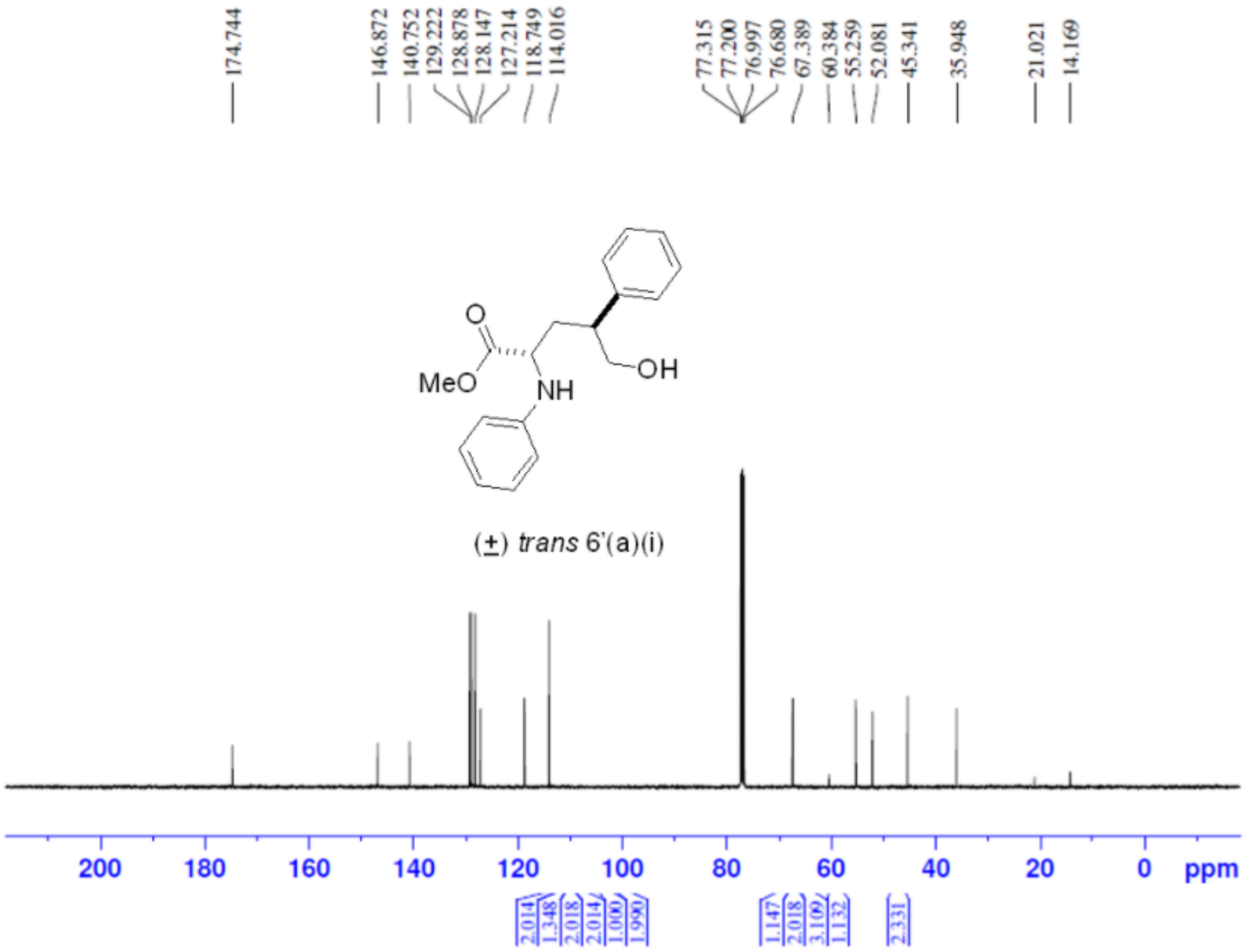

Figure S2. ^{13}C NMR spectrum of *(±) trans* 6'(a)(i)

v

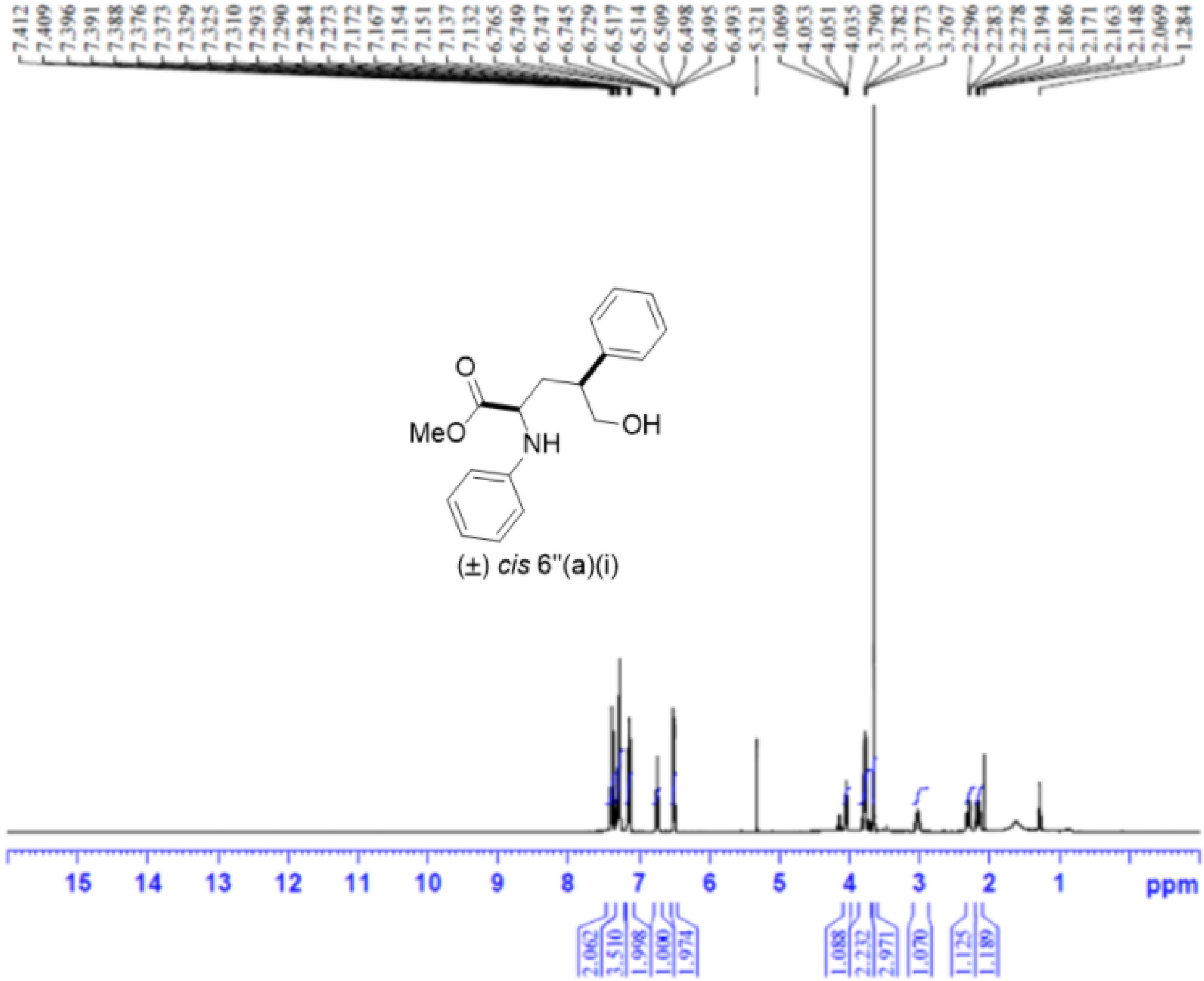

Figure S3. ^{1}H NMR spectrum of *(±) cis* 6''(a)(i)

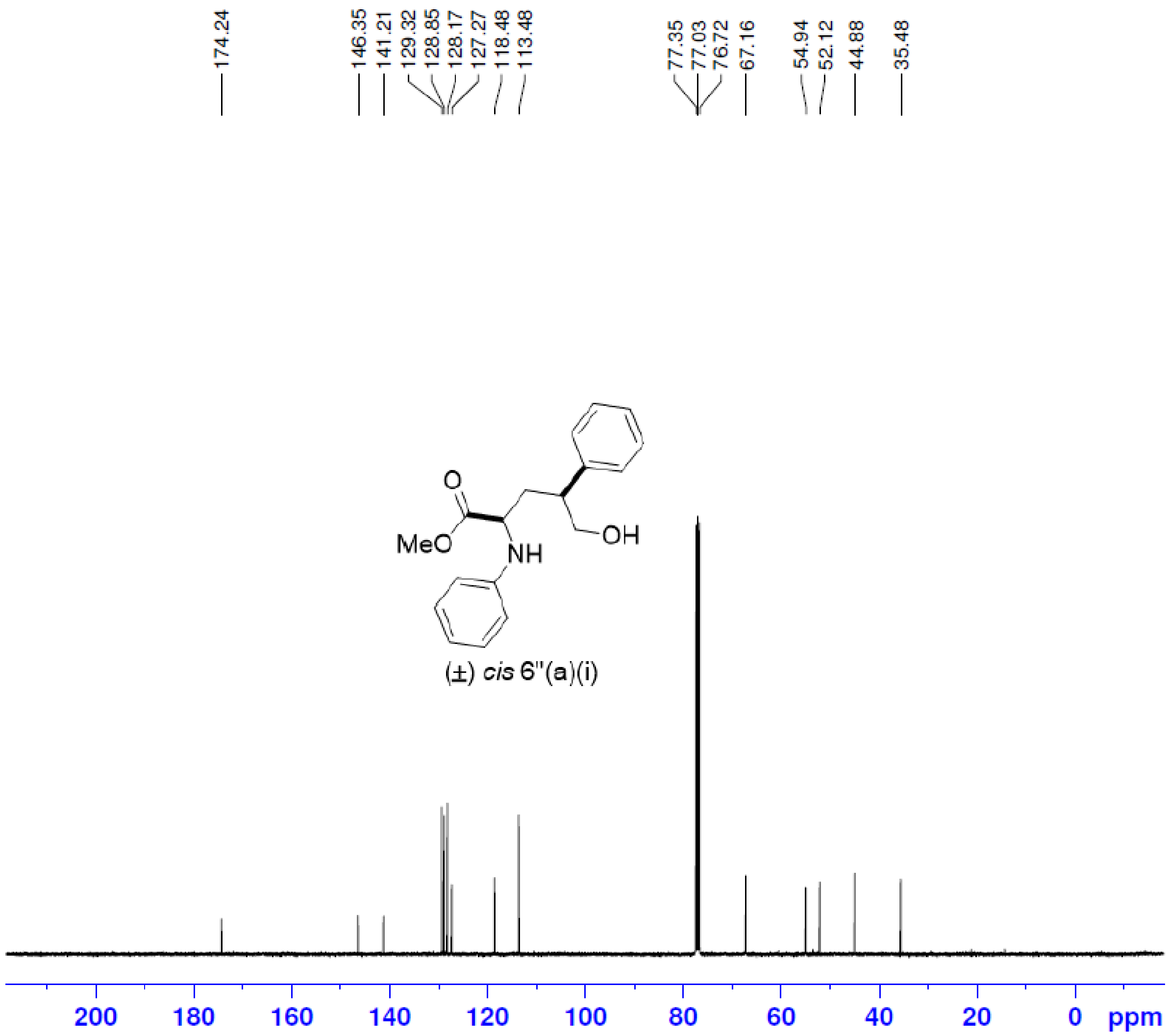

Figure S4. ^{13}C NMR spectrum of *(±) cis* 6''(a)(i)

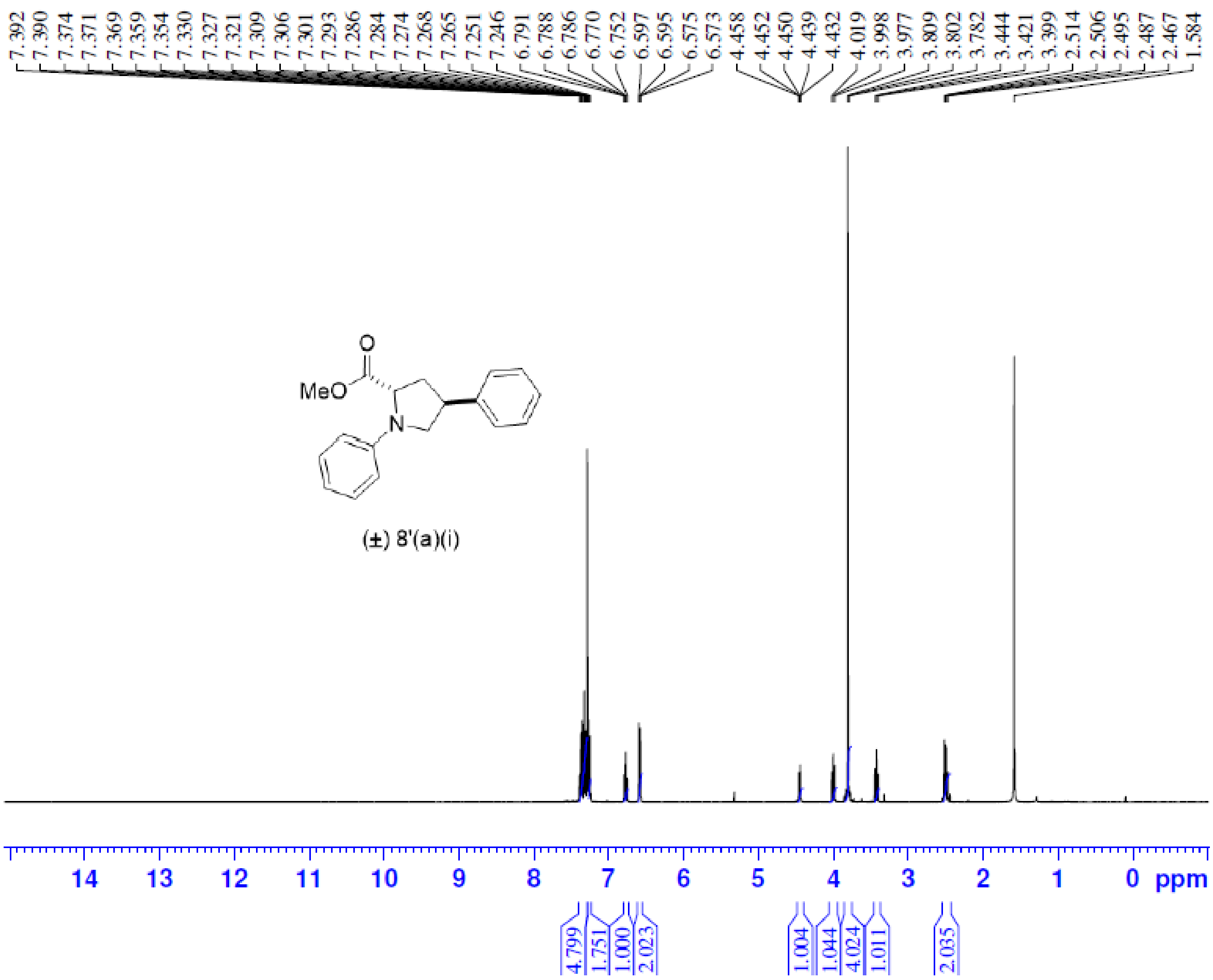

Figure S5. ^{1}H NMR spectrum of *(±) trans* 8'(a)(i)

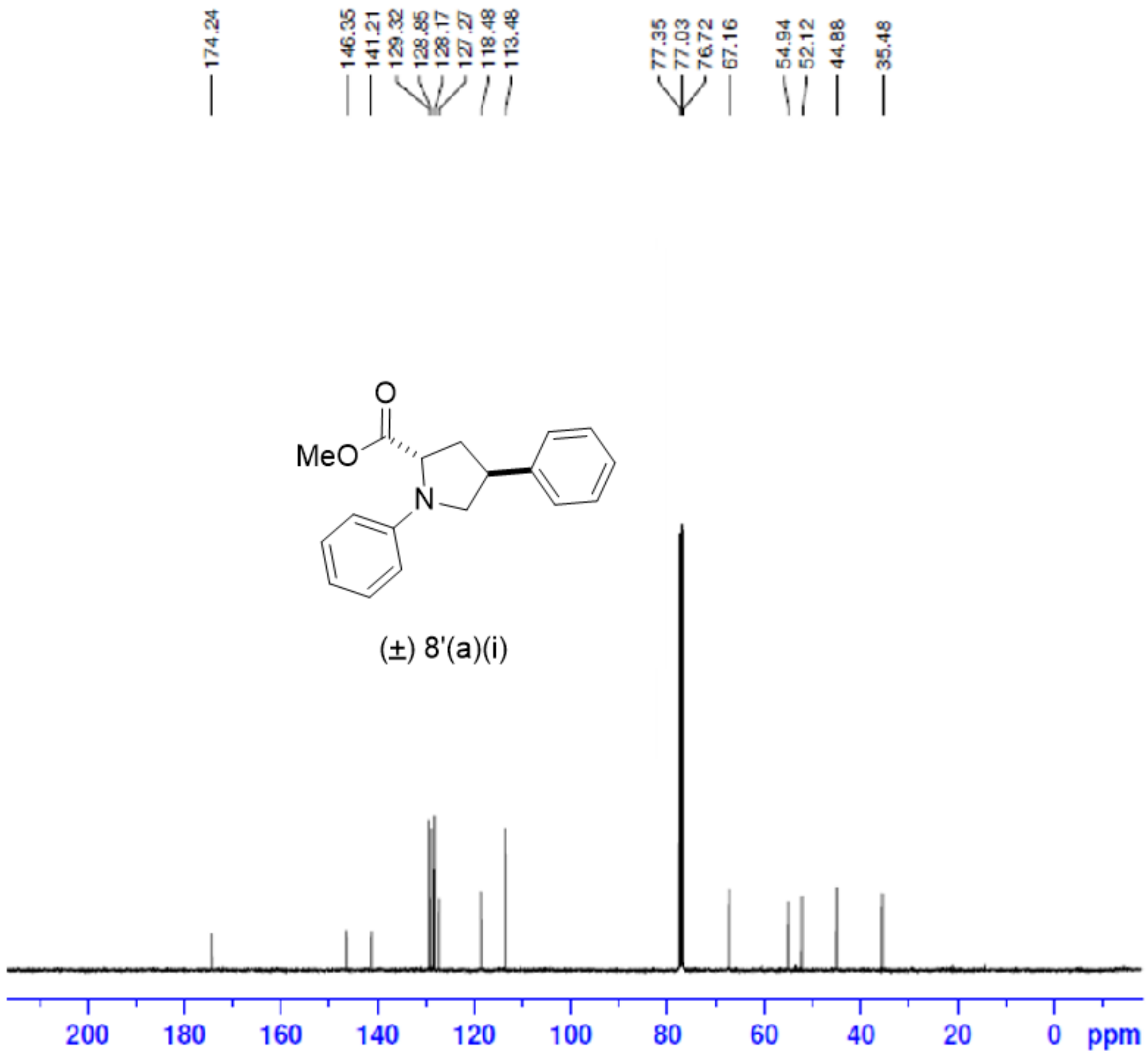

Figure S6. ^{13}C NMR spectrum of *(±) trans* 8'(a)(i)

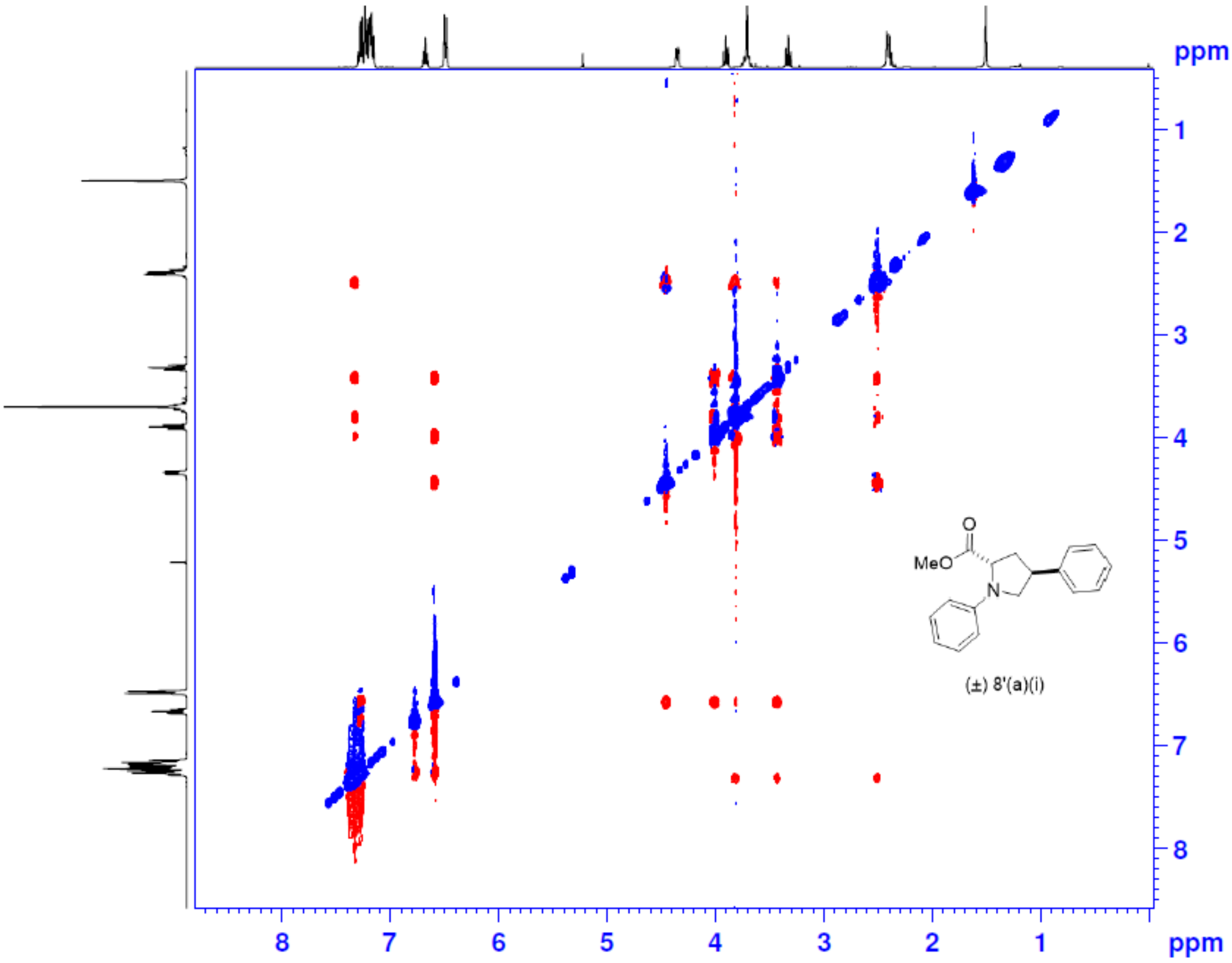

Figure S7. NOESY spectrum of *(±) trans* 8’(a)(i)

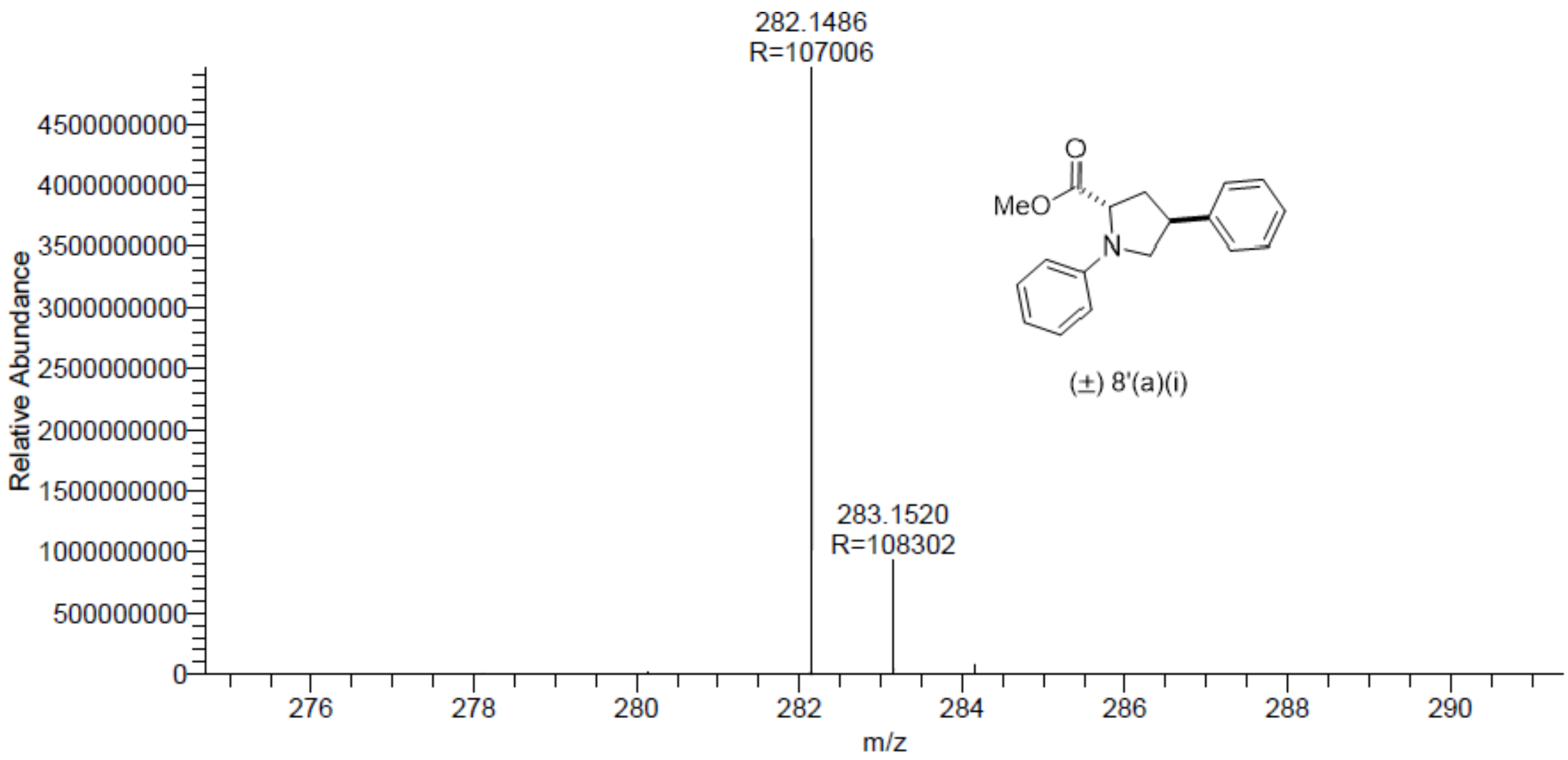

Figure S8. HRMS data of *(±) trans* 8'(a)(i)

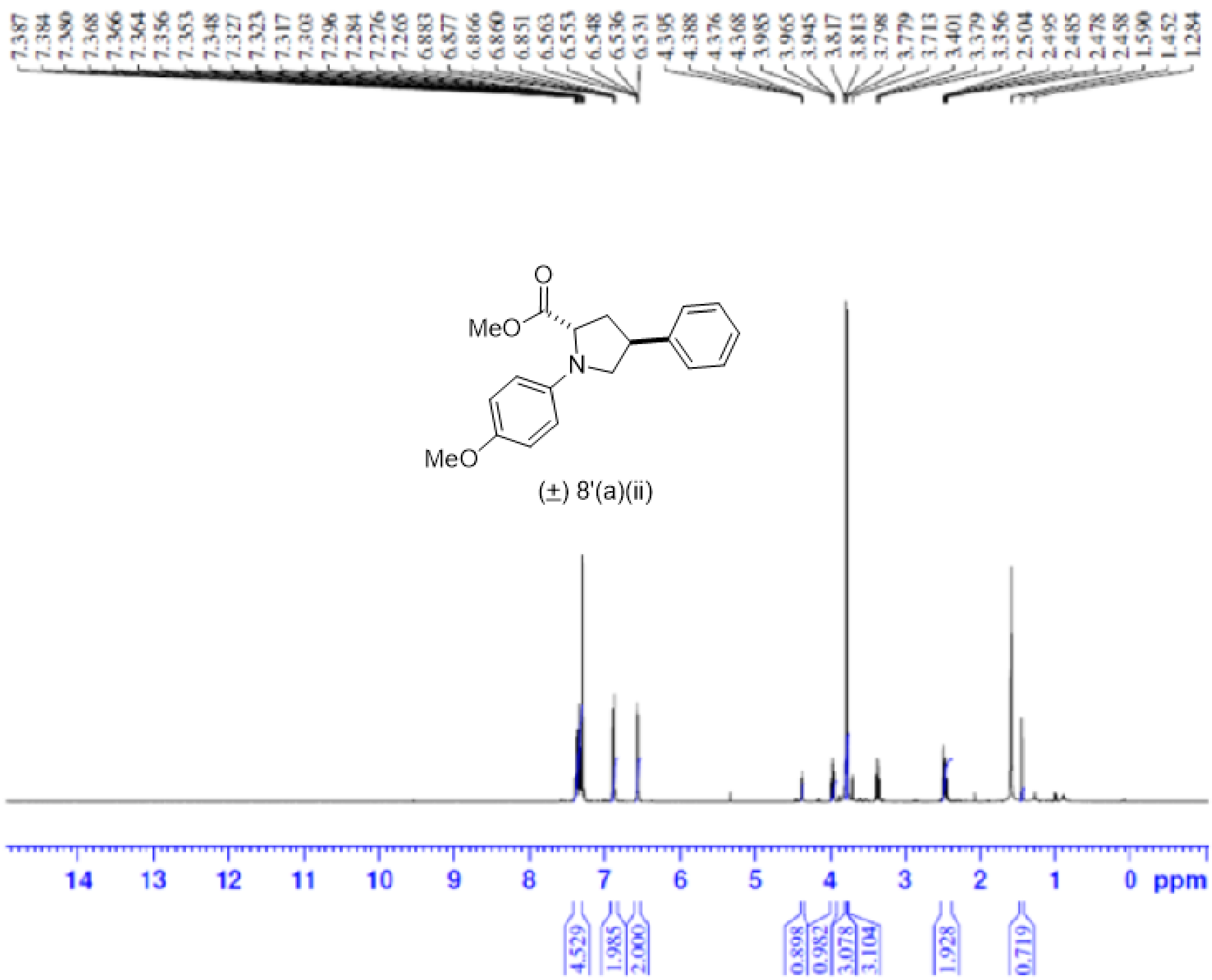

Figure S9. ^{1}H NMR spectrum of *(±) trans* 8'(a)(ii)

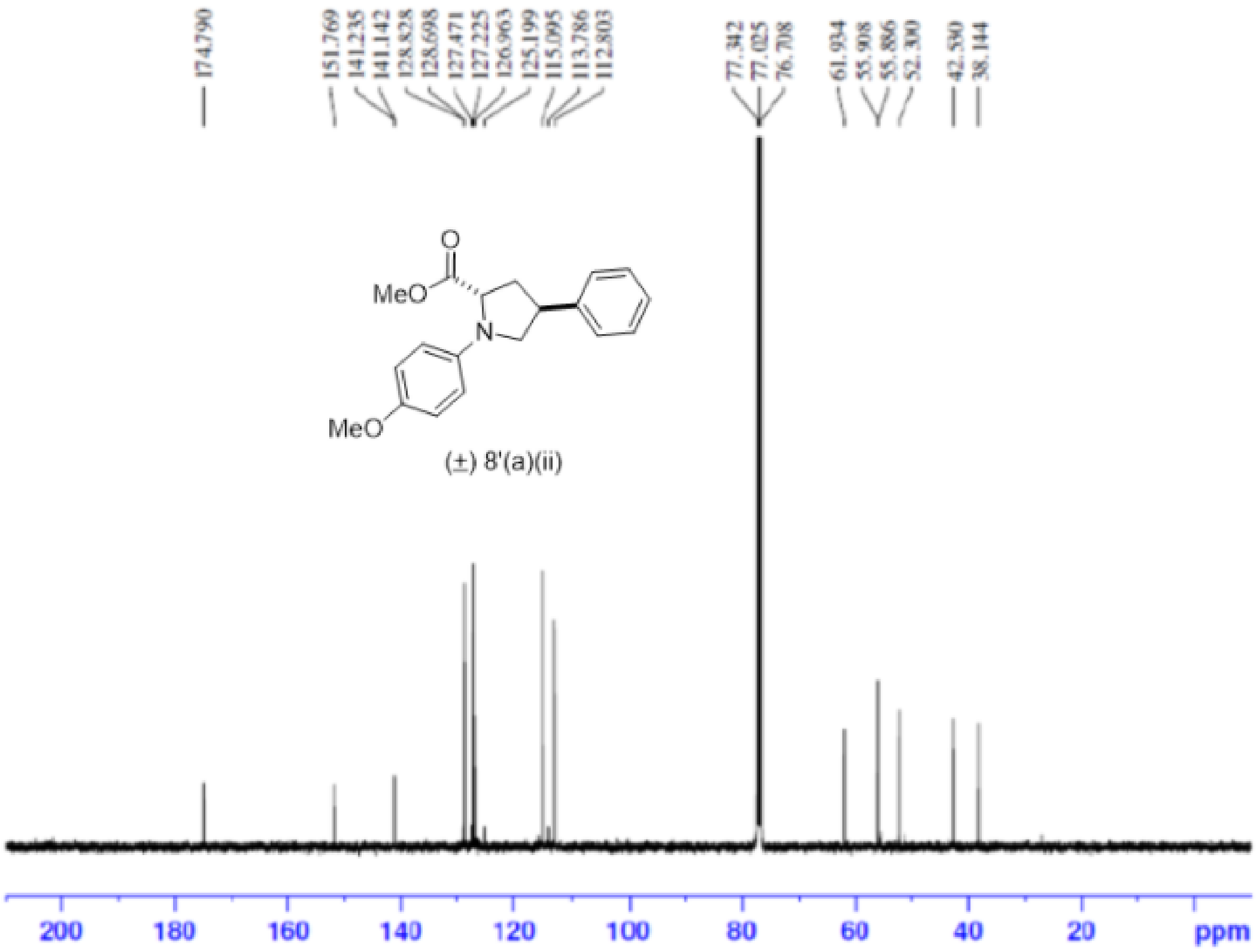

Figure S10. ^{13}C NMR spectrum of *(±) trans* 8'(a)(ii)

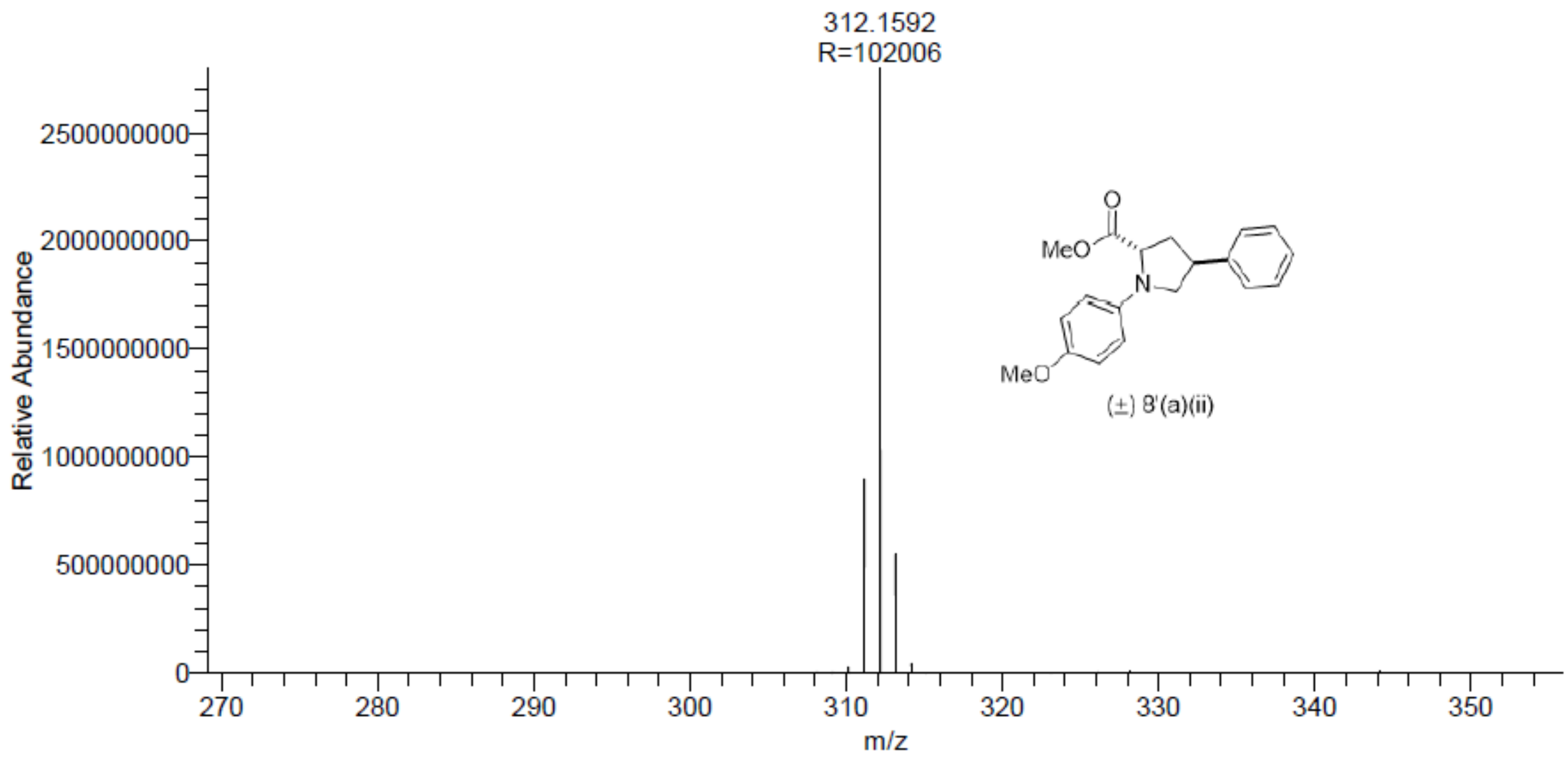

Figure S11. HRMS data of *(±) trans* 8’(a)(ii)

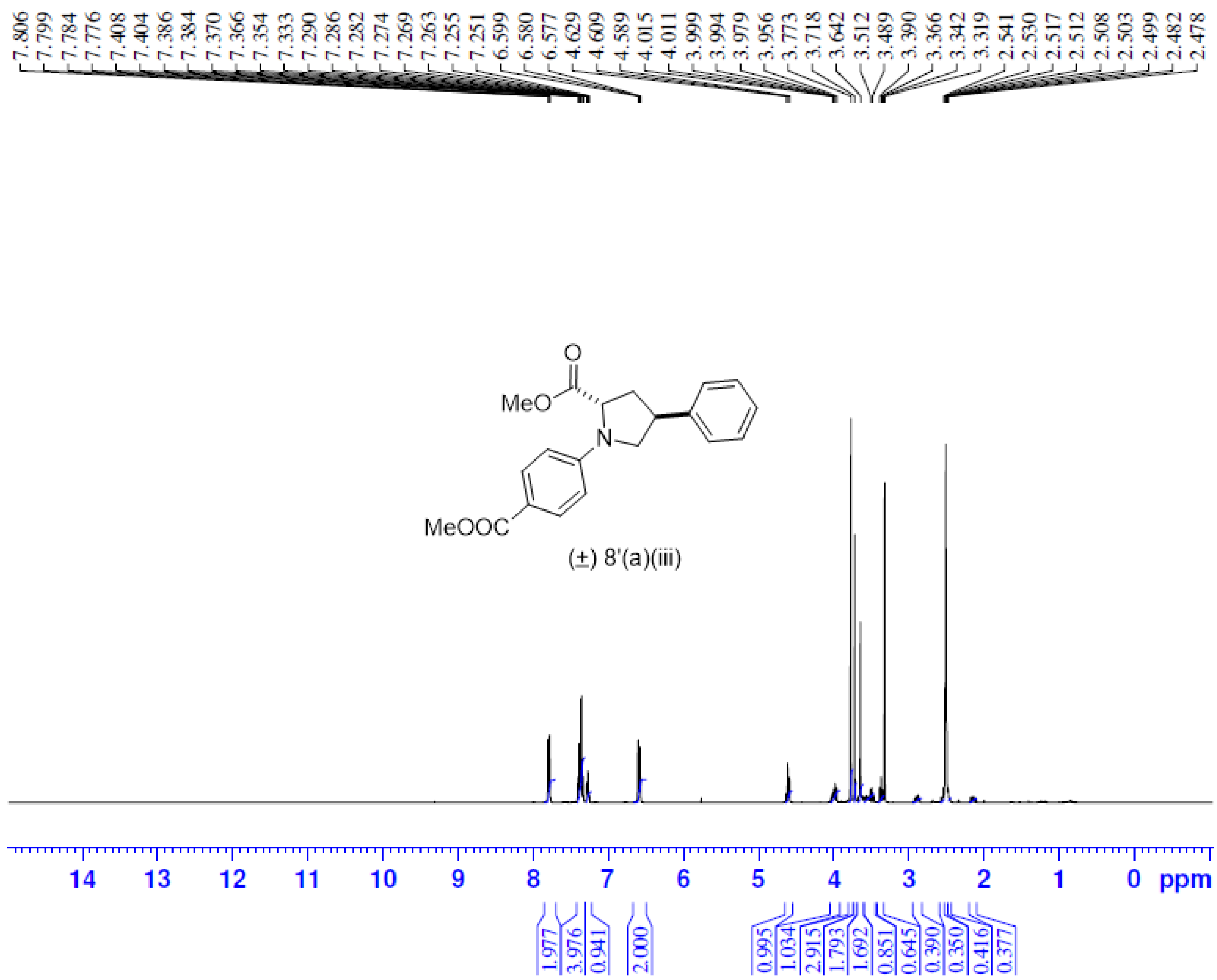

Figure S12. ^{1}H NMR spectrum of *(±) trans* 8'(a)(iii)

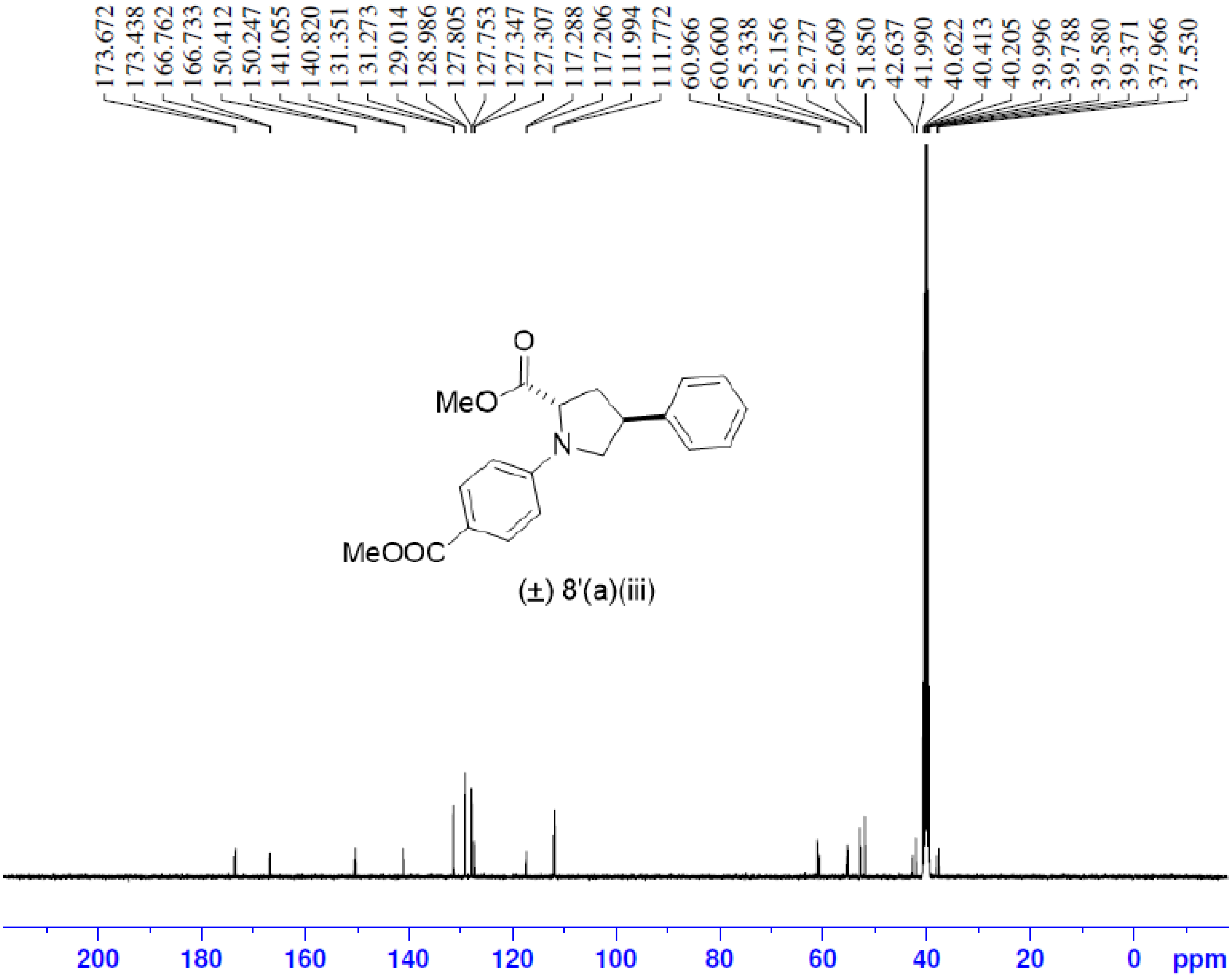

Figure S13. ^{13}C NMR spectrum of *(±) trans* 8'(a)(iii)

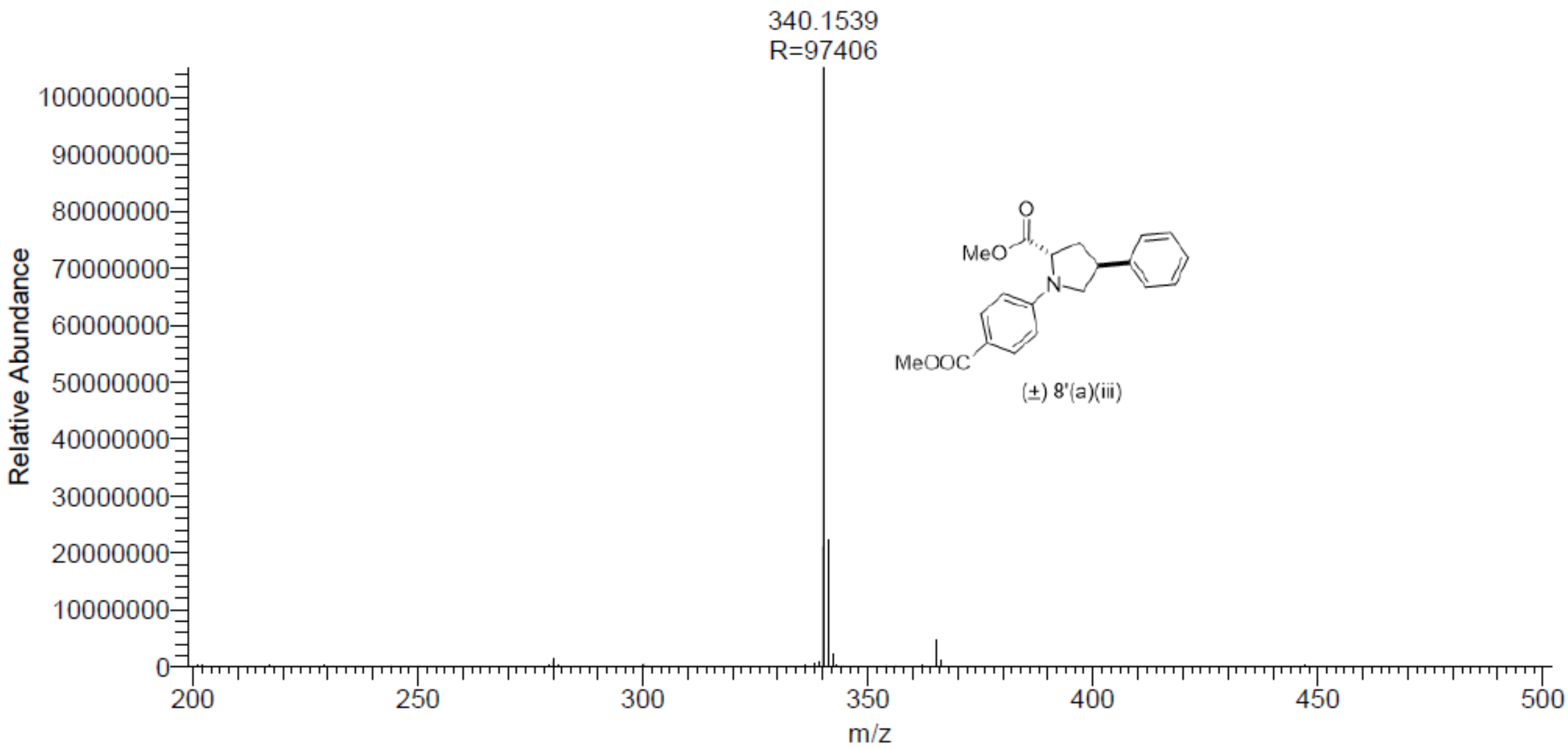

Figure S14. HRMS data of *(±) trans* 8’(a)(iii)

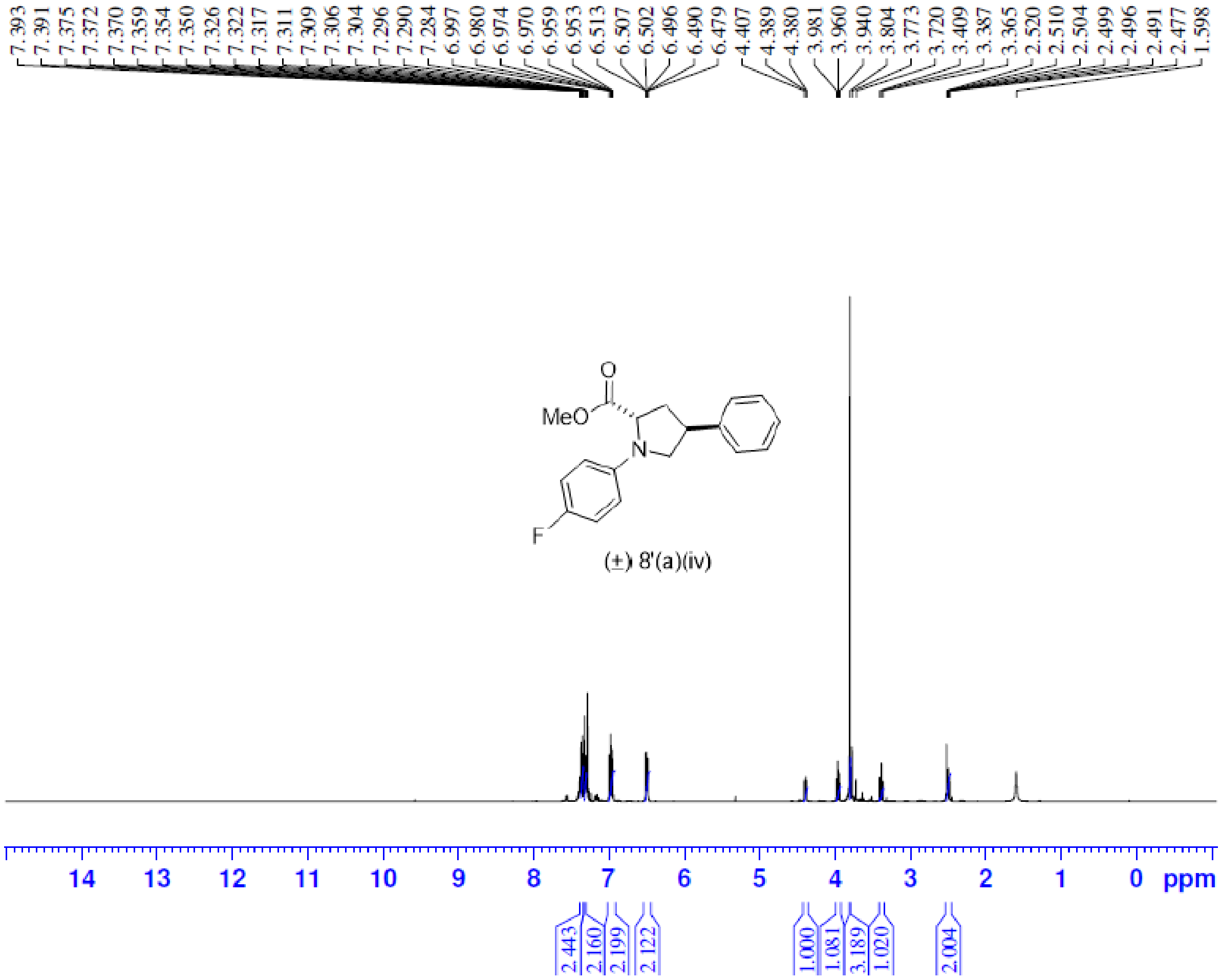

Figure S15. ^{1}H NMR spectrum of *(±) trans* 8'(a)(iv)

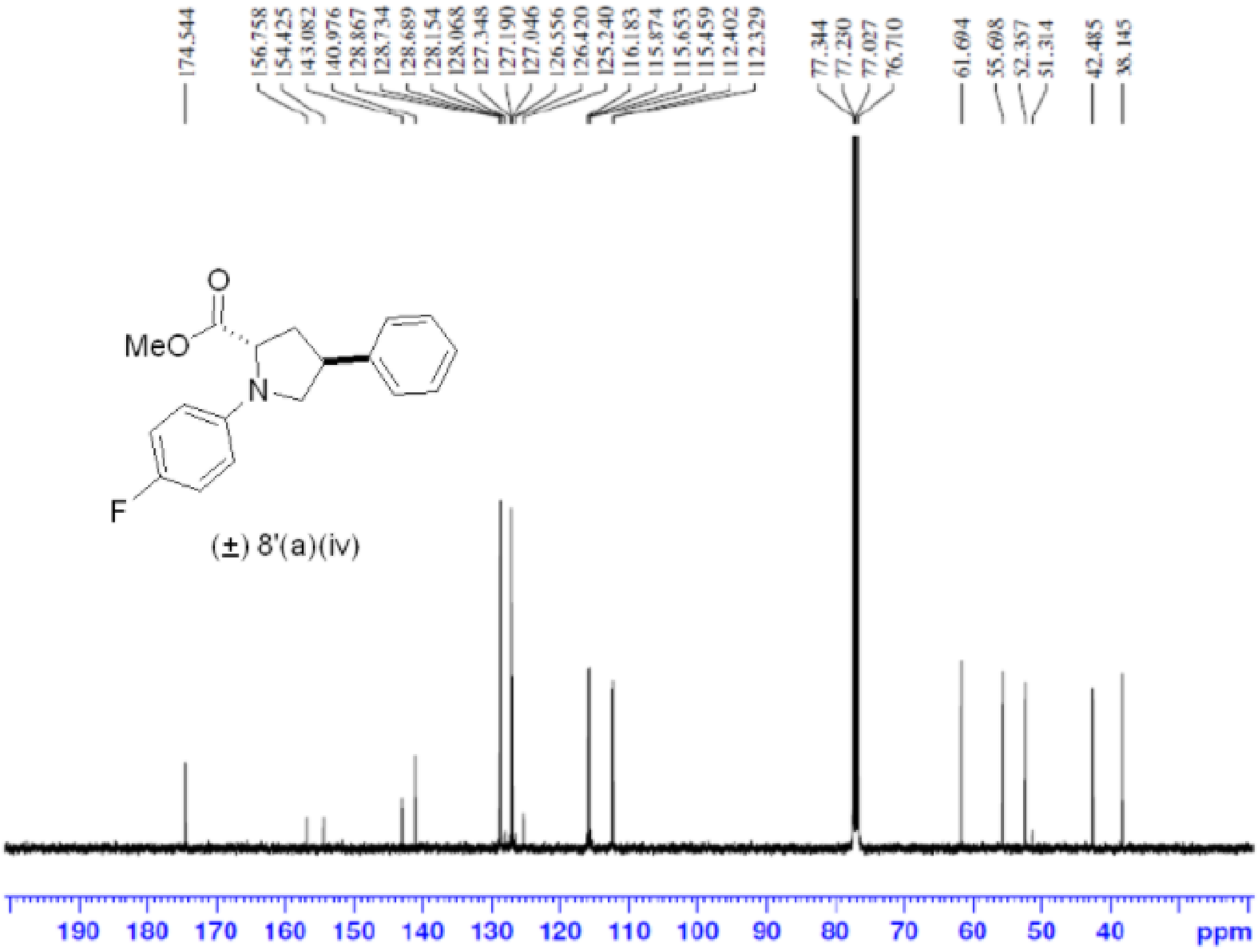

Figure S16. ^{13}C NMR spectrum of *(±) trans* 8’(a)(iv)

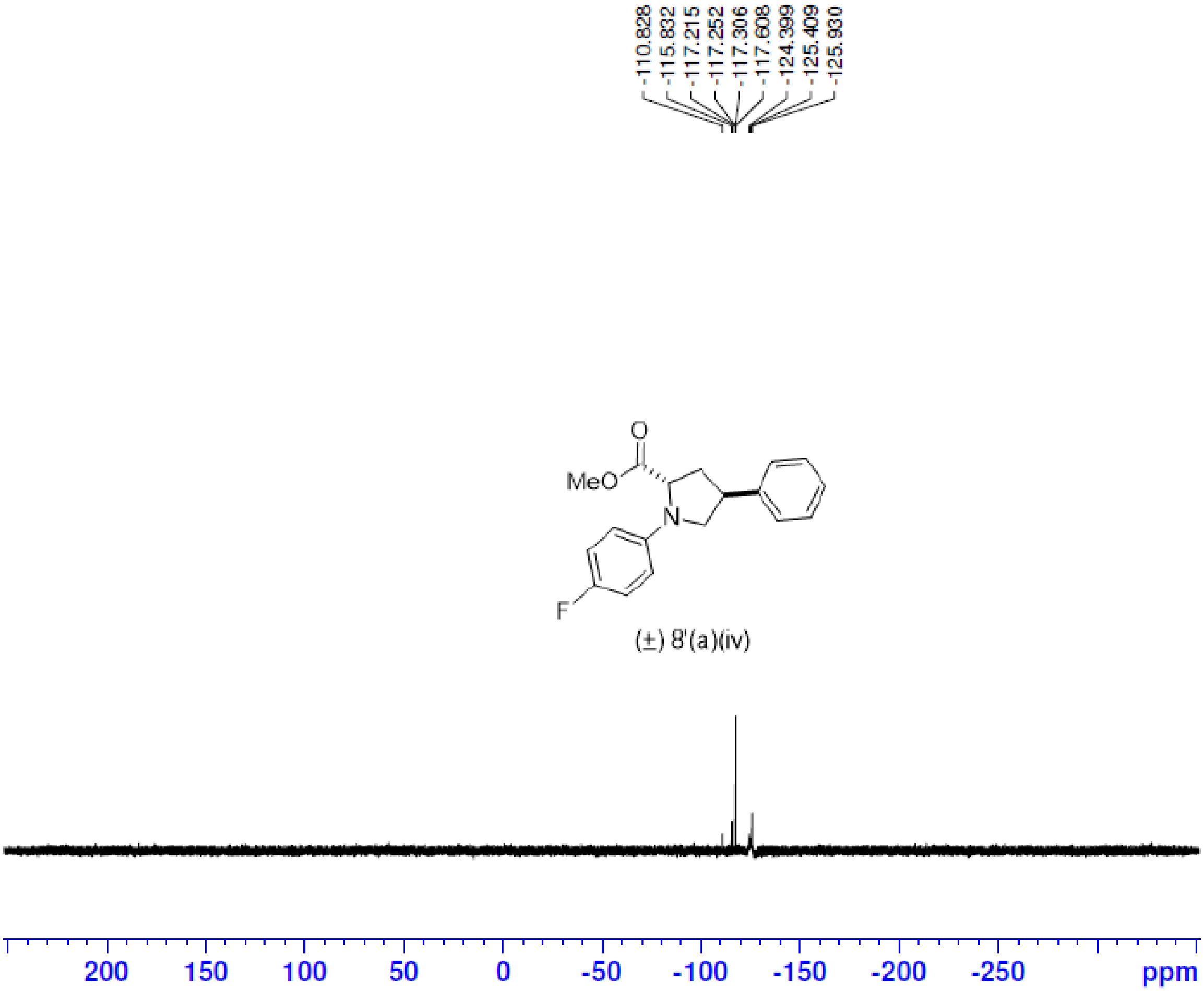

Figure S17. ^{19}F NMR spectrum of *(±) trans* 8’(a)(iv)

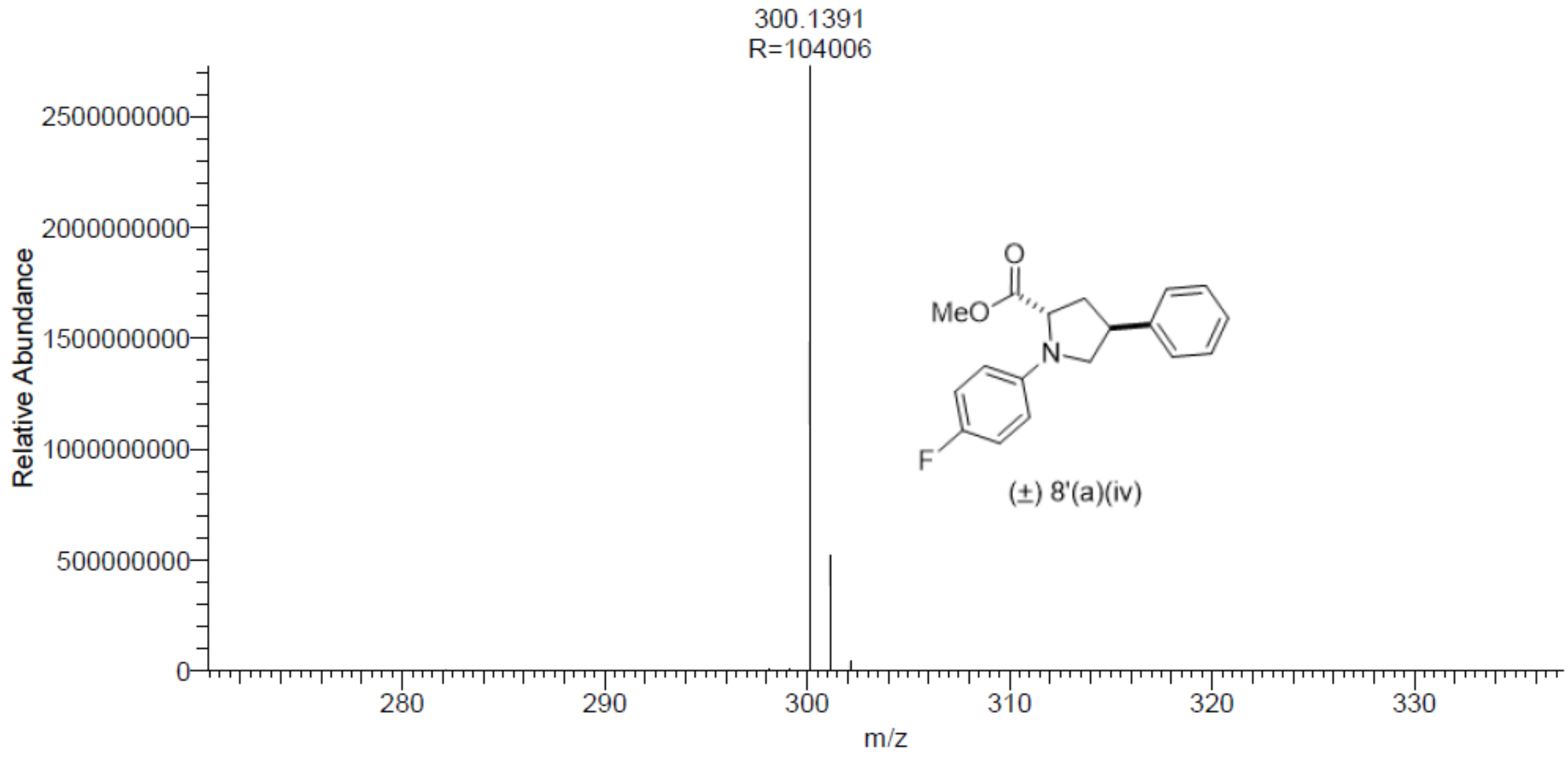

Figure S18. HRMS data of *(±) trans* 8’(a)(iv)

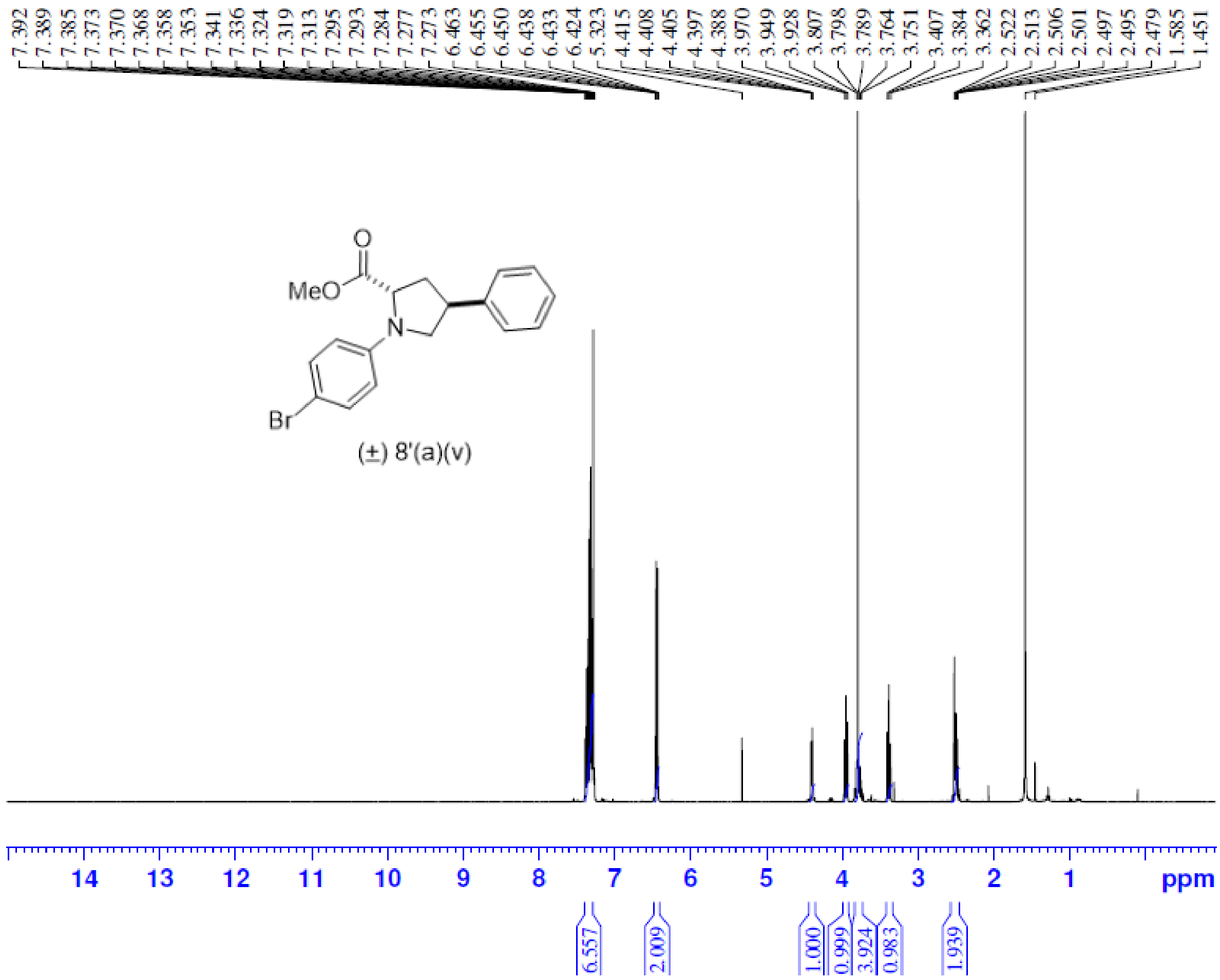

Figure S19. ¹H NMR spectrum of *(±) trans* 8'(a)(v)

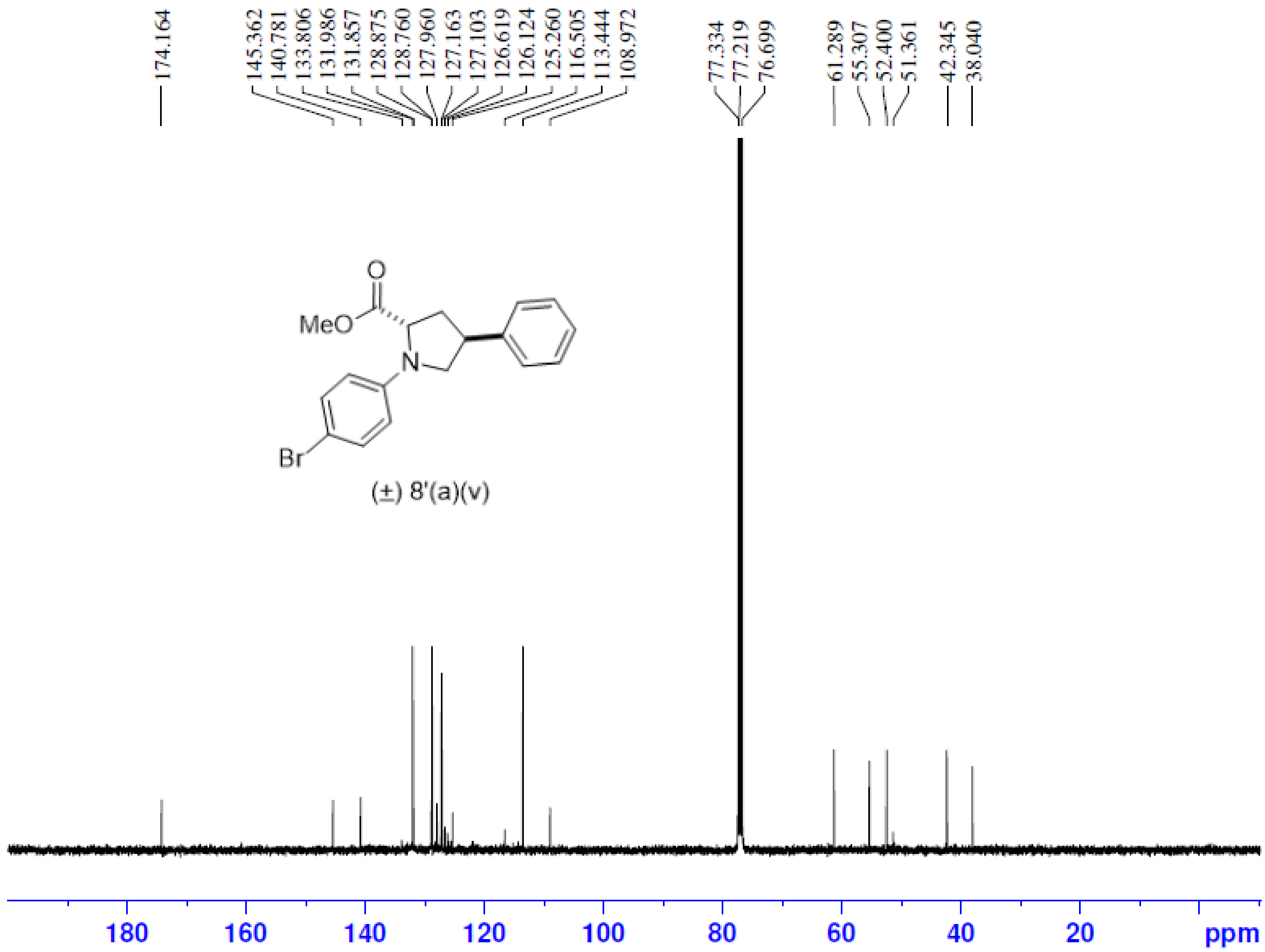

Figure S20. ^{13}C NMR spectrum of *(±) trans* 8'(a)(v)

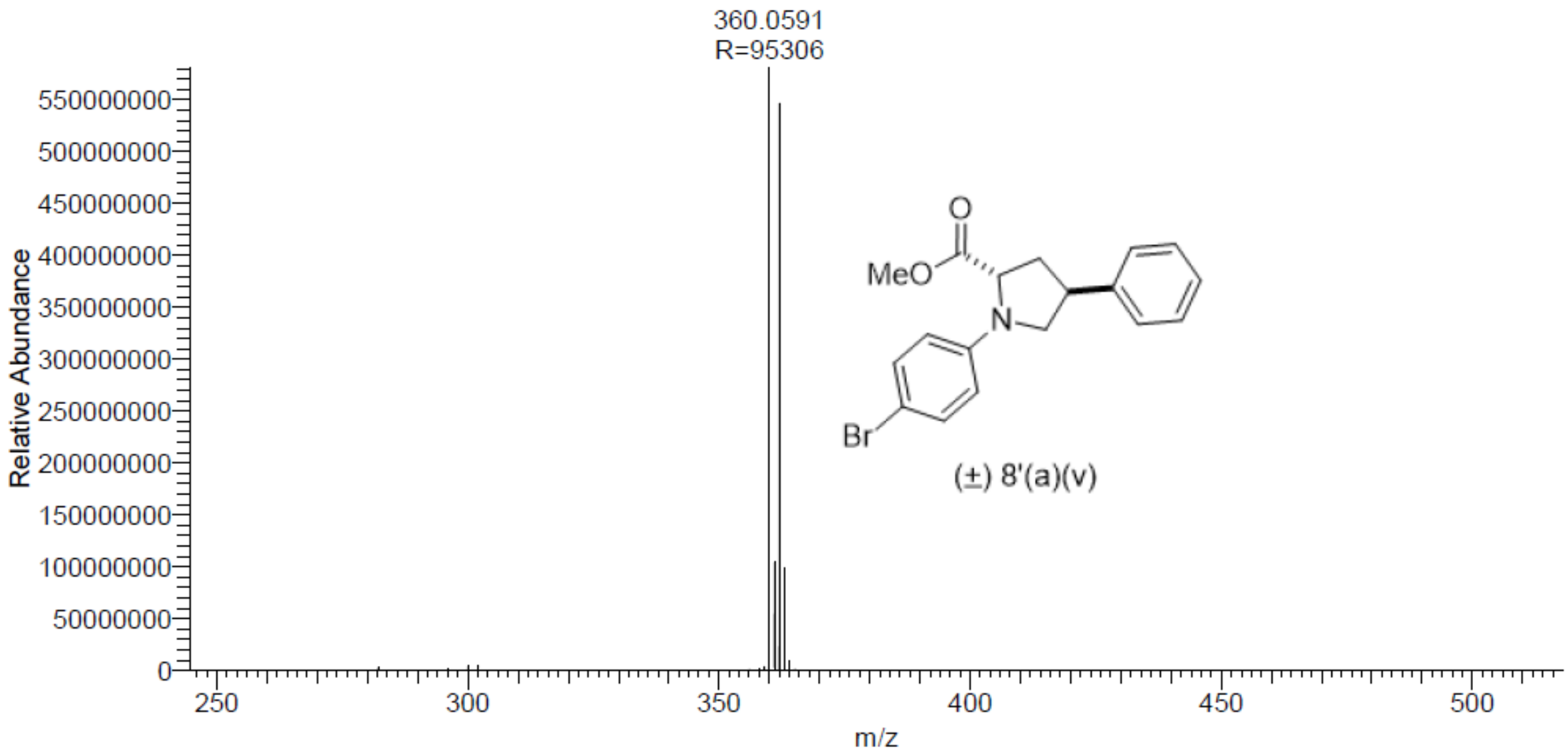

Figure S21. HRMS data of *(±) trans* 8’(a)(v)

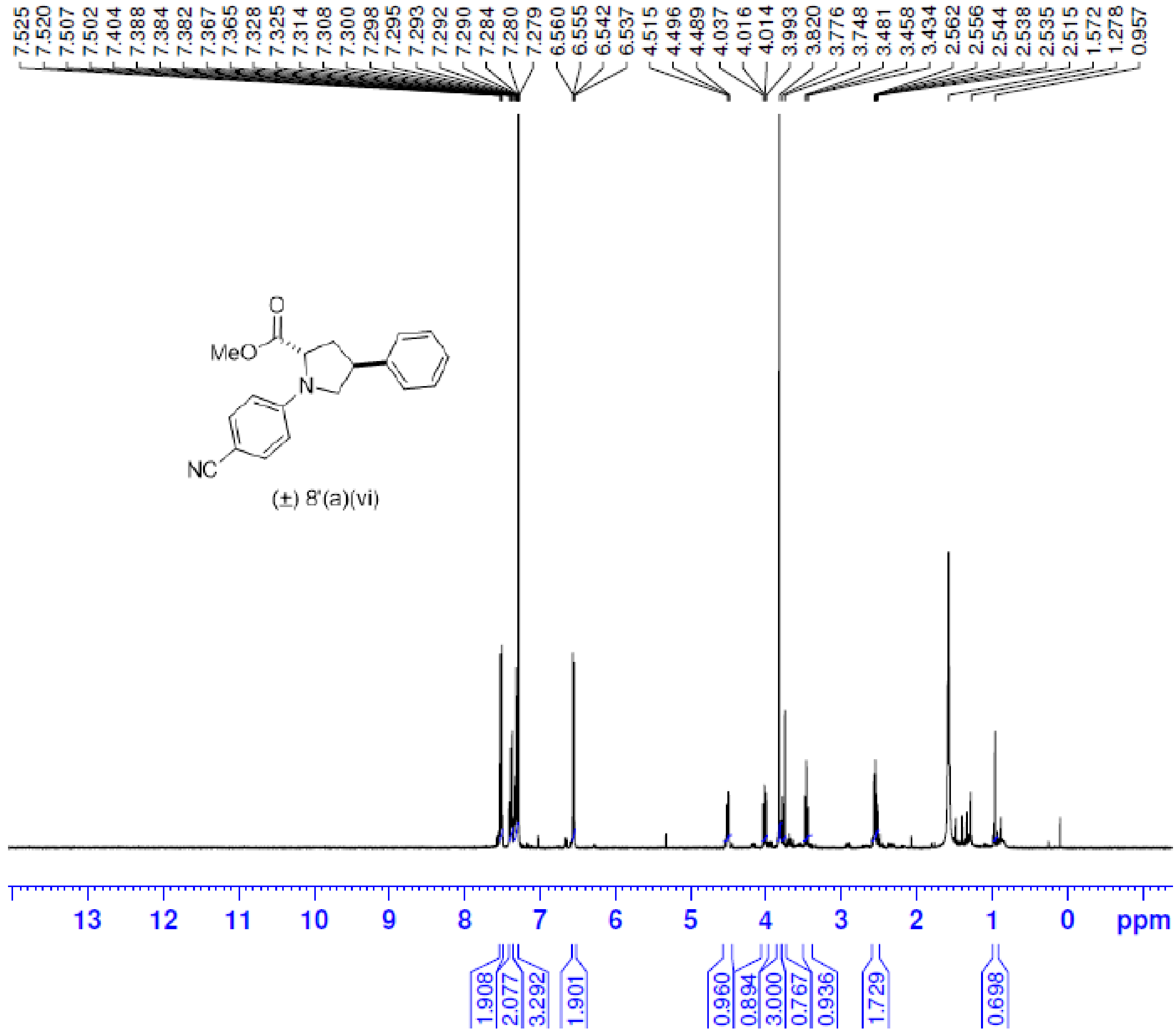

Figure S22. ^{1}H NMR spectrum of *(±) trans* 8'(a)(vi)

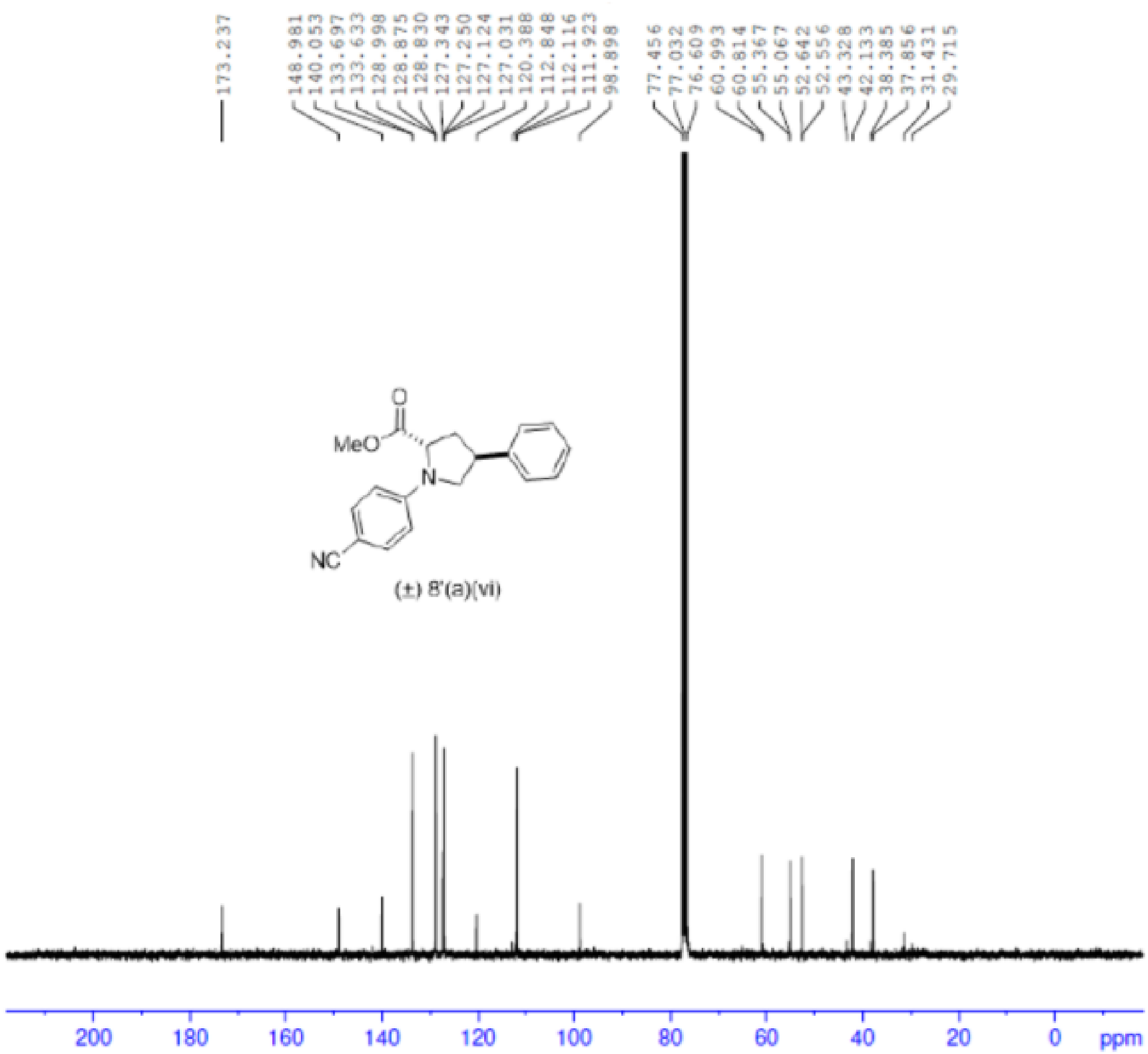

Figure S23. ^{13}C NMR spectrum of *(±) trans* 8'(a)(vi)

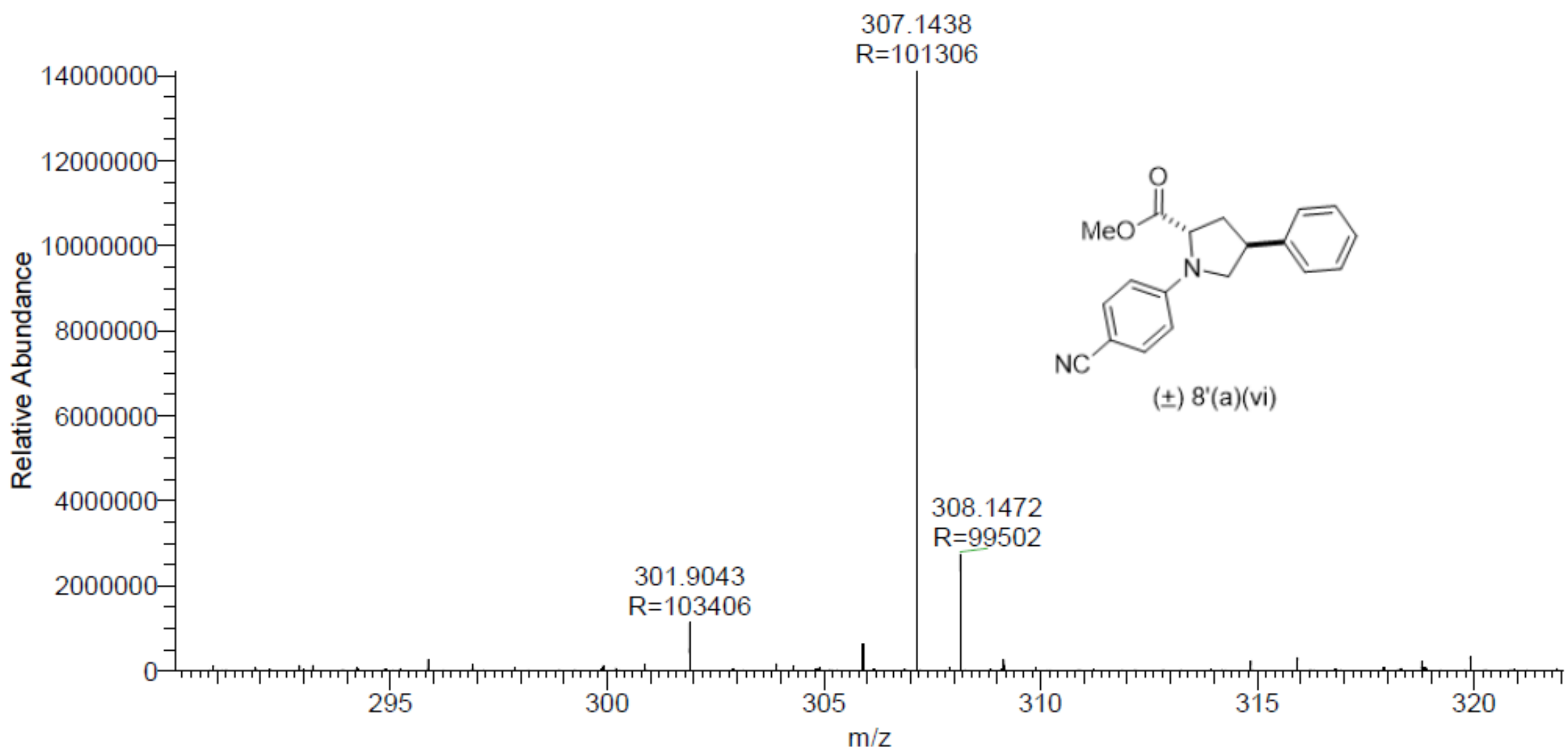

Figure S24. HRMS data of *(±) trans* 8’(a)(vi)

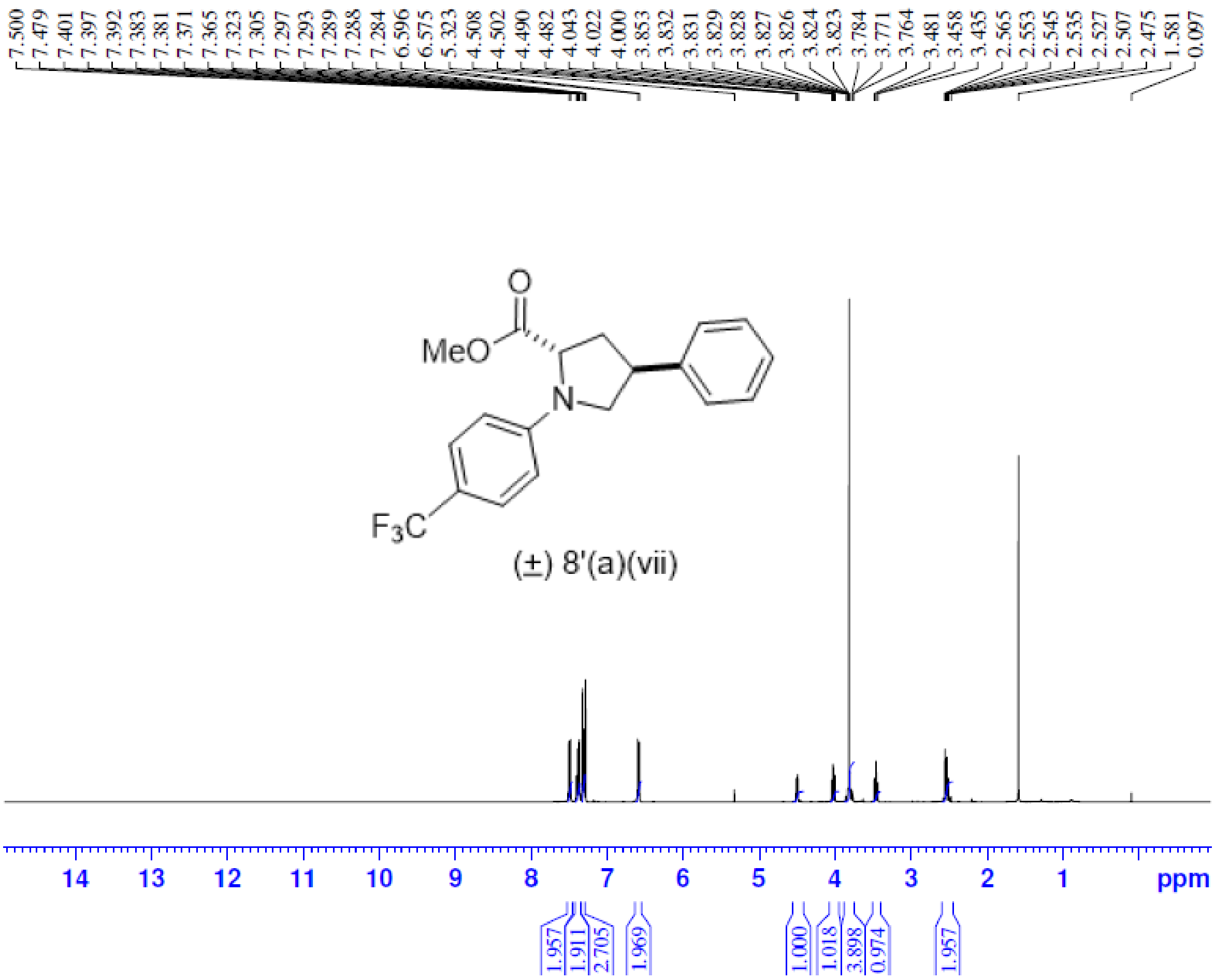

Figure S25. ^{1}H NMR spectrum of *(±) trans* 8'(a)(vii)

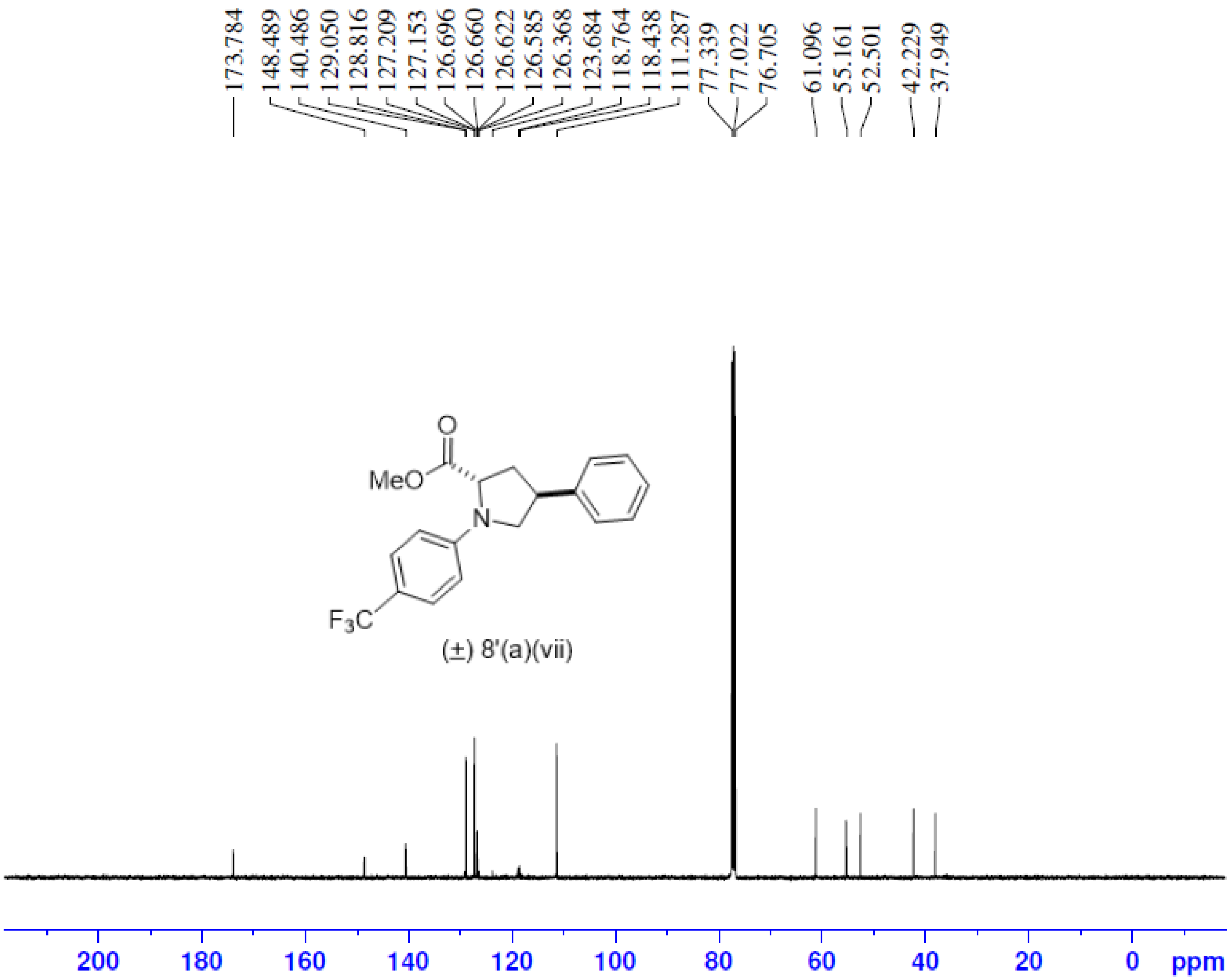

Figure S26. ^{13}C NMR spectrum of *(±) trans* 8'(a)(vii)

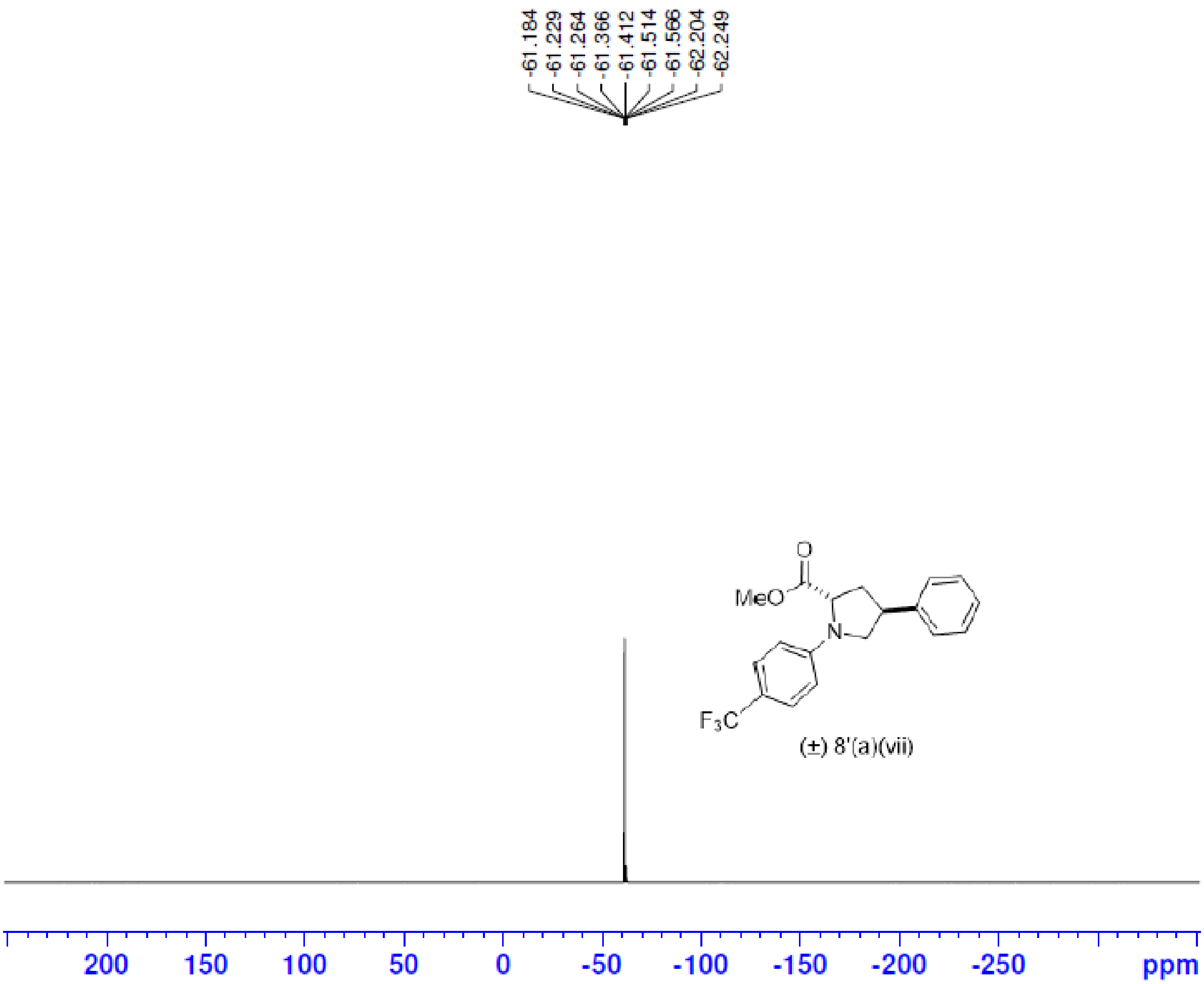

Figure S27. ^{19}F NMR spectrum of *(±) trans* 8'(a)(vii)

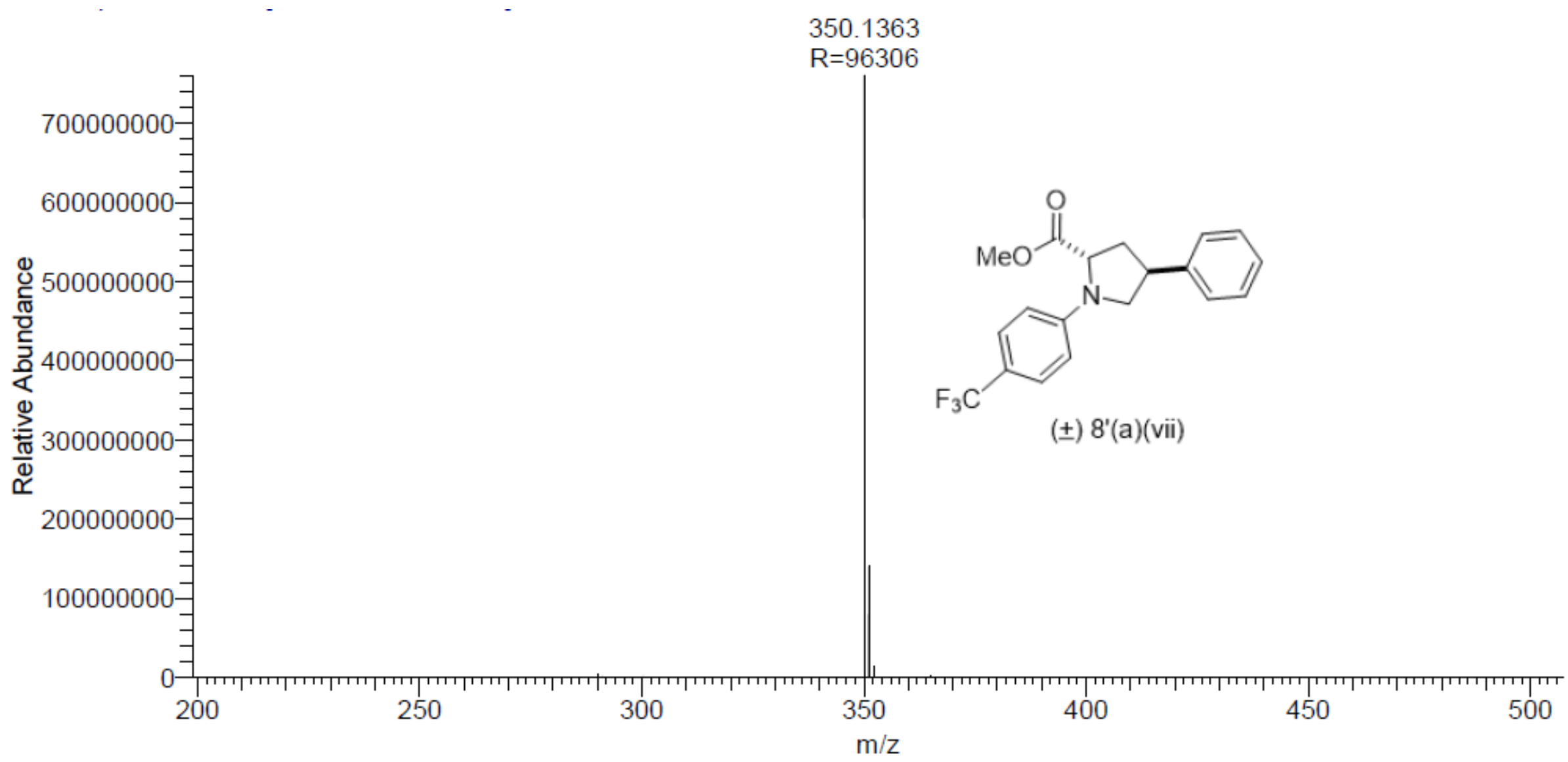

Figure S28. HRMS data of *(±) trans* 8'(a)(vii)

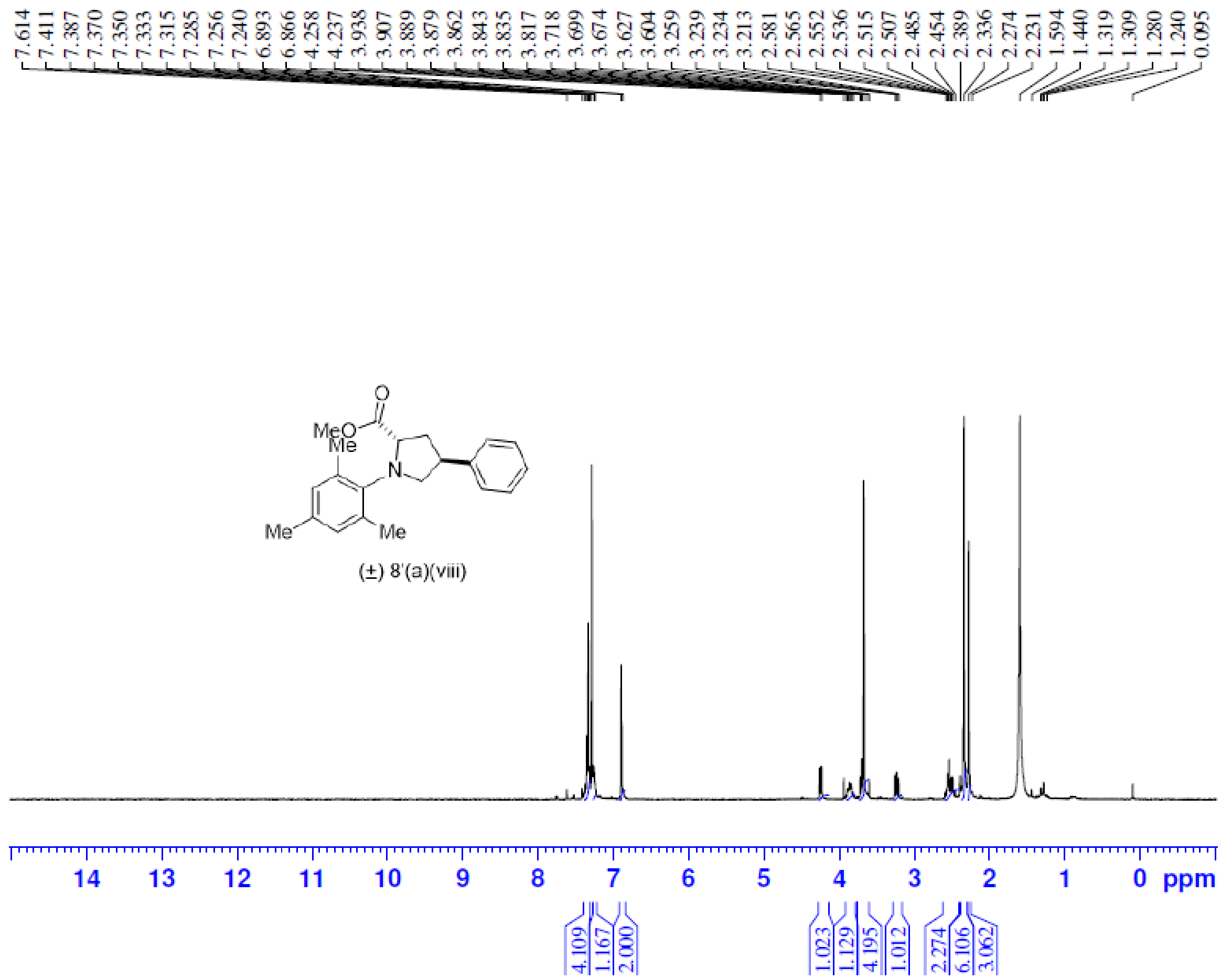

Figure S29. ^{1}H NMR spectrum of *(±) trans* 8'(a)(viii)

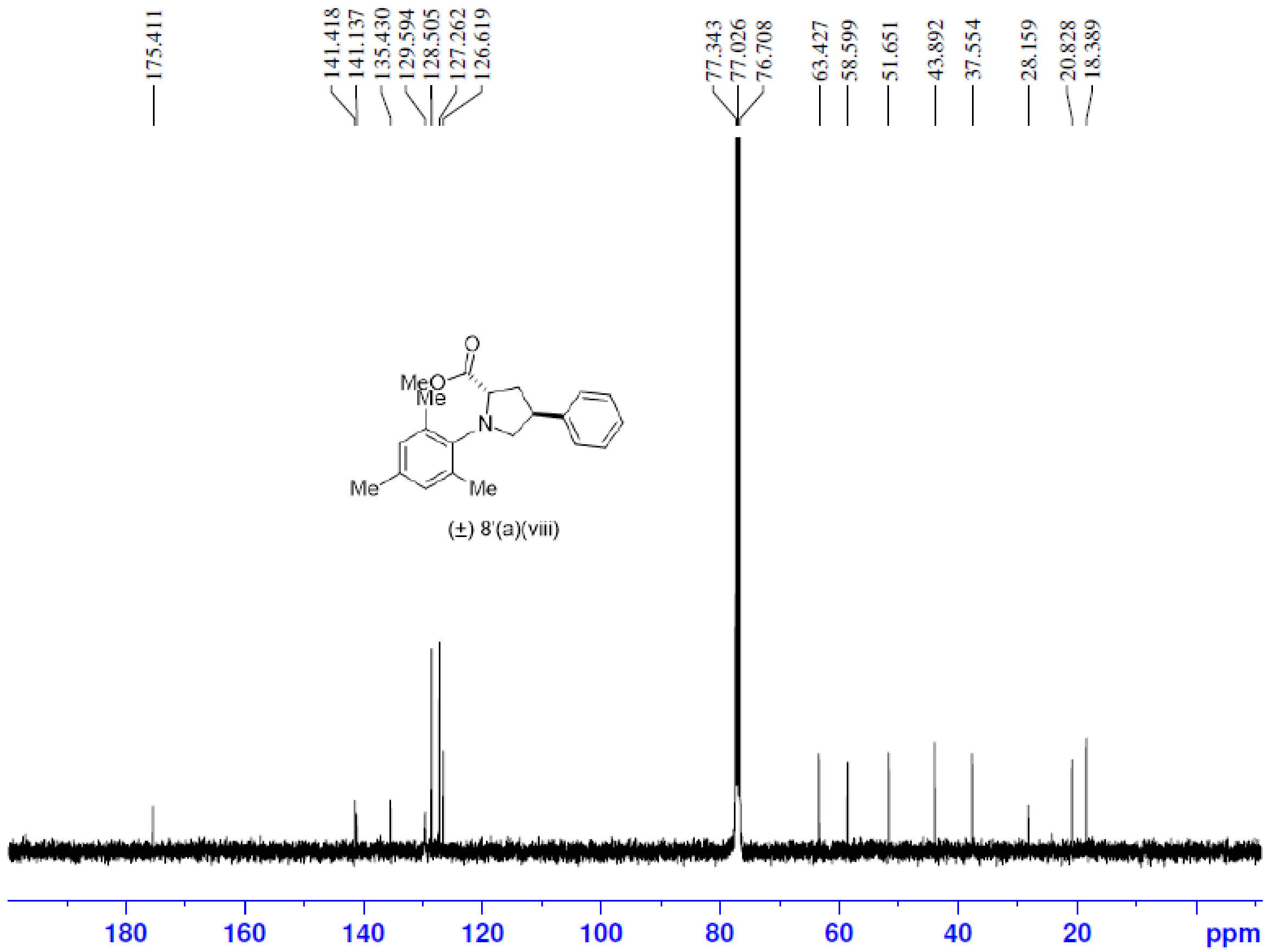

Figure S30. ^{13}C NMR spectrum of *(±) trans* 8’(a)(viii)

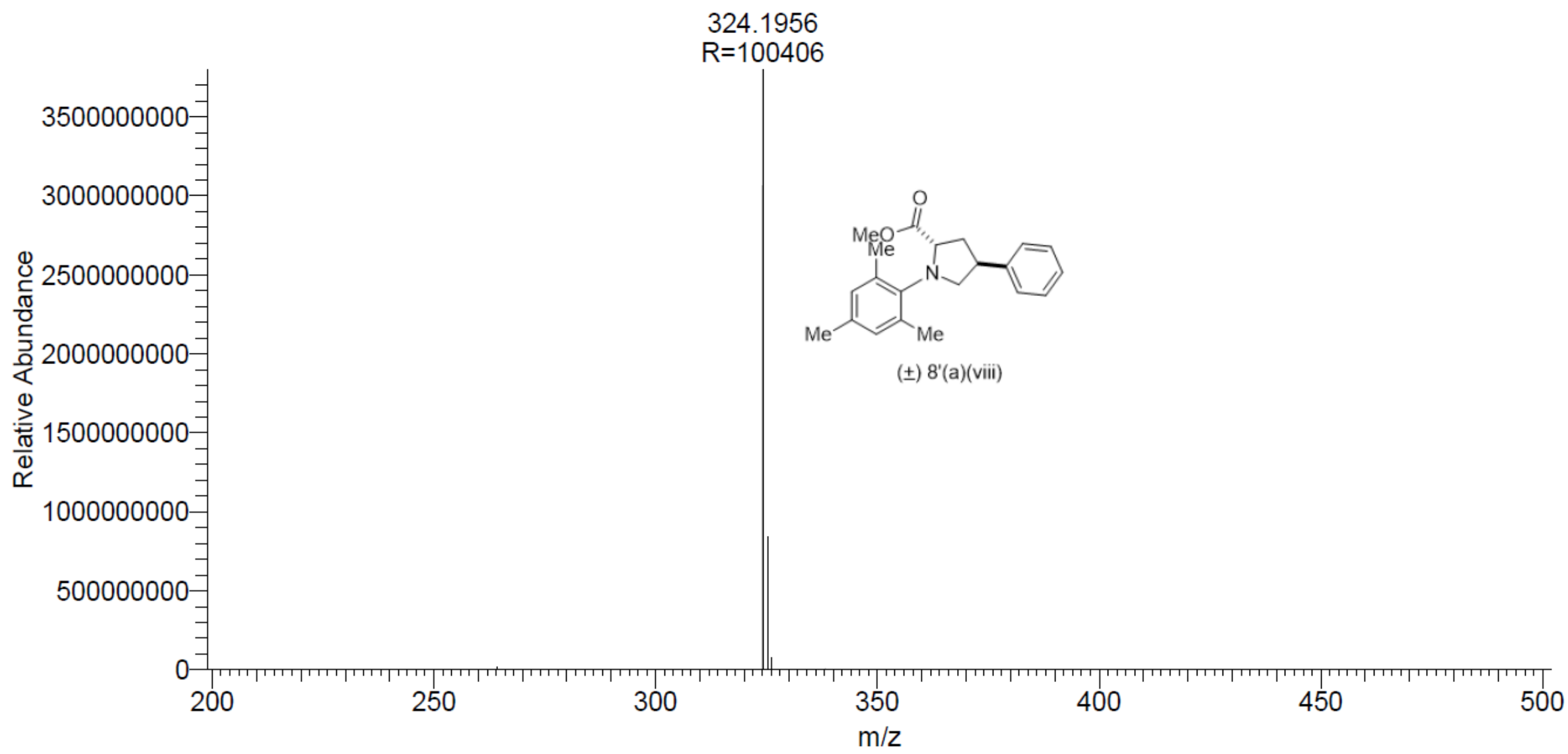

Figure S31. HRMS data of *(±) trans* 8'(a)(viii)

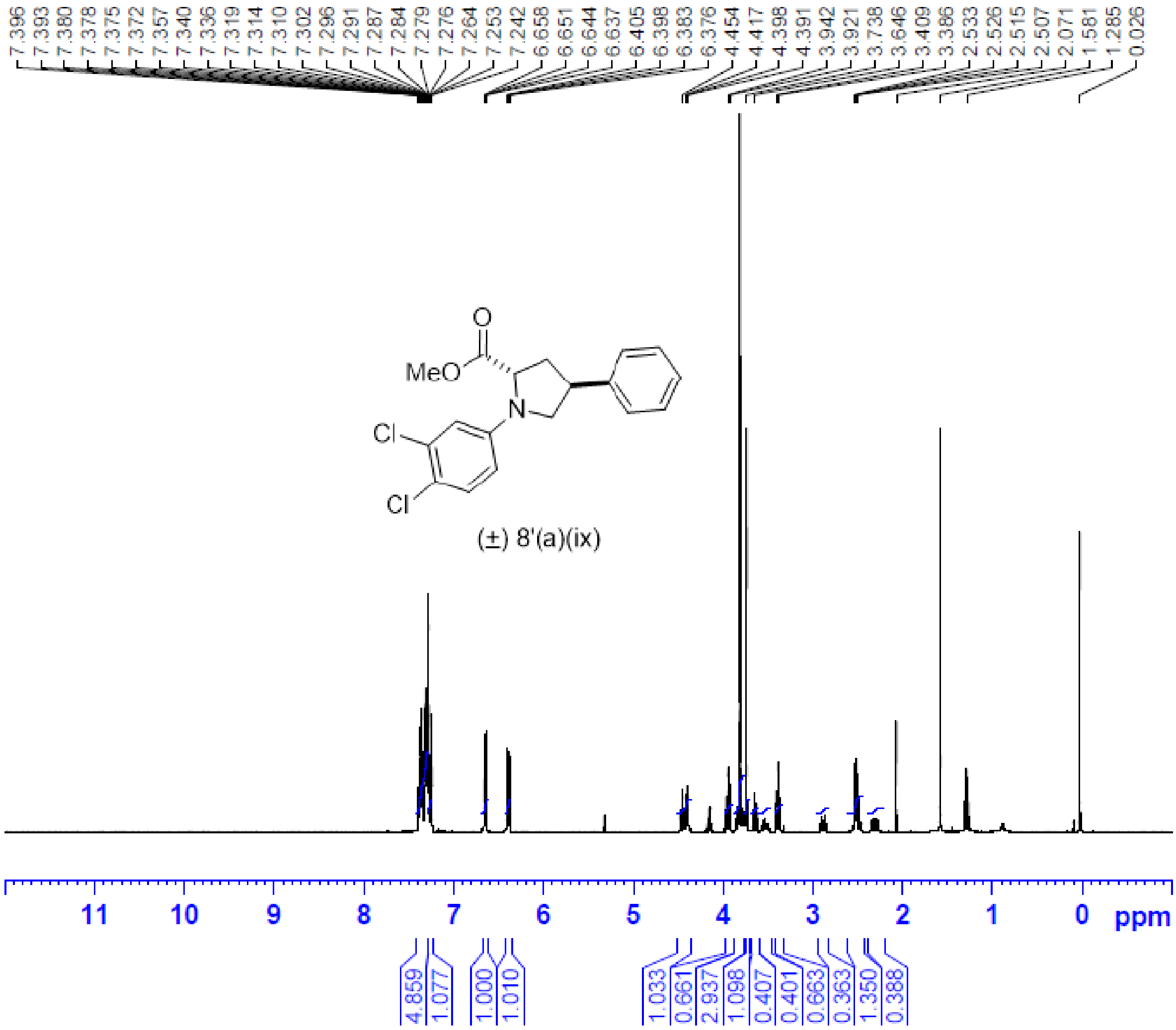

Figure S32. ^{1}H NMR spectrum of *(±) trans* 8'(a)(ix)

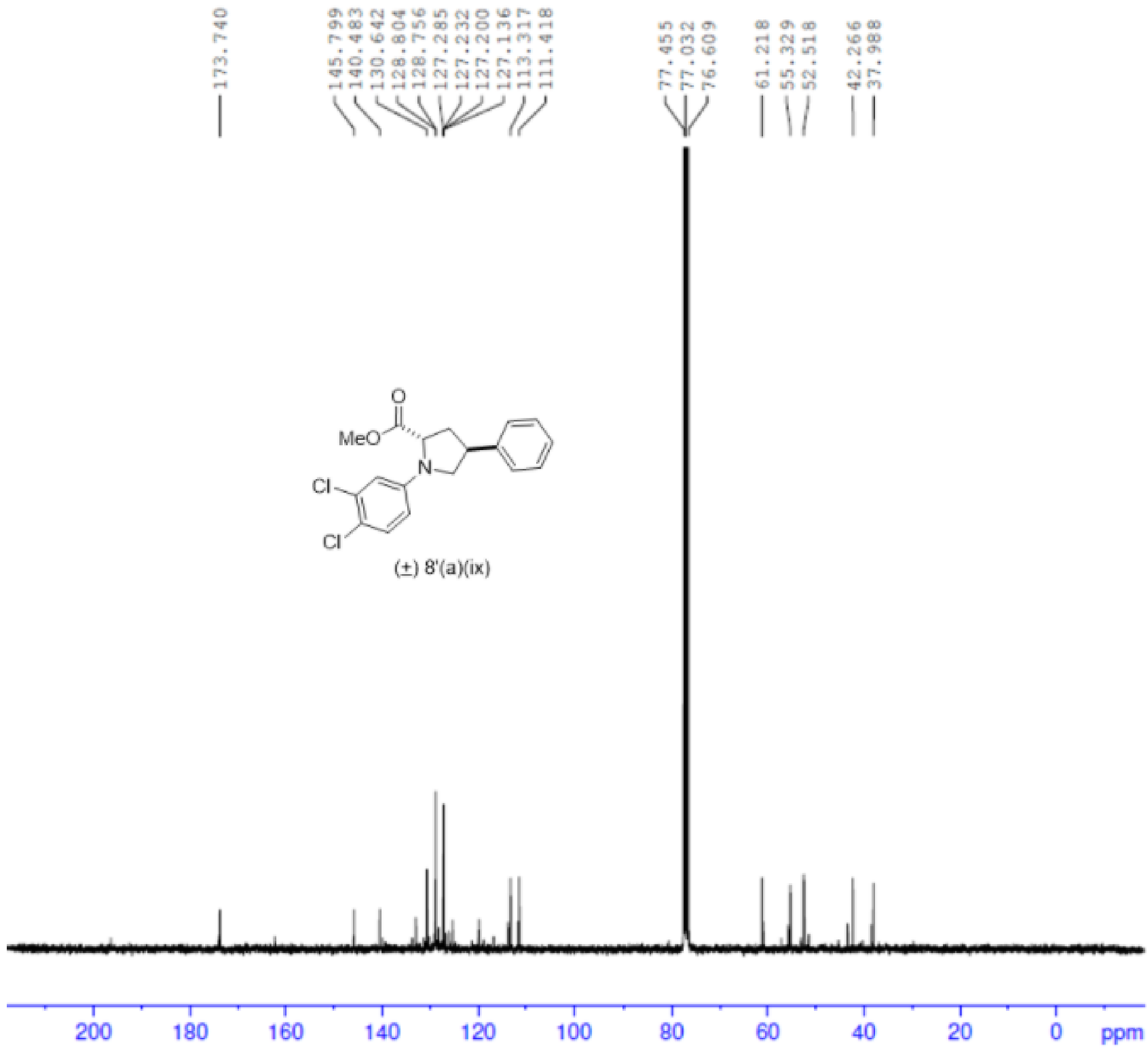

Figure S33. ^{13}C NMR spectrum of *(±) trans* 8'(a)(ix)

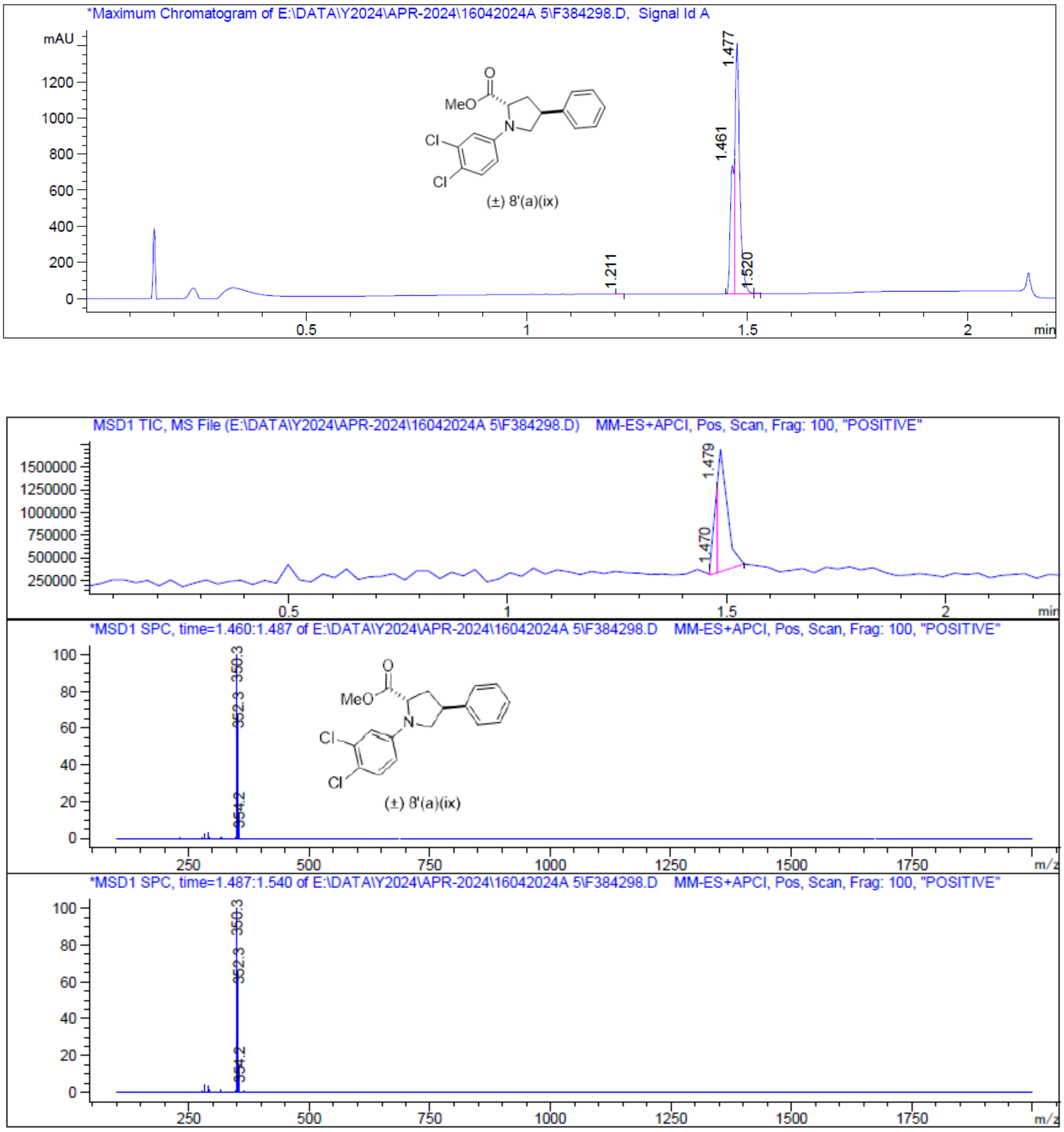

Figure S34. LCMS data of *(±) trans* 8'(a)(ix)

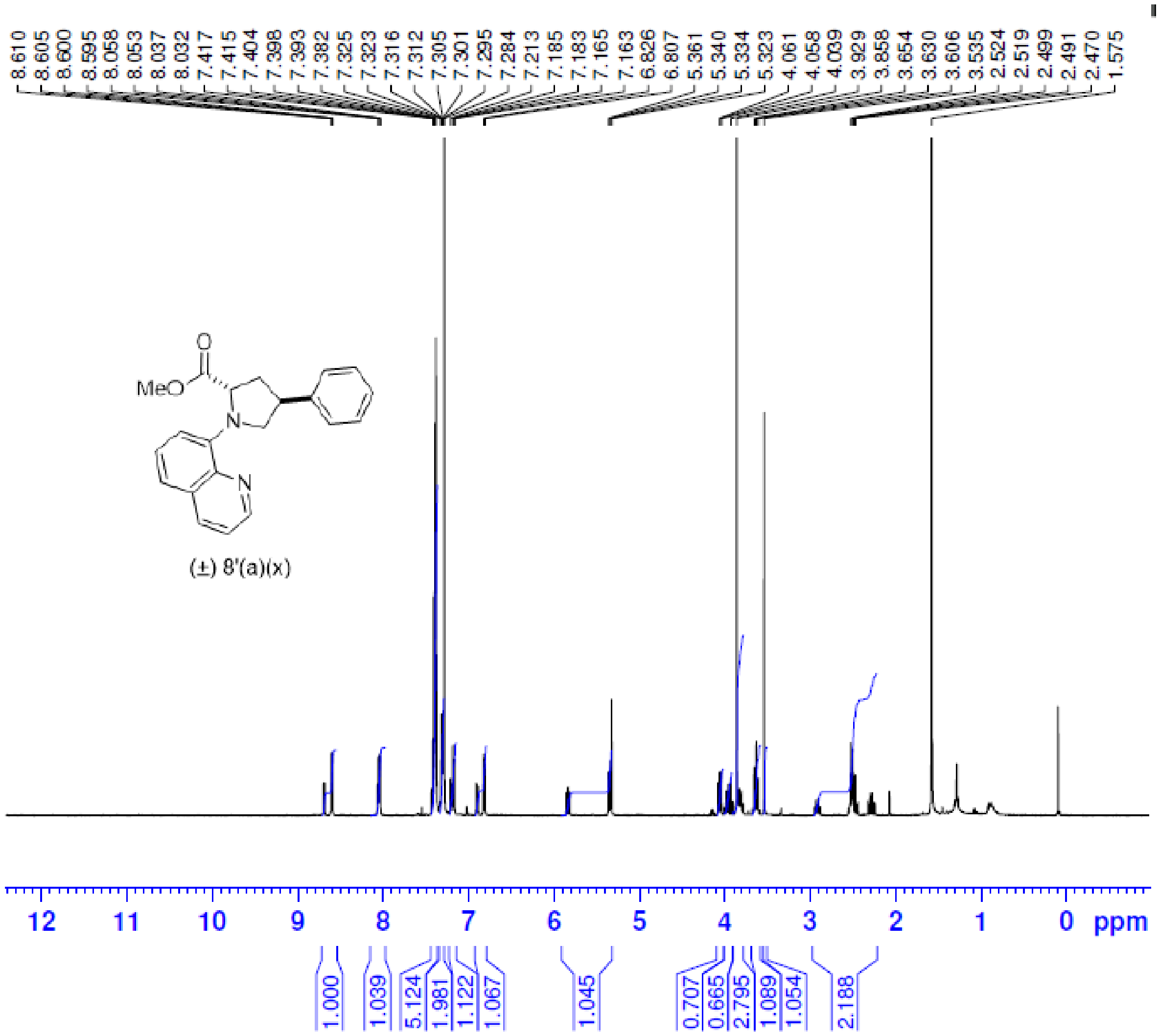

Figure S35. ^{1}H NMR spectrum of *(±) trans* 8'(a)(x)

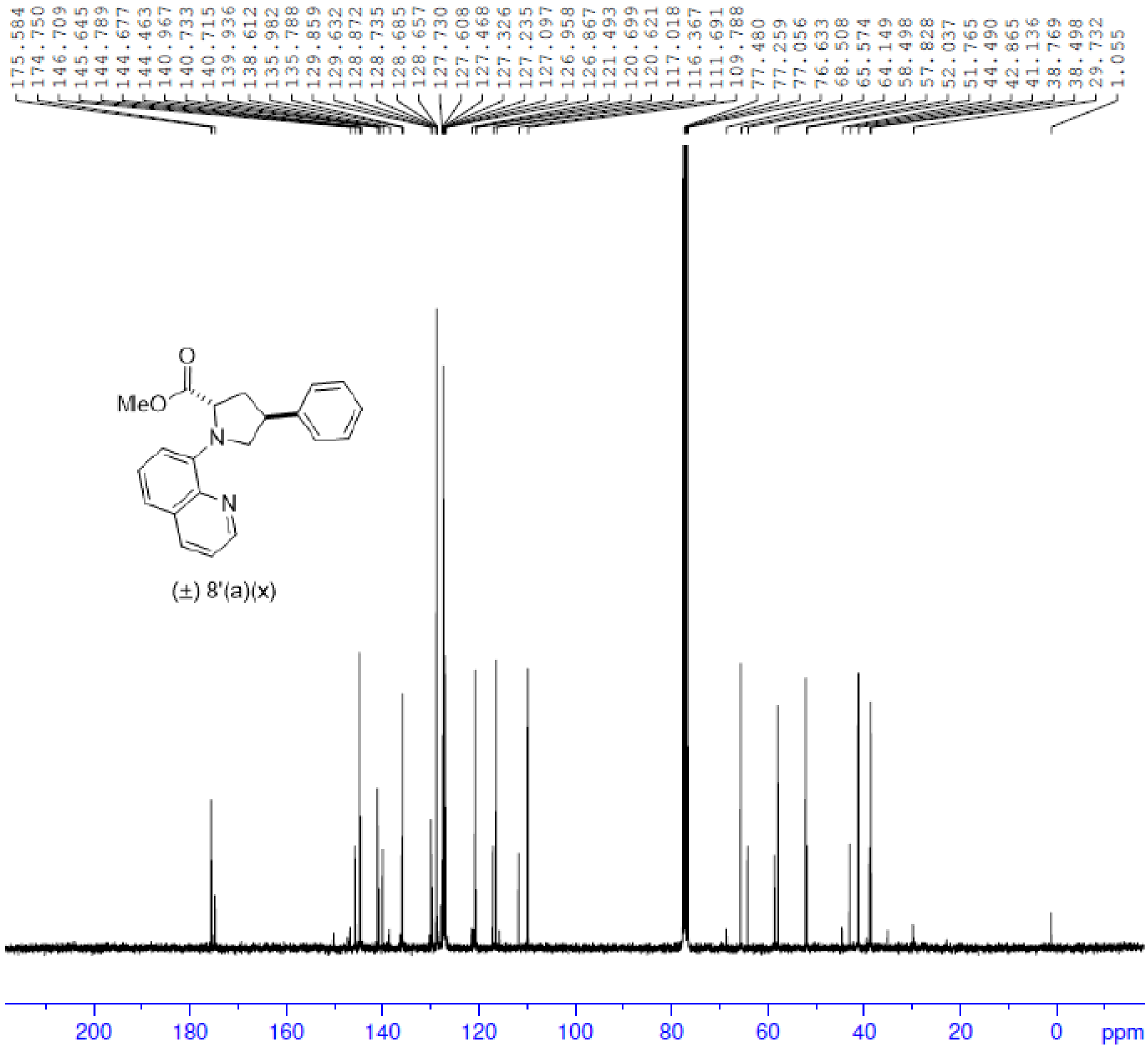

Figure S36. ^{13}C NMR spectrum of *(±) trans* 8'(a)(x)

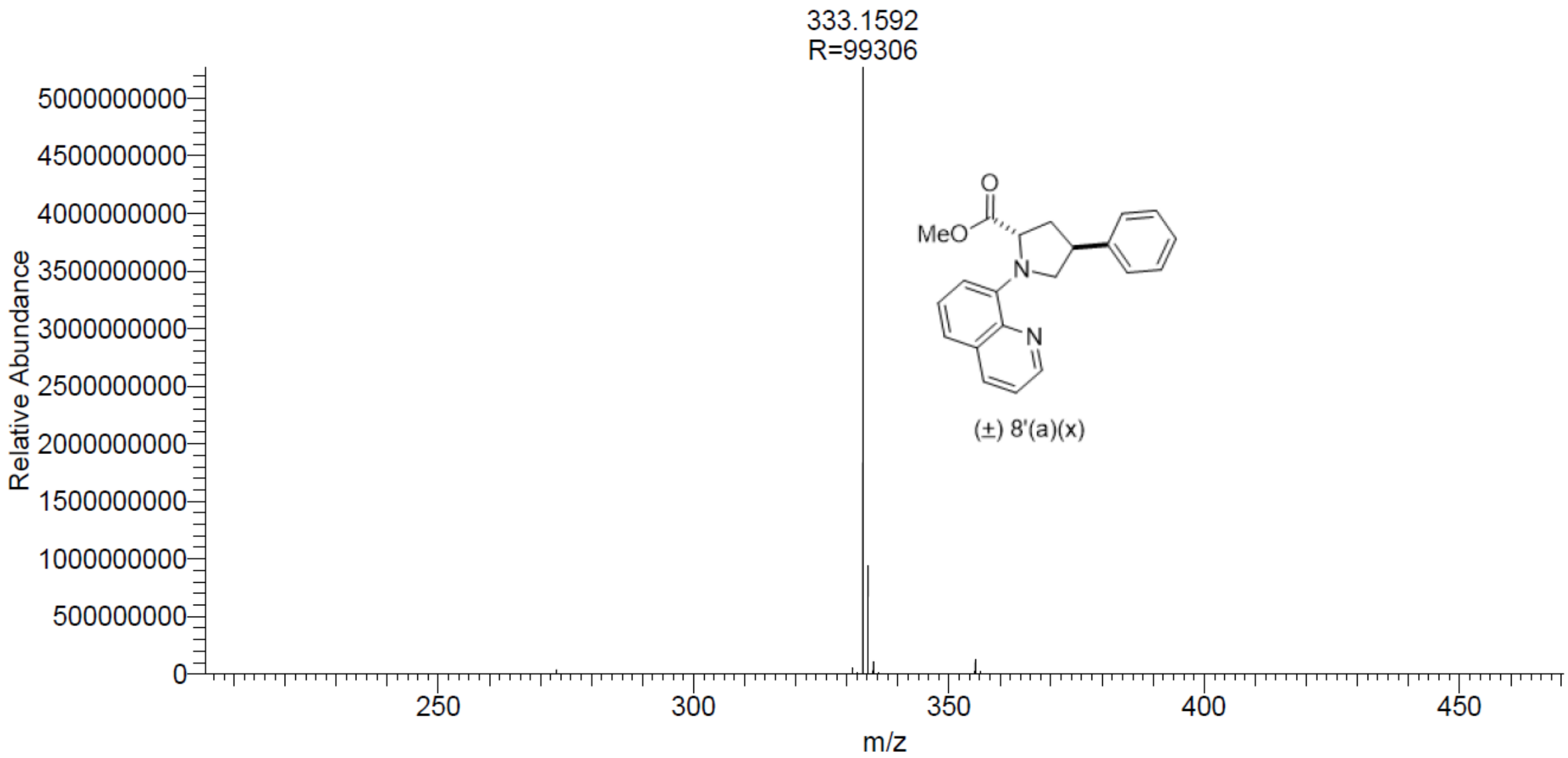

Figure S37. HRMS data of *(±) trans* 8'(a)(x)

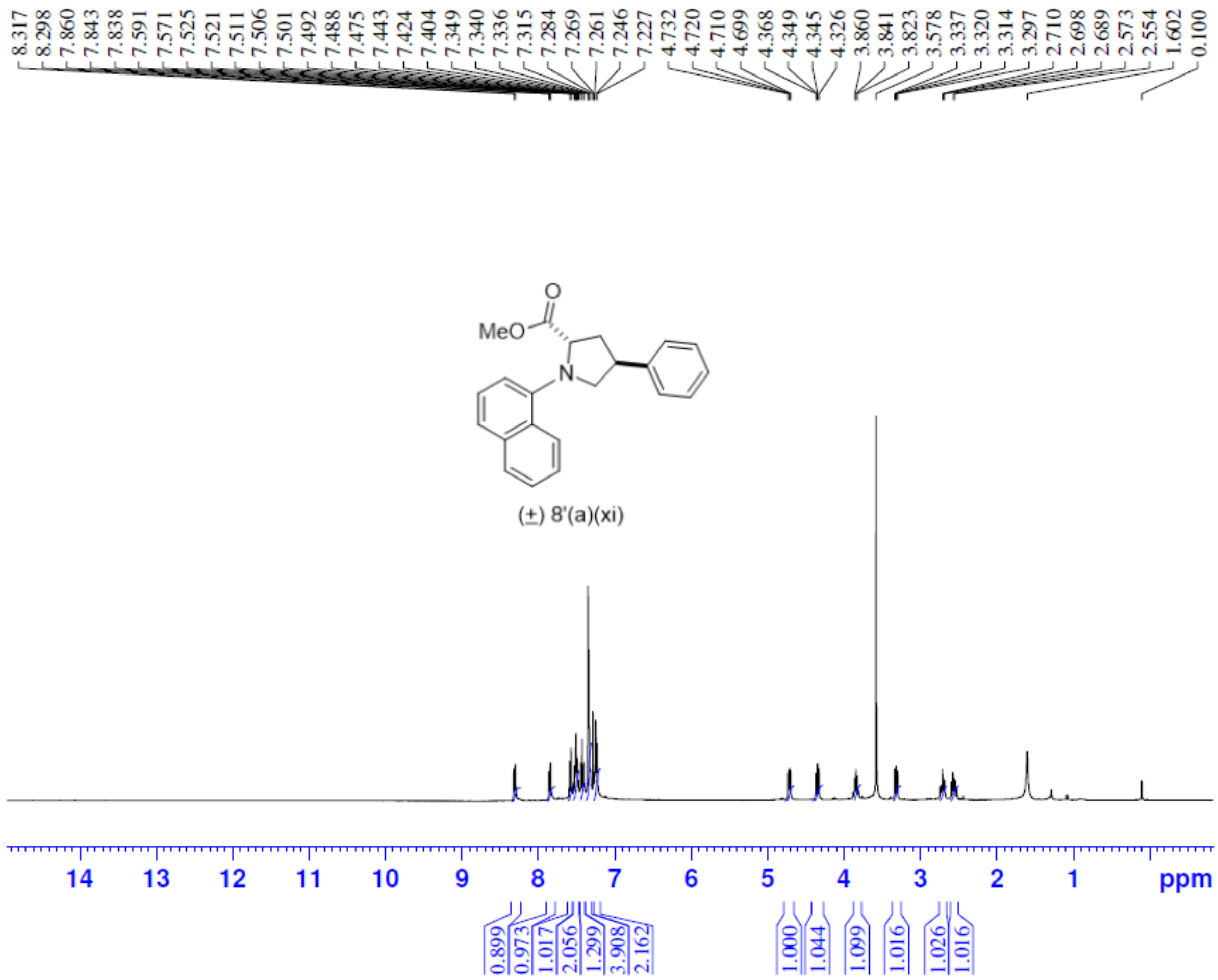

Figure S38. ^{1}H NMR spectrum of *(±) trans* 8'(a)(xi)

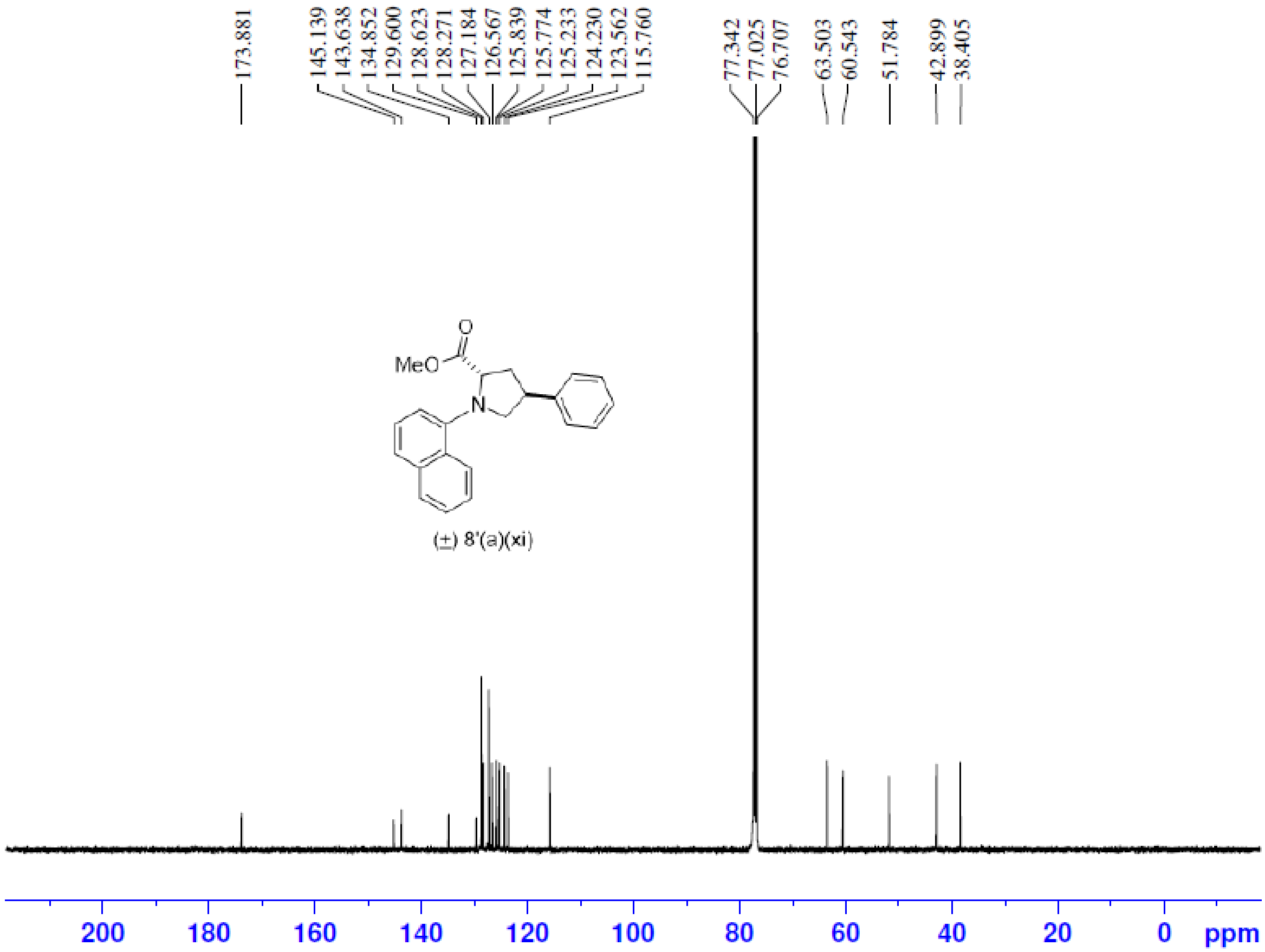

Figure S39. ^{13}C NMR spectrum of *(±) trans* 8'(a)(xi)

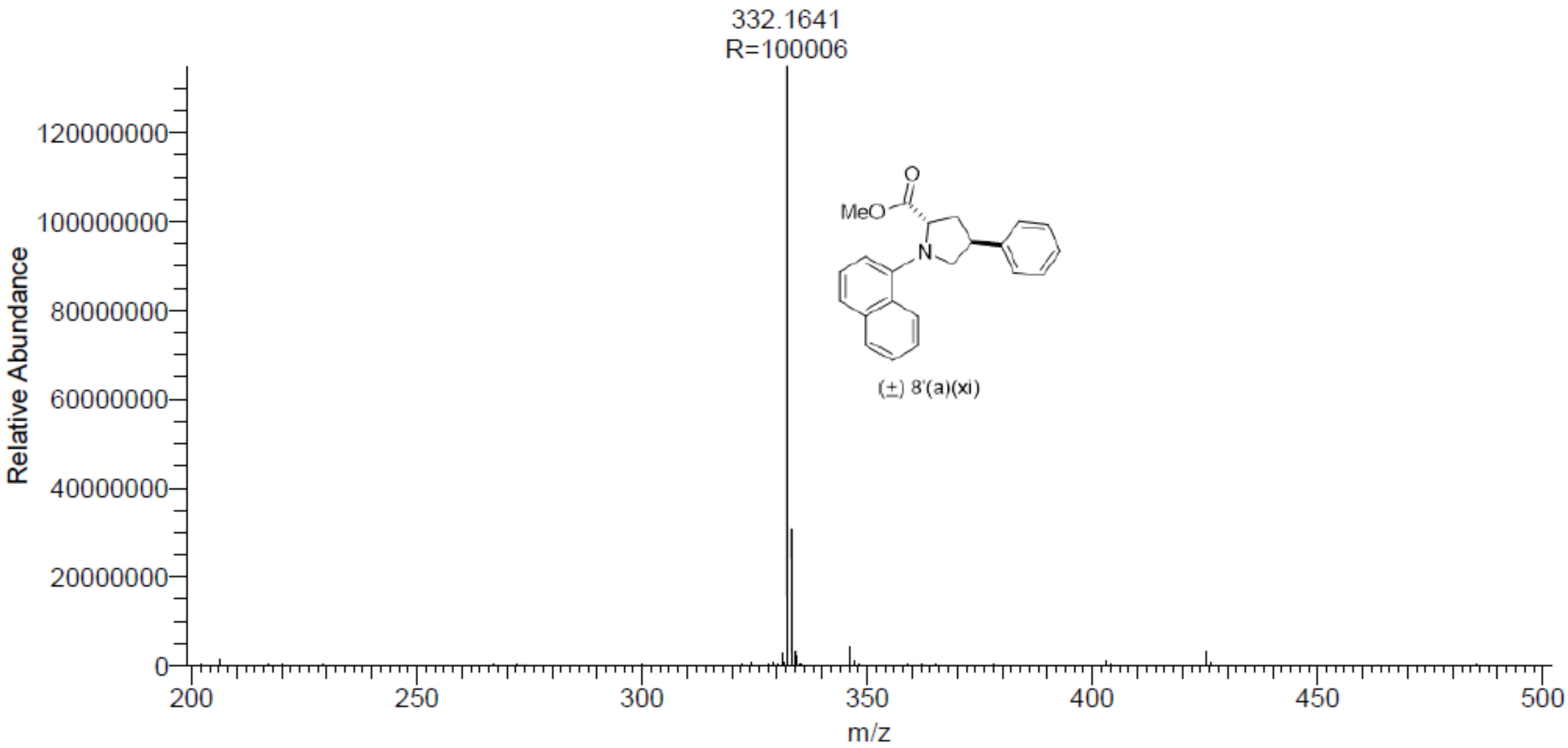

Figure S40. HRMS data of *(±) trans* 8'(a)(xi)

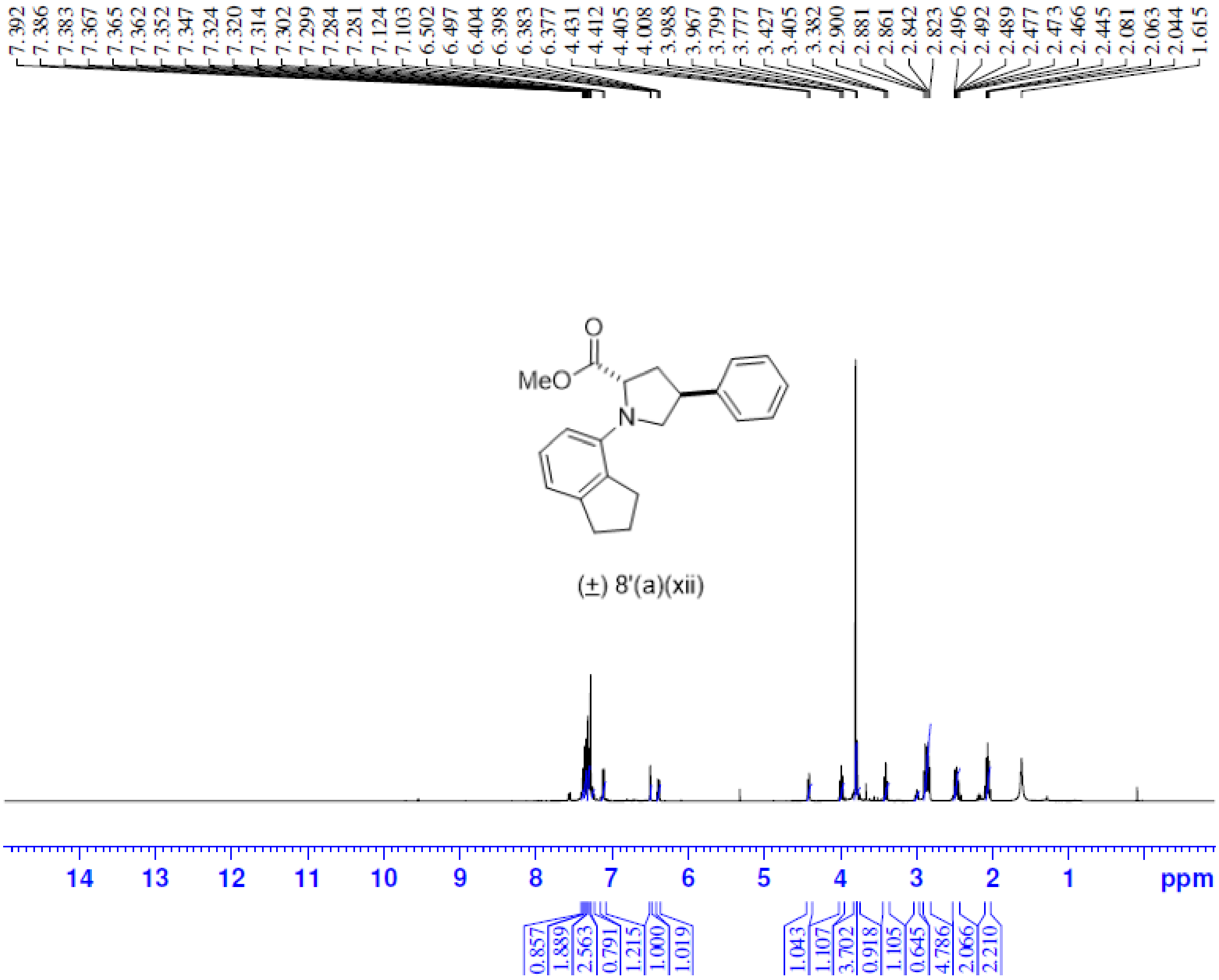

Figure S41. ^{1}H NMR spectrum of *(±) trans* 8'(a)(xii)

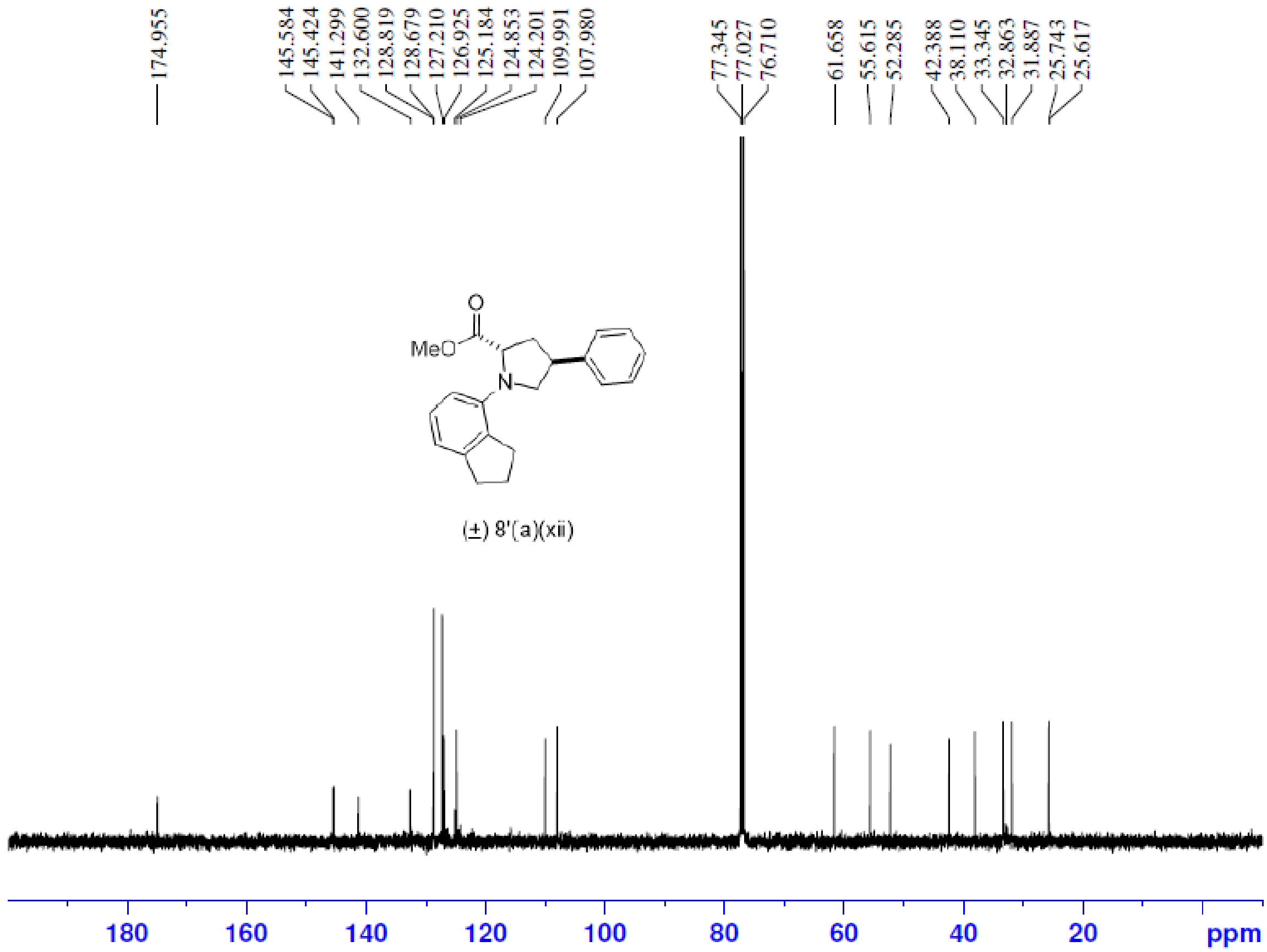

Figure S42. ^{13}C NMR spectrum of *(±) trans* 8'(a)(xii)

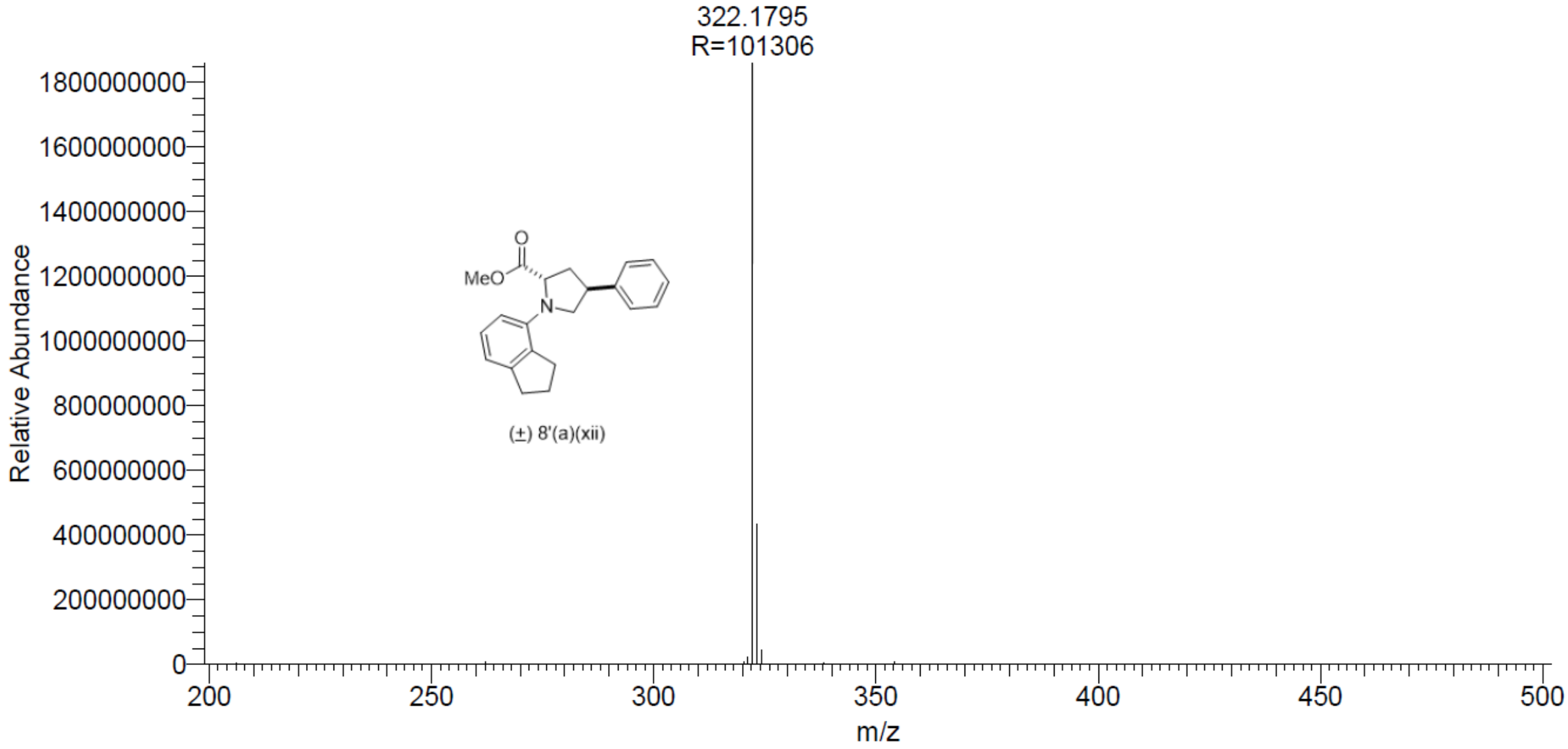

Figure S43. HRMS data of *(±) trans* 8'(a)(xii)

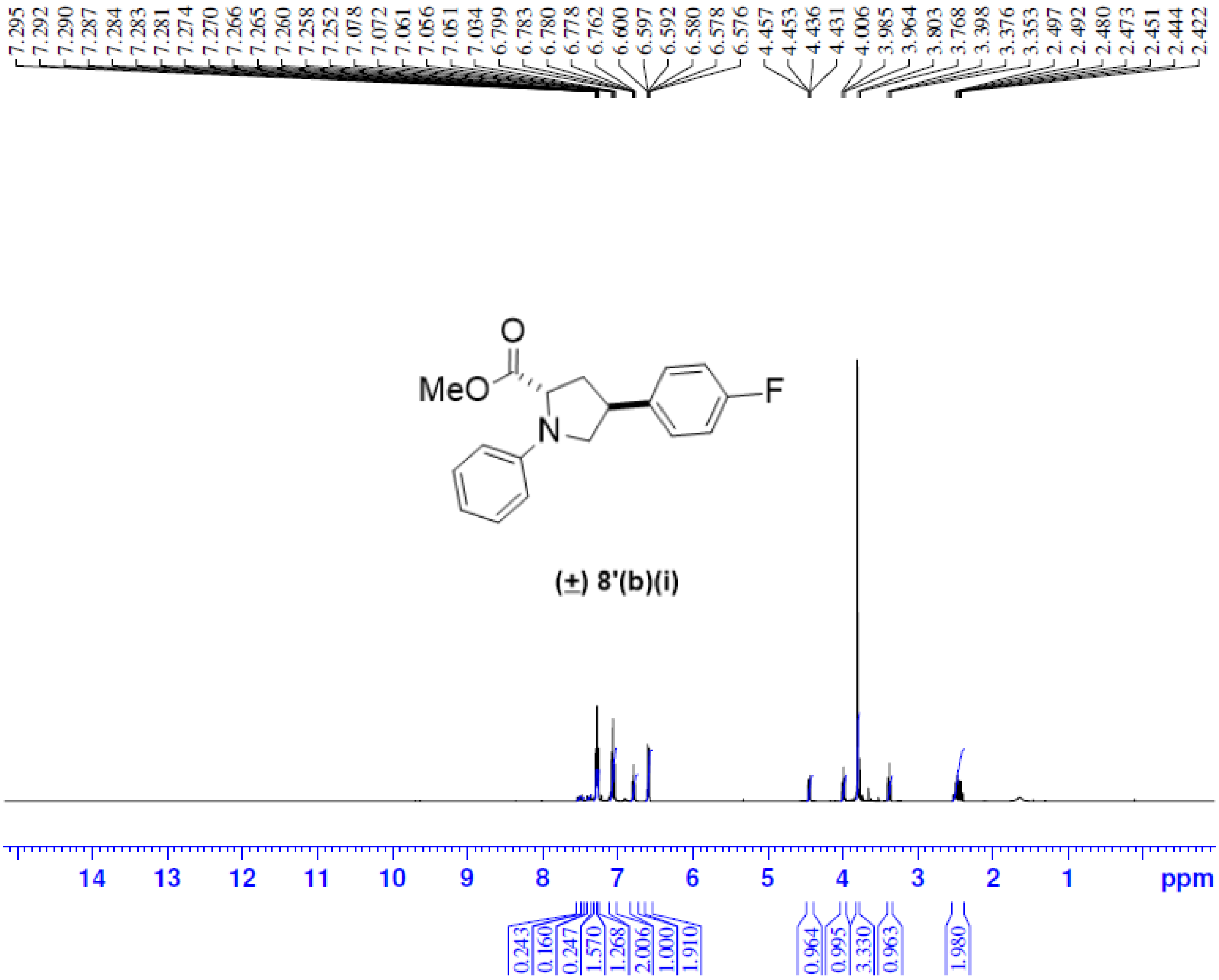

Figure S44. ^{1}H NMR spectrum of *(±) trans* 8'(b)(i)

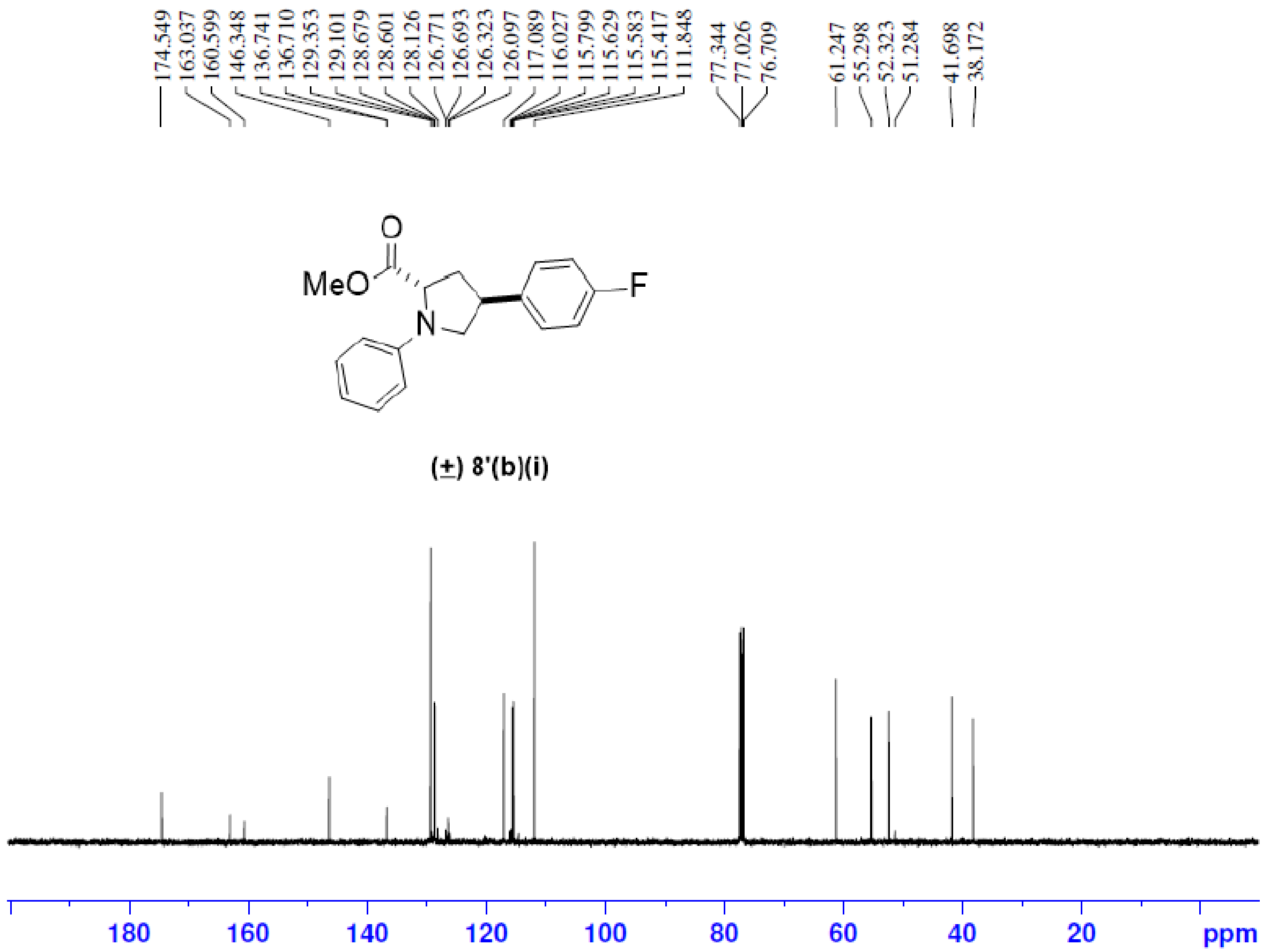

Figure S45. ^{13}C NMR spectrum of *(±) trans* 8'(b)(i)

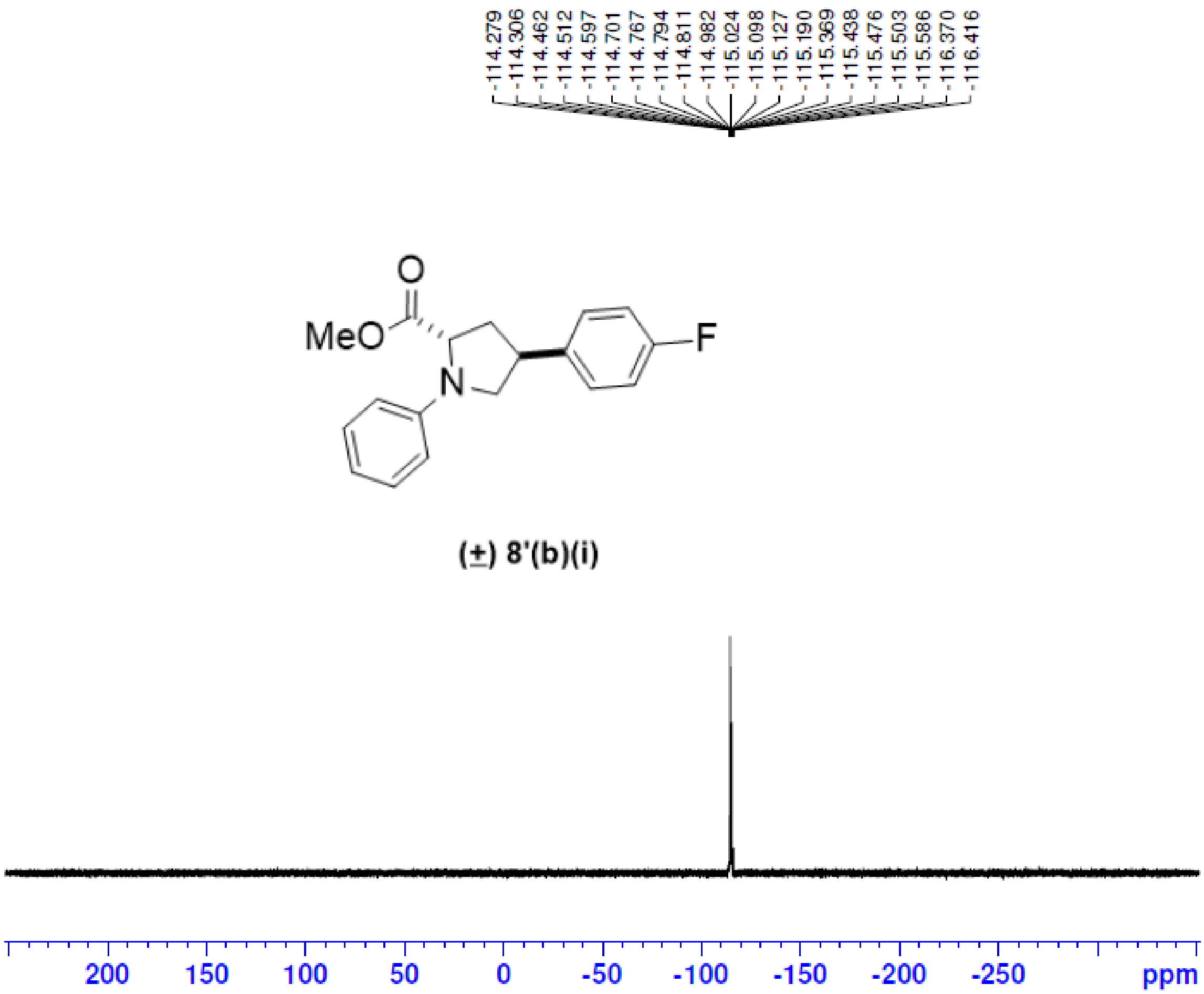

Figure S46. ^{19}F NMR spectrum of *(±) trans* 8'(b)(i)

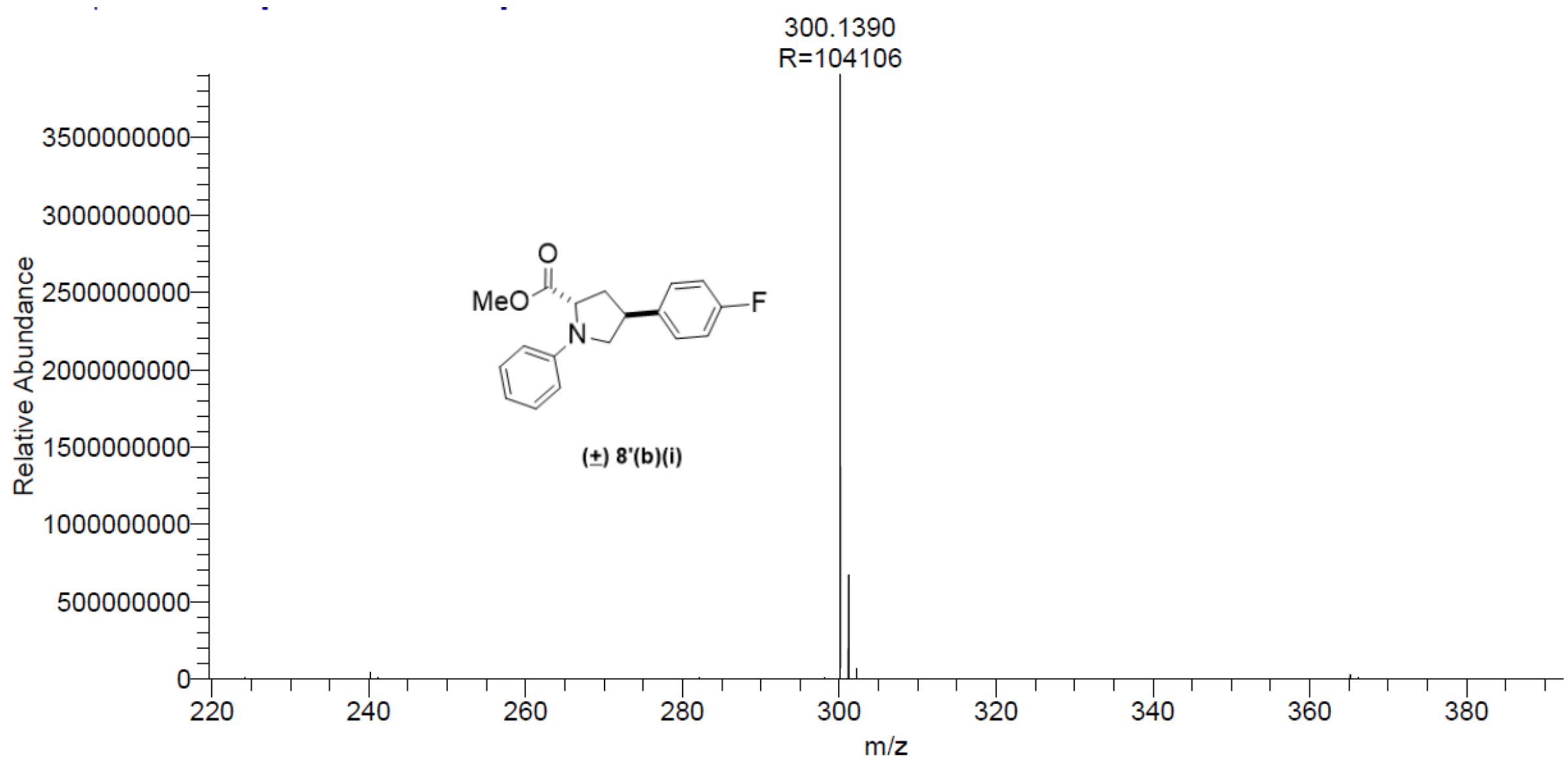

Figure S47. HRMS data of *(±) trans* 8'(b)(i)

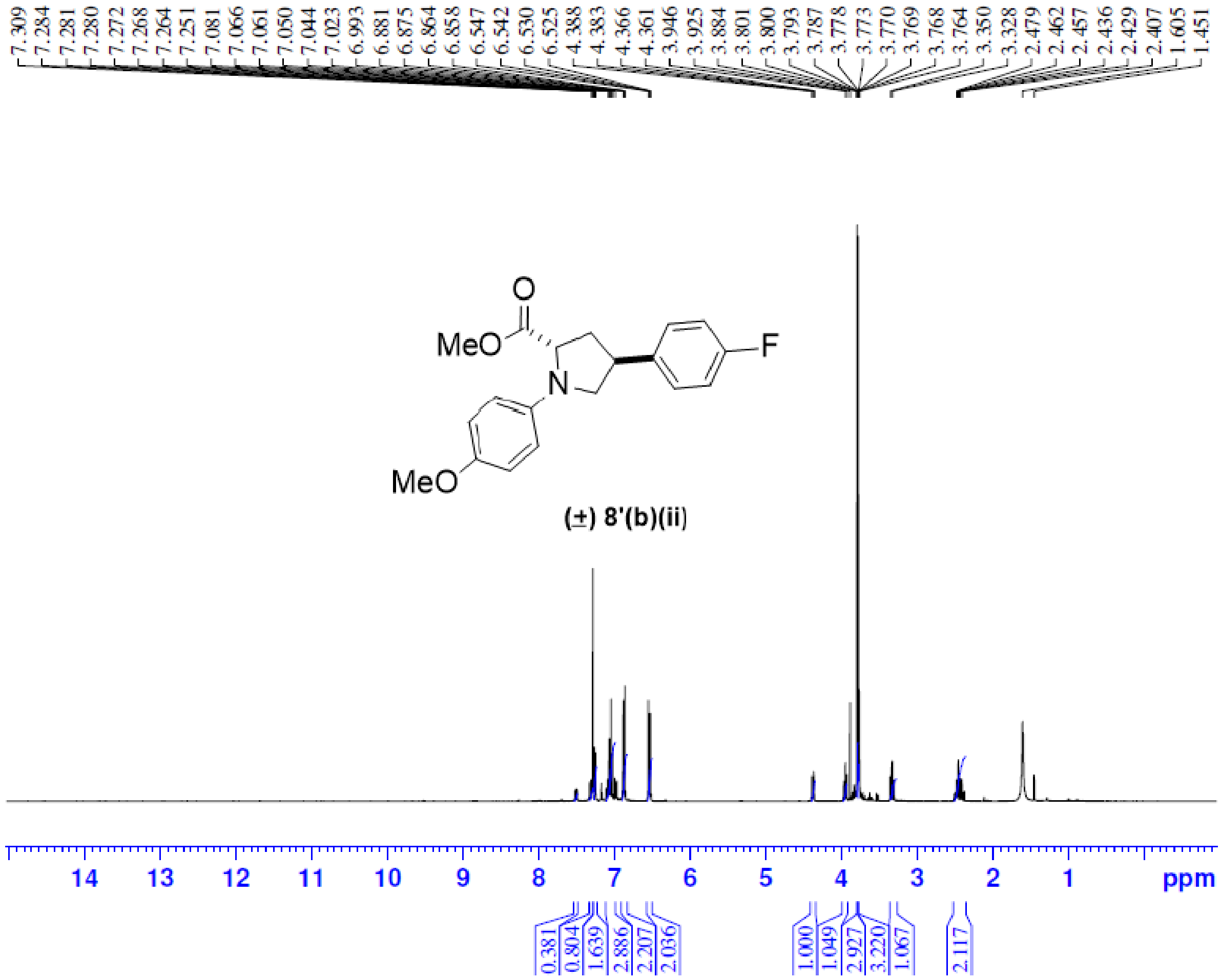

Figure S48. ^{1}H NMR spectrum of *(±) trans* 8’(b)(ii)

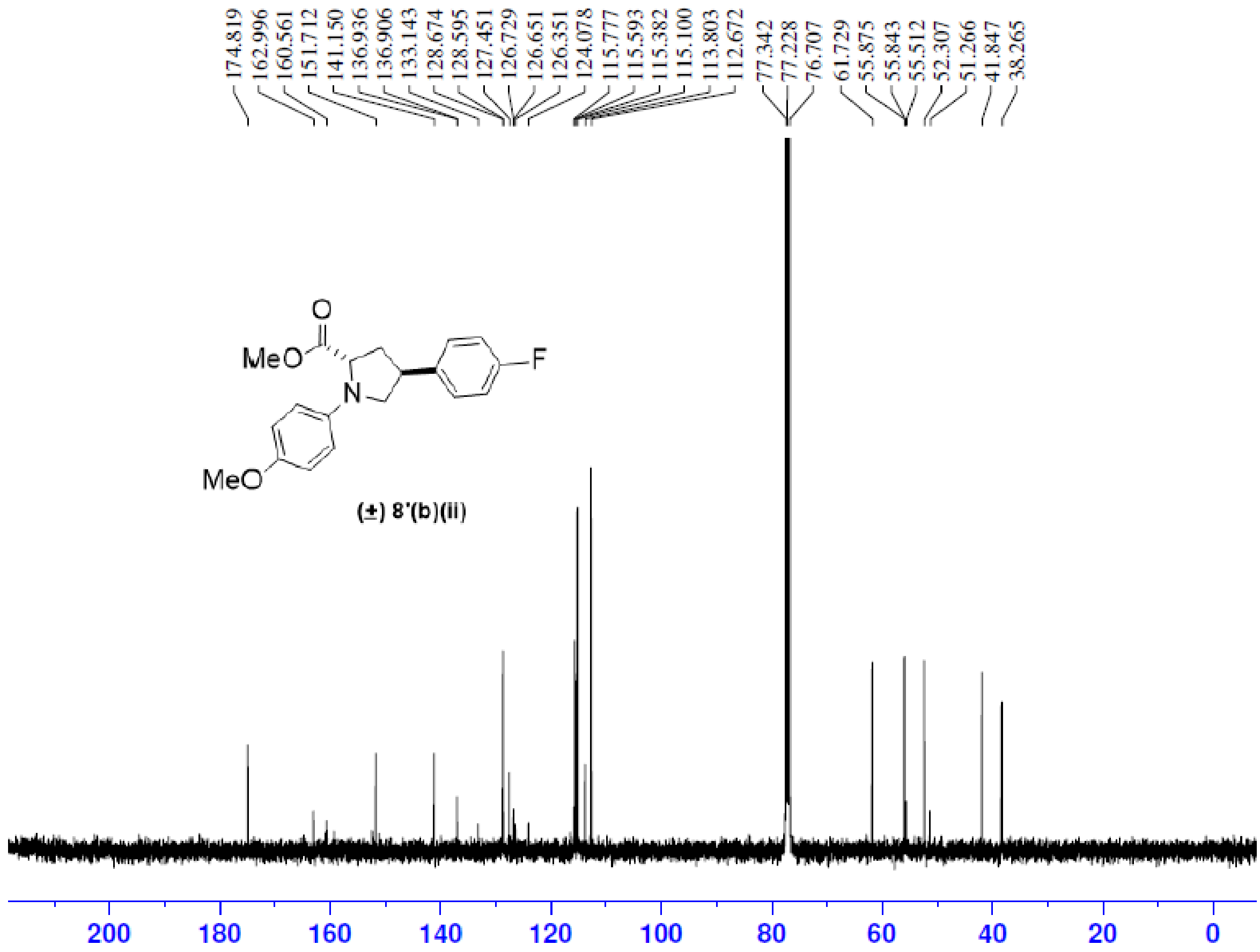

Figure S49. ^{13}C NMR spectrum of *(±) trans* 8'(b)(ii)

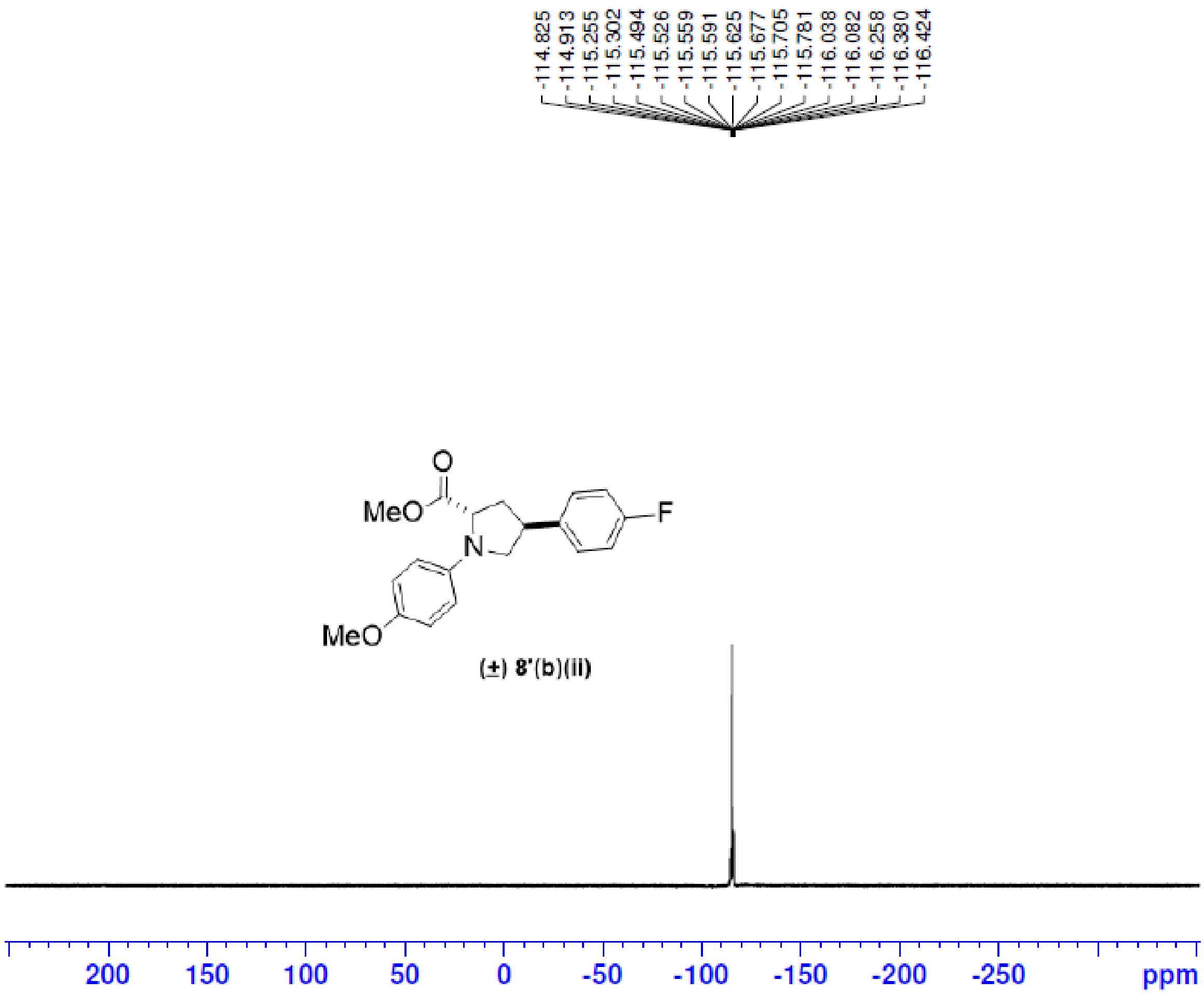

Figure S50. ^{19}F NMR spectrum of *(±) trans* 8'(b)(ii)

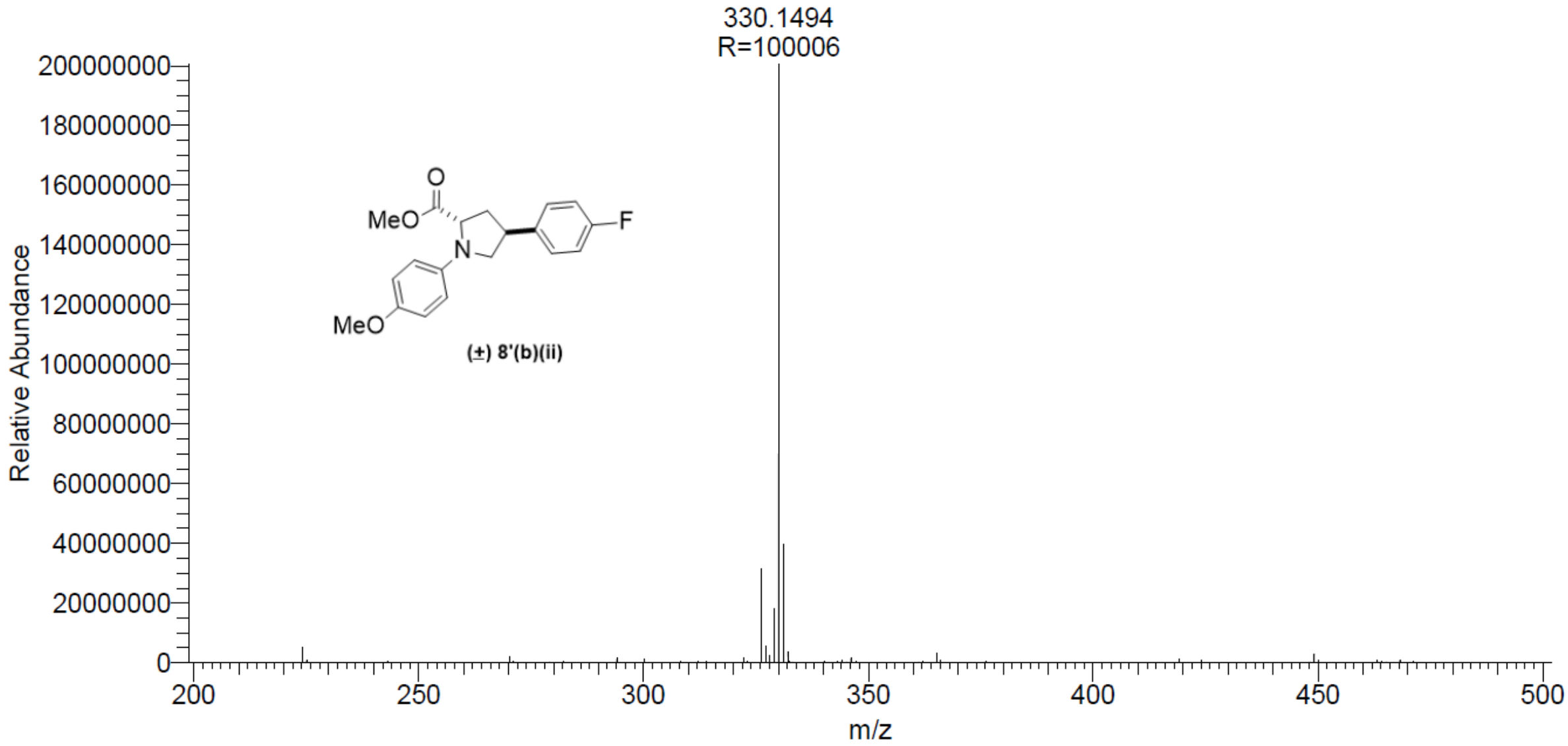

Figure S51. HRMS data of *(±) trans* 8'(b)(ii)

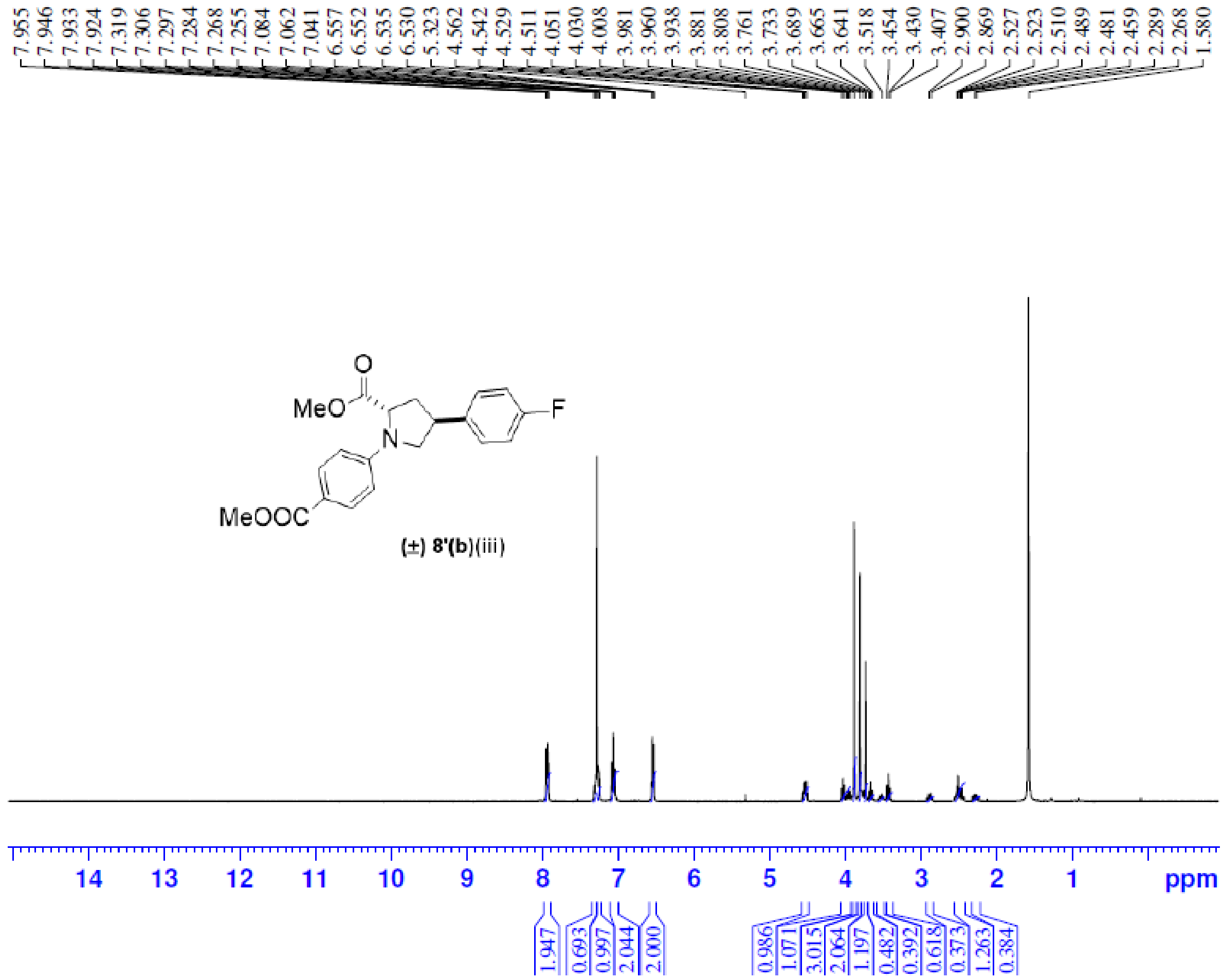

Figure S52. ^{1}H NMR spectrum of *(±) trans* 8'(b)(iii)

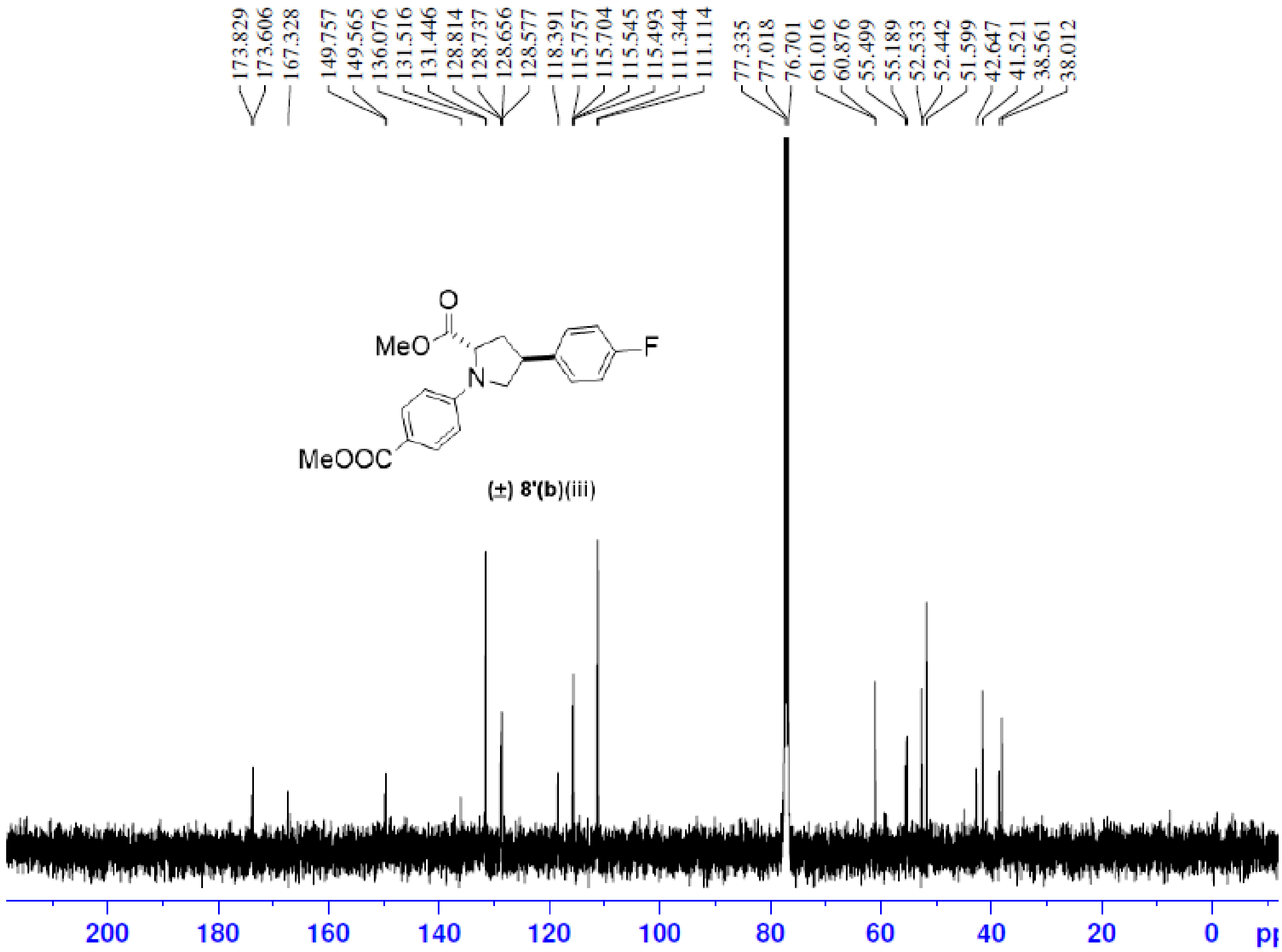

Figure S53. ^{13}C NMR spectrum of *(±) trans* 8’(b)(iii)

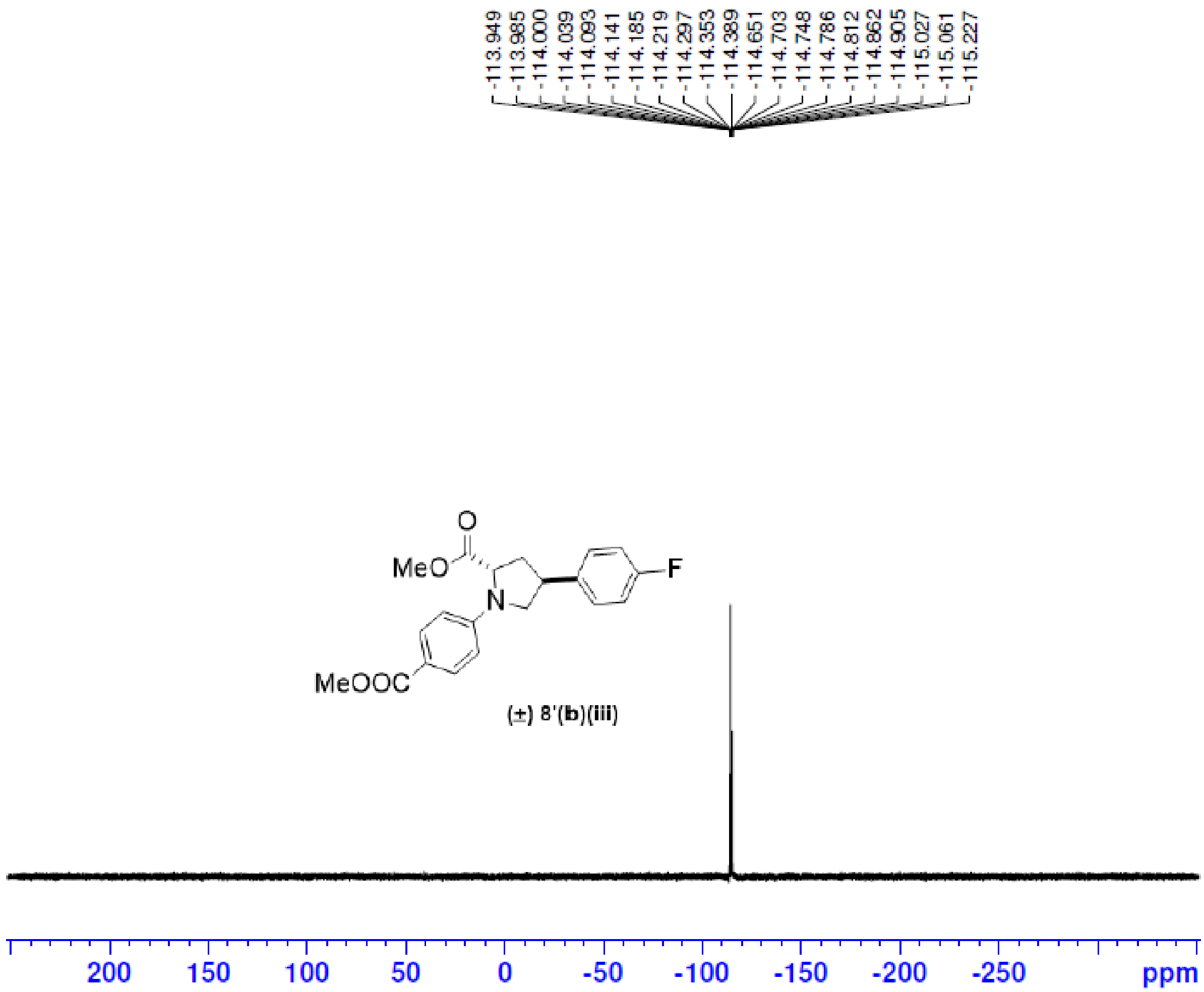

Figure S54. ^{19}F NMR spectrum of *(±) trans* 8’(b)(iii)

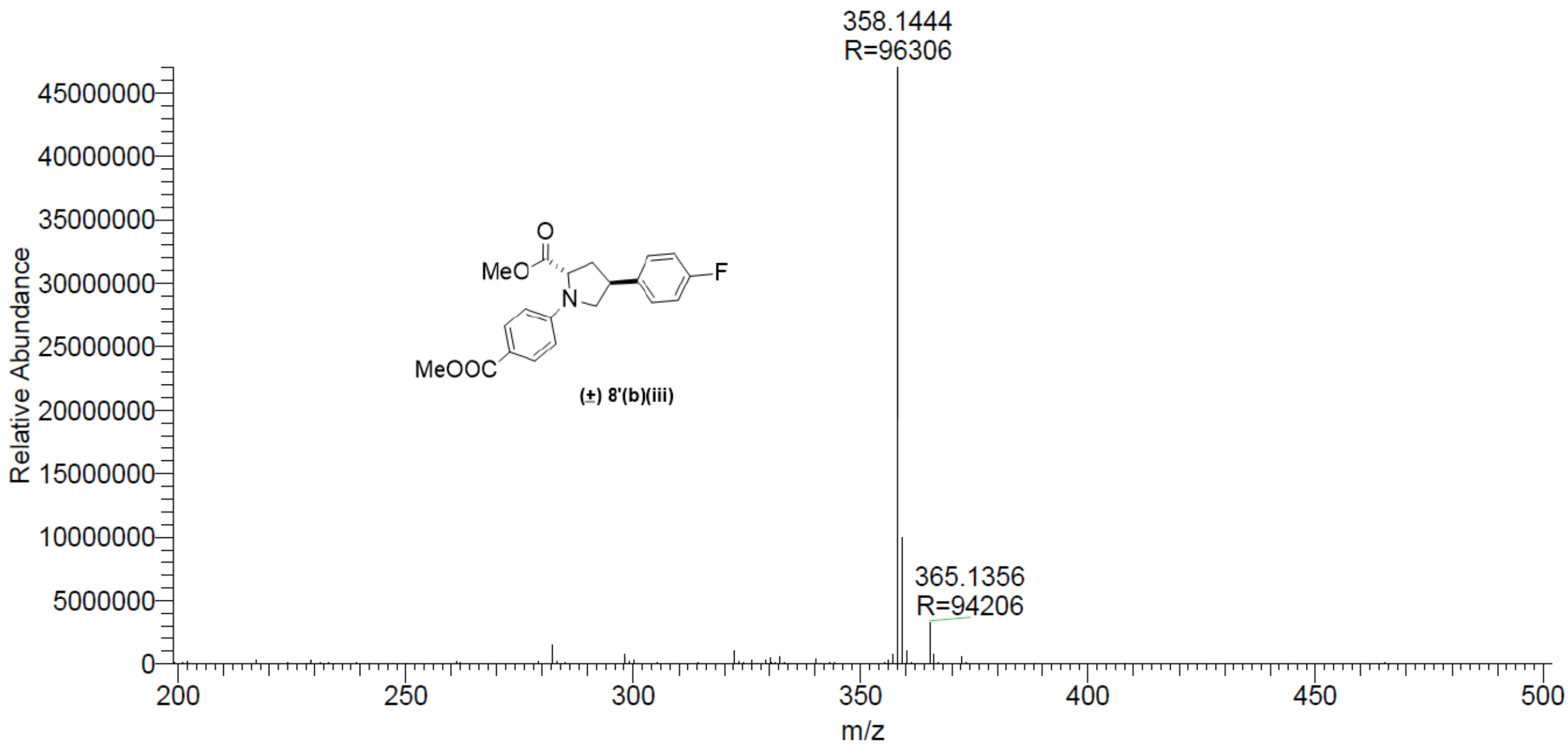

Figure S55. HRMS data of *(±) trans* 8’(b)(iii)

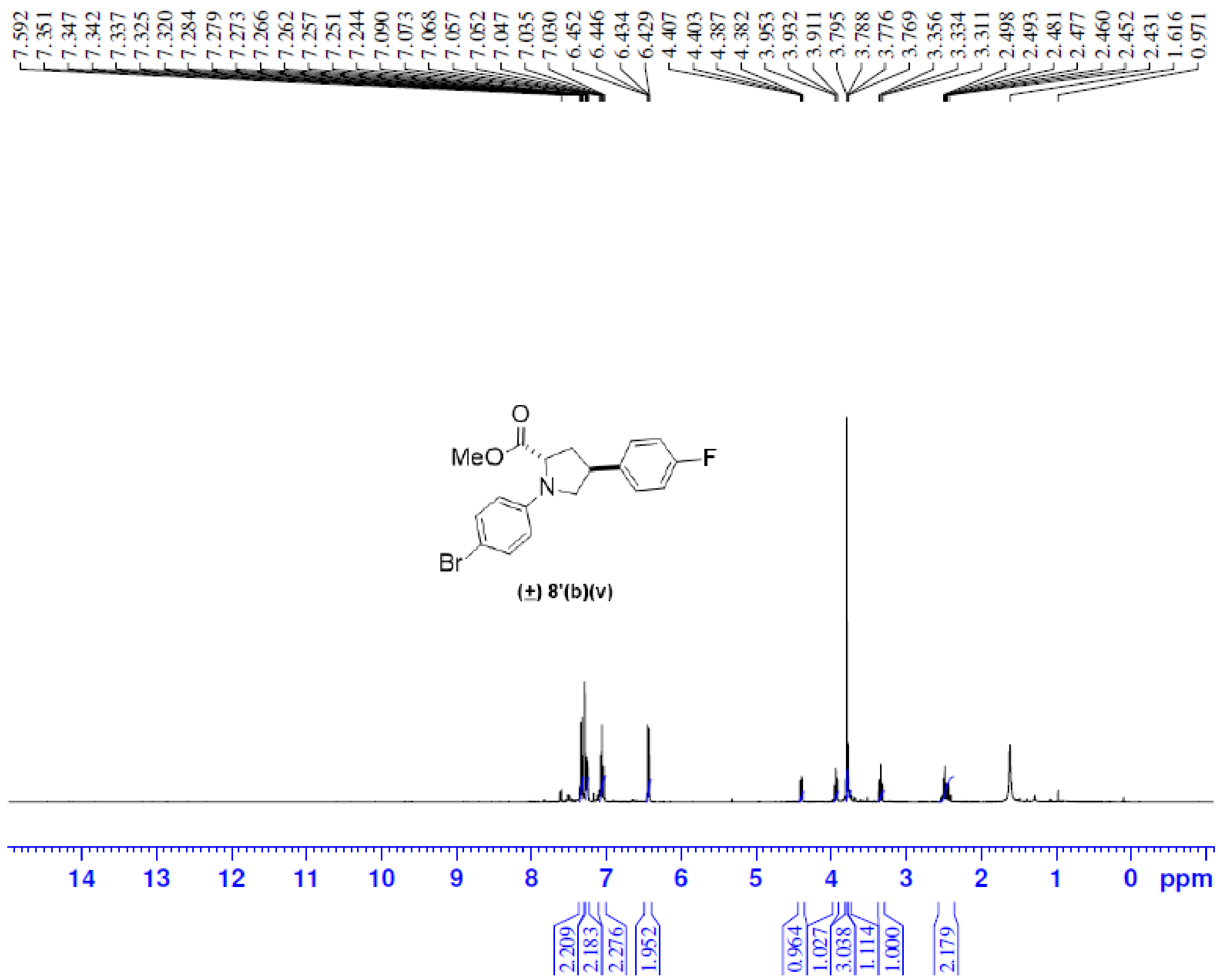

Figure S56. ^{1}H NMR spectrum of *(±) trans* 8'(b)(v)

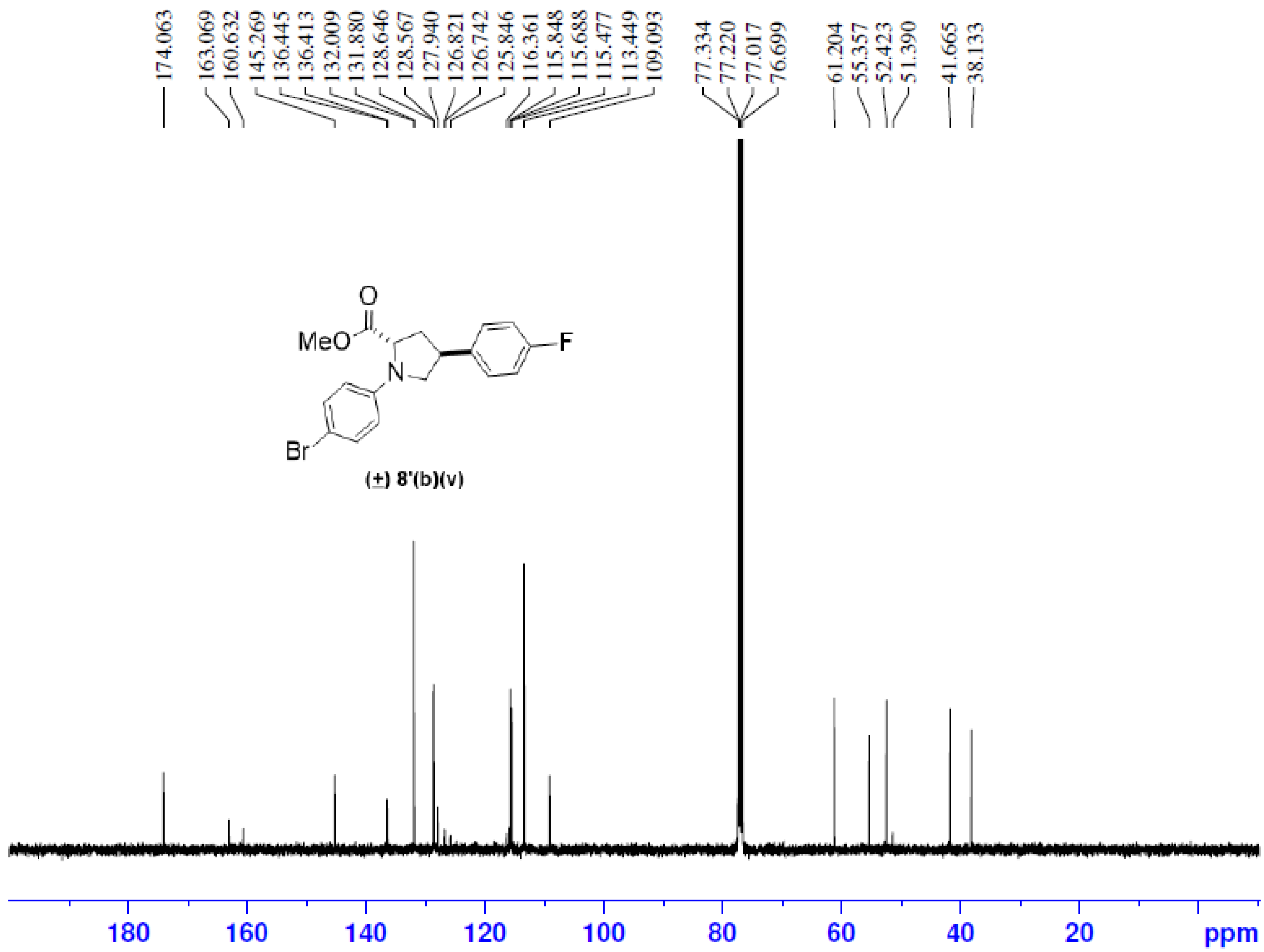

Figure S57. ^{13}C NMR spectrum of *(±) trans* 8’(b)(v)

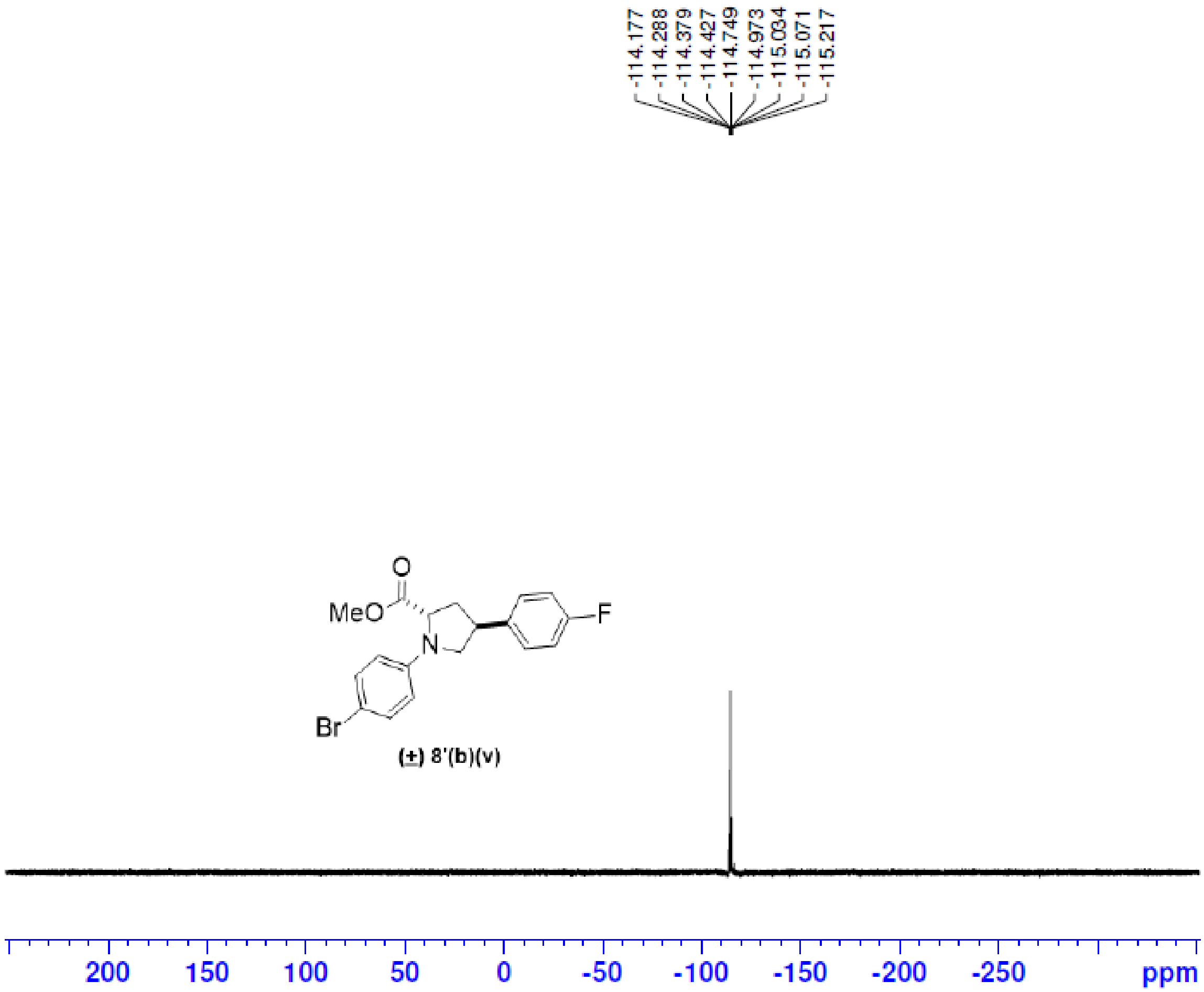

Figure S58. ^{19}F NMR spectrum of *(±) trans* 8'(b)(v)

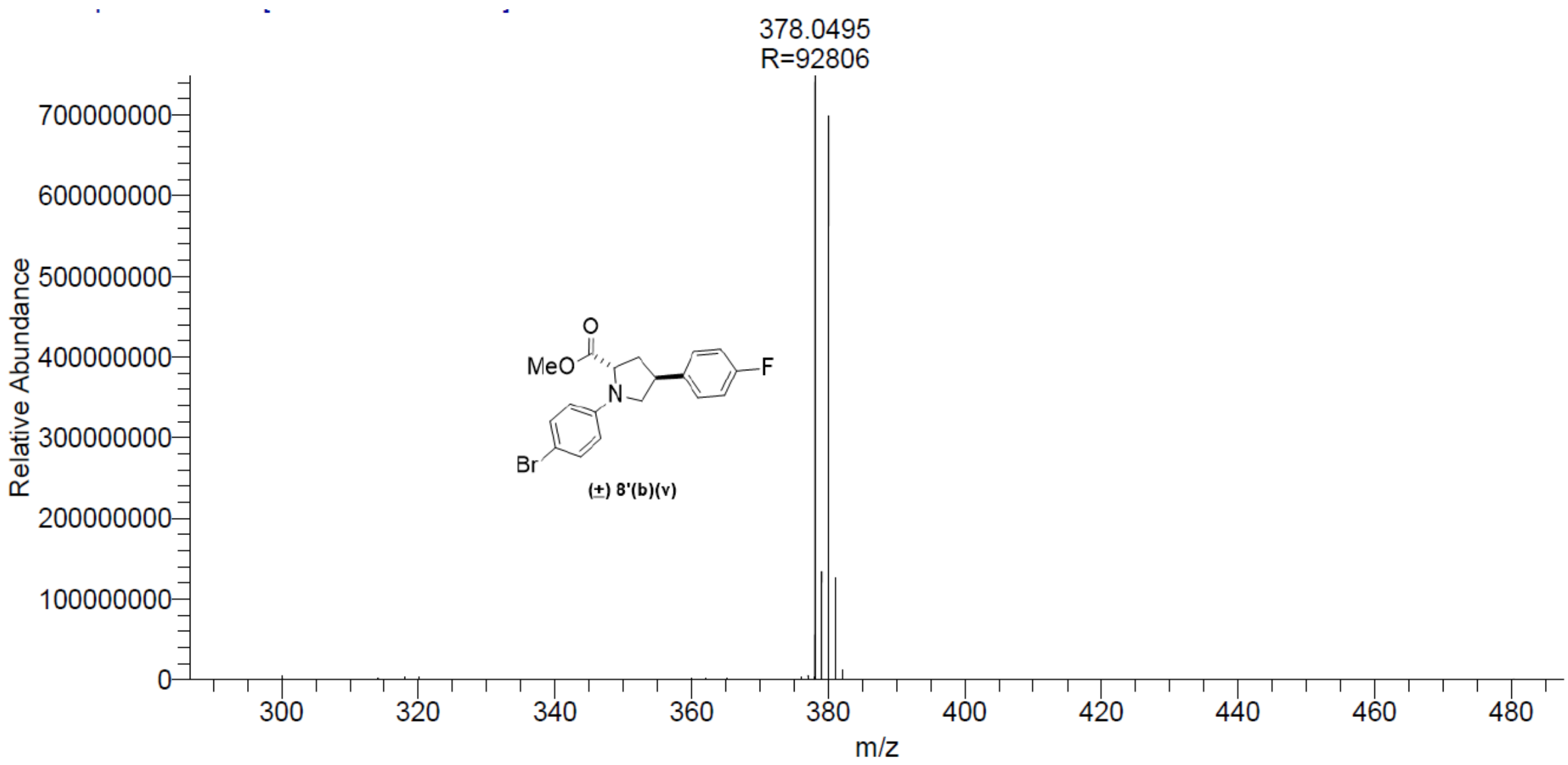

Figure S59. HRMS data of *(±) trans* 8'(b)(v)

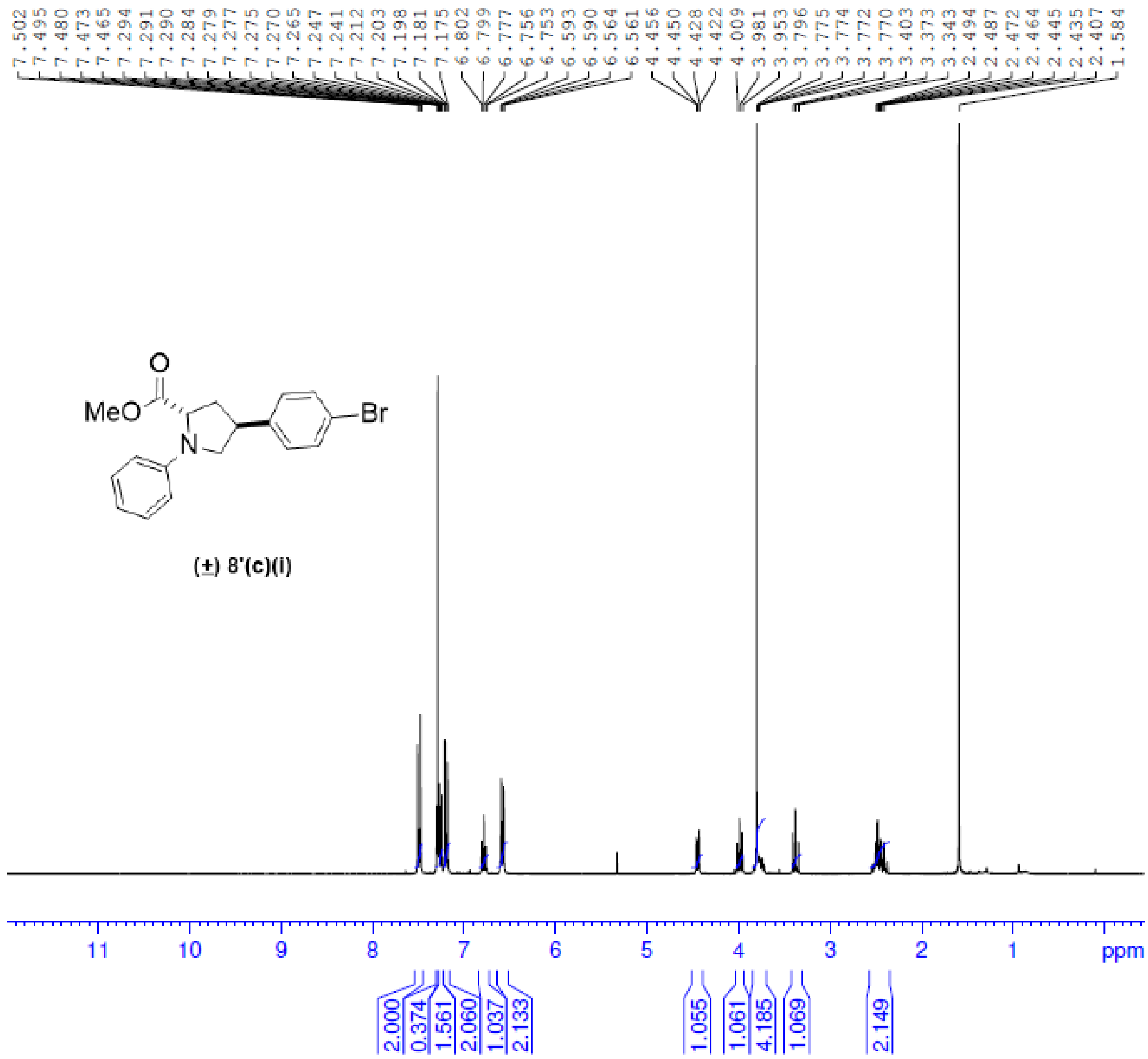

Figure S60. ^{1}H NMR spectrum of *(±) trans* 8'(c)(i)

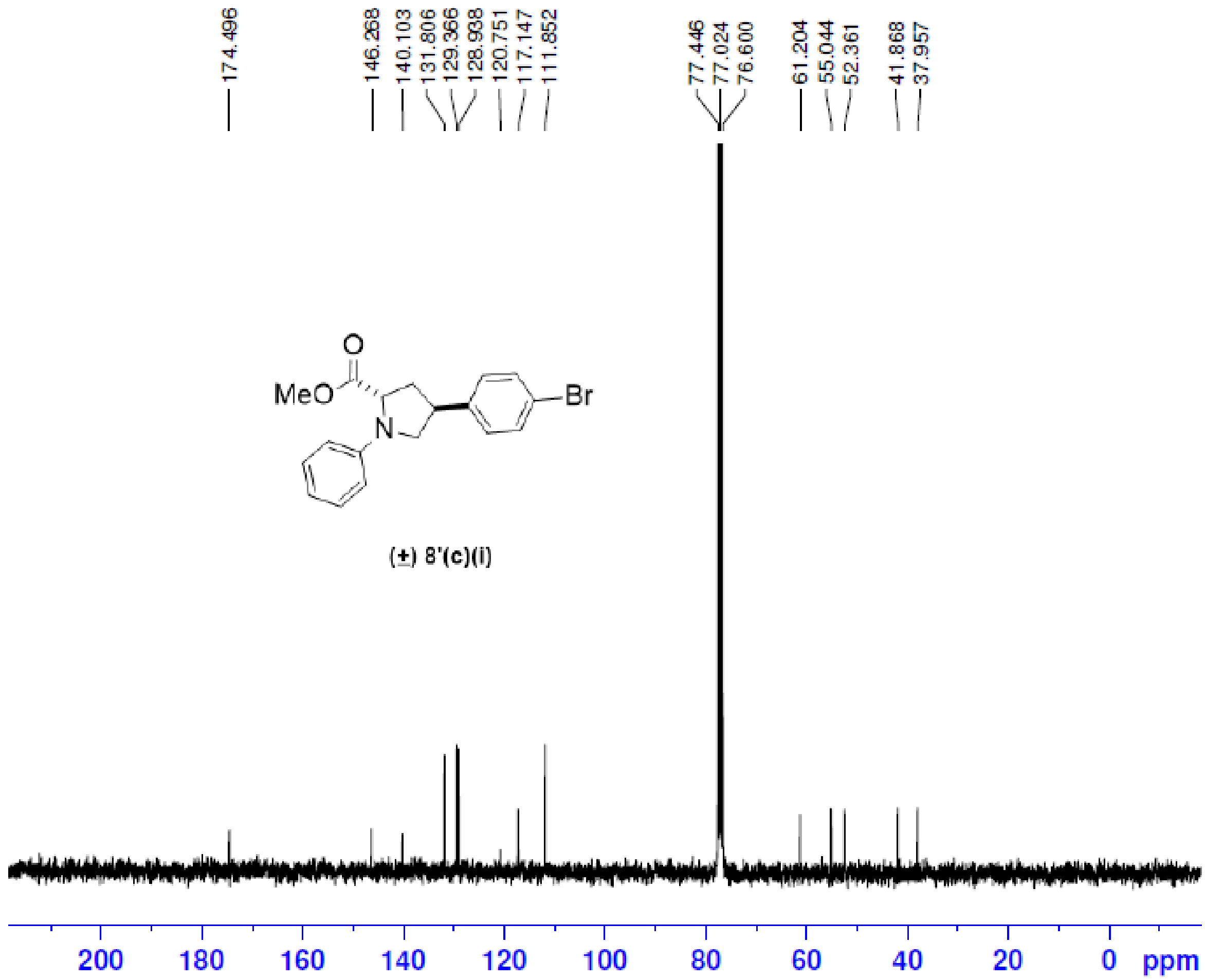

Figure S61. ^{13}C NMR spectrum of *(±) trans* 8'(c)(i)

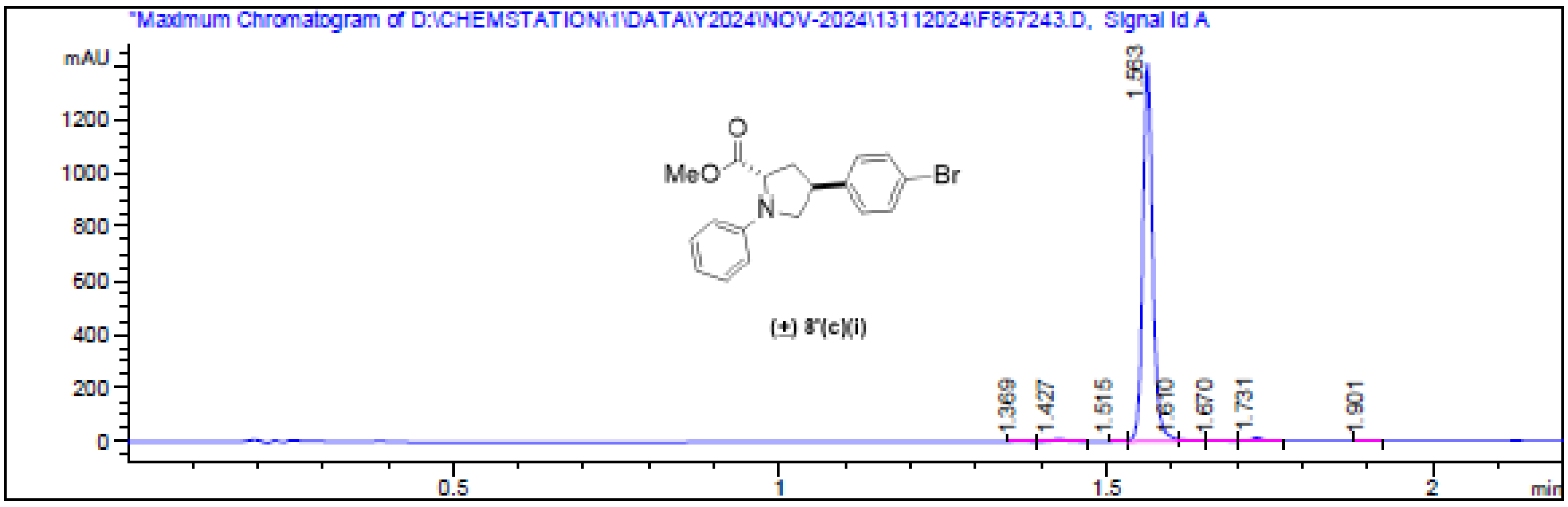

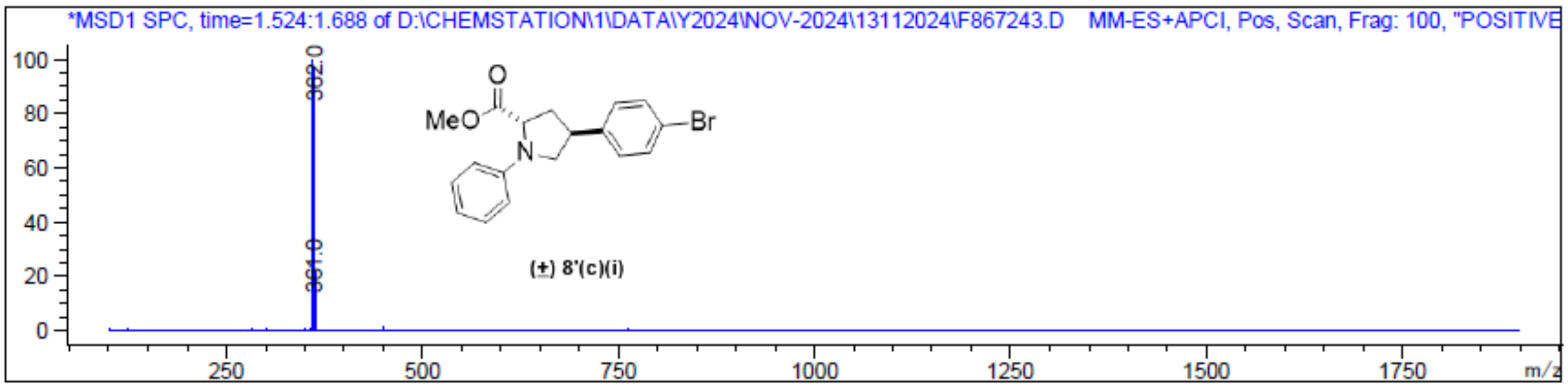

Figure S62. LC-MS data of *(±) trans* 8'(c)(i)

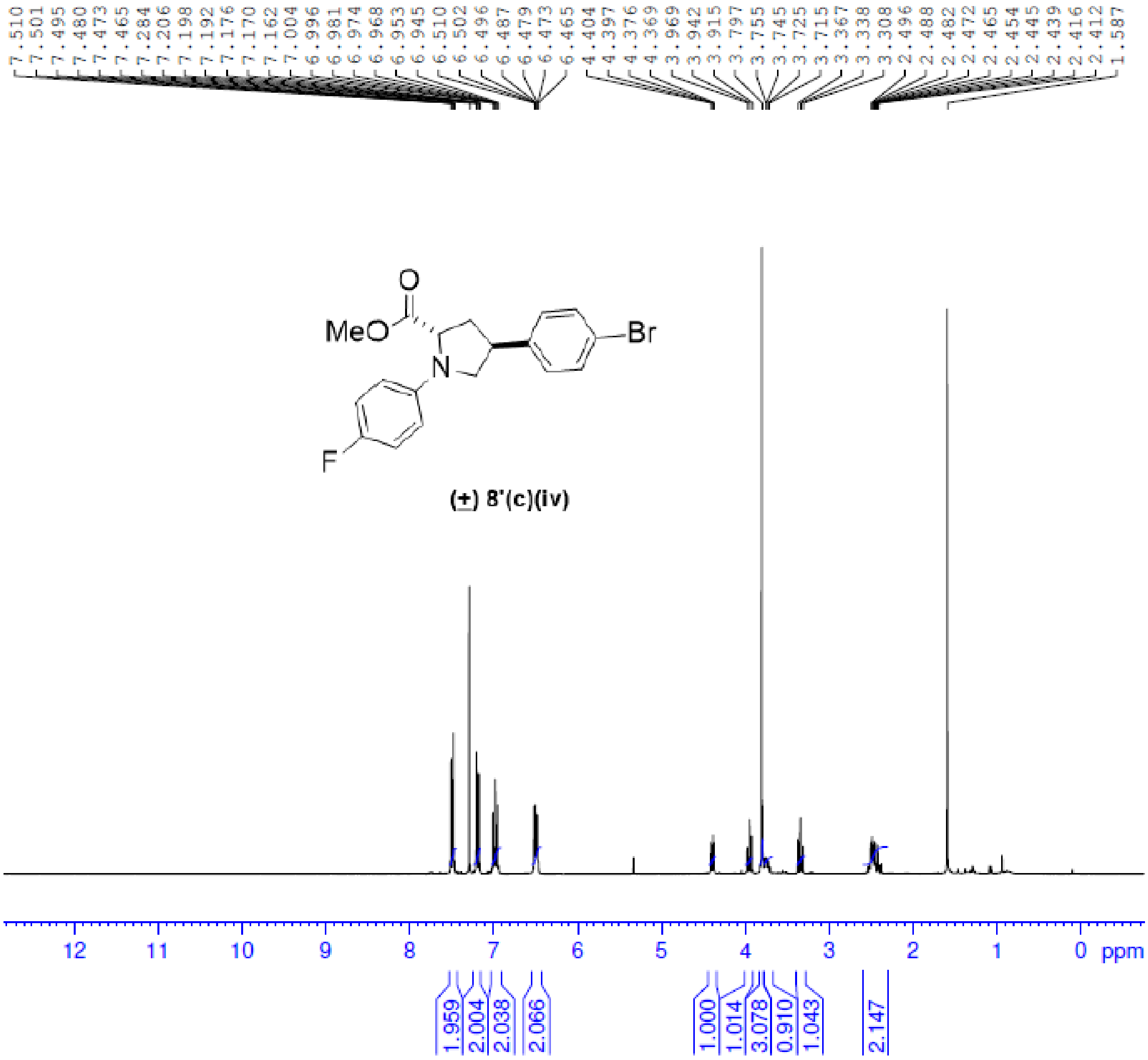

Figure S63. ^1H NMR spectrum of *(±) trans* 8'(c)(iv)

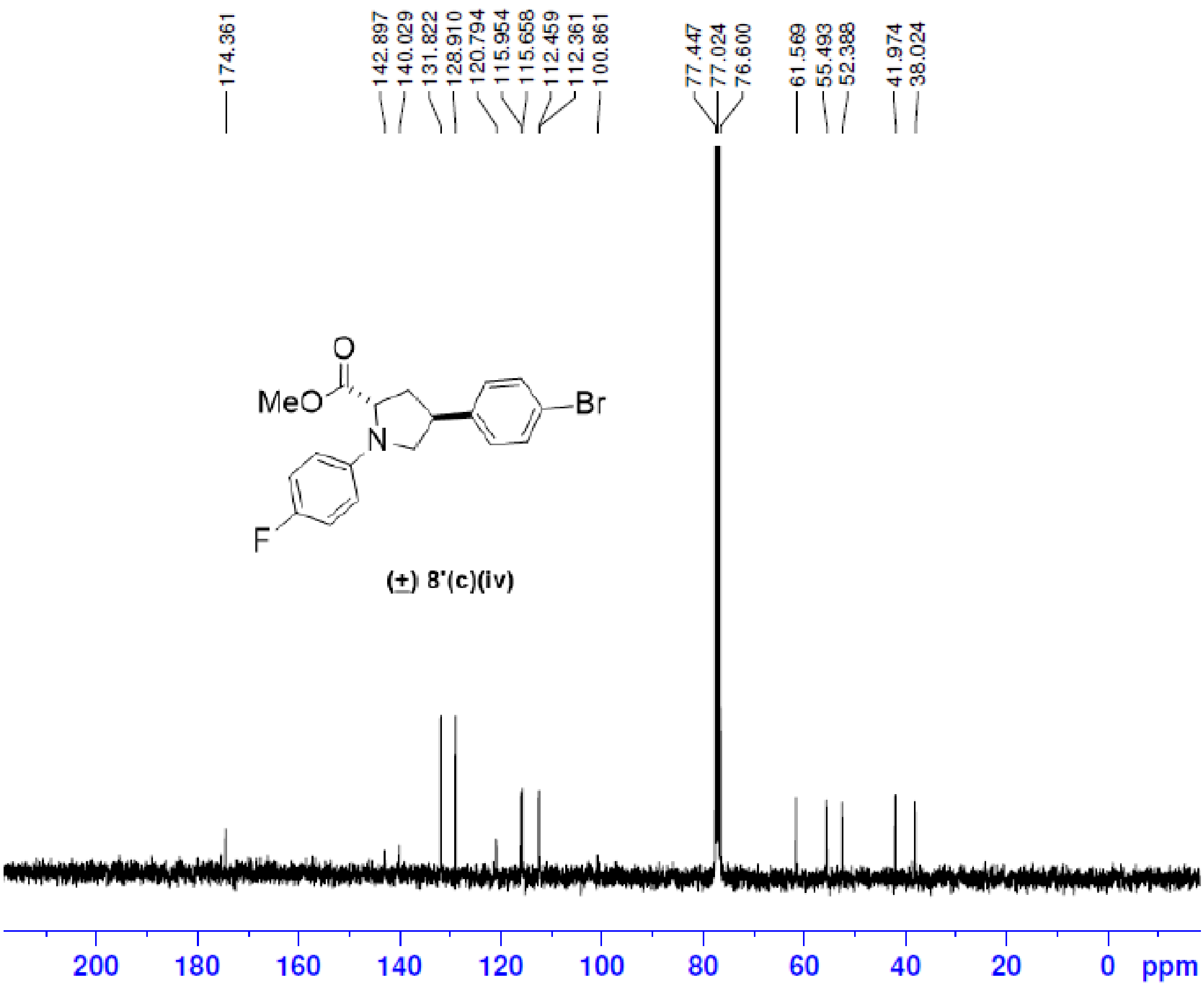

Figure S64. ^{13}C NMR spectrum of *(±) trans* 8'(c)(iv)

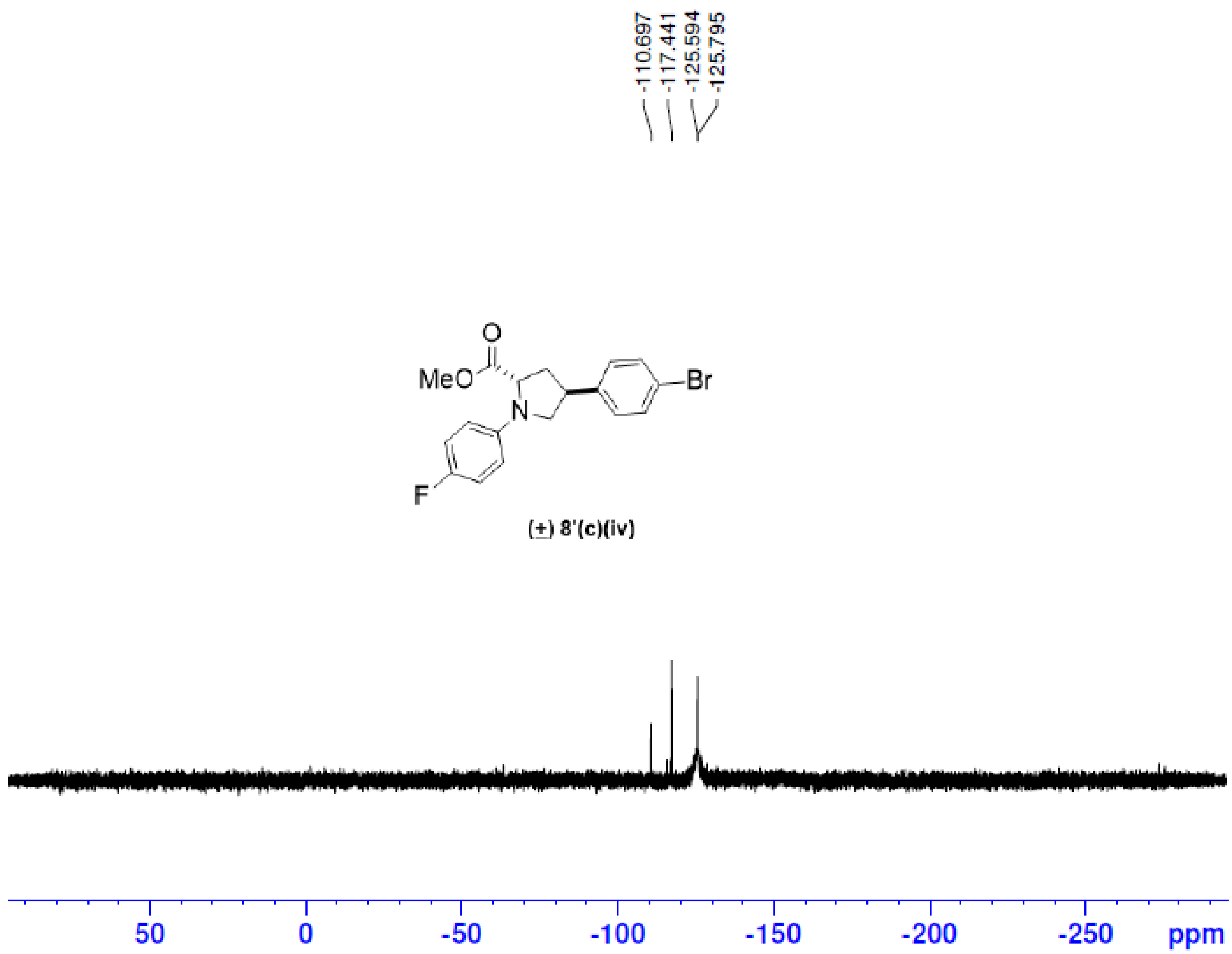

Figure S65. ^{19}F NMR spectrum of *(±) trans* 8'(c)(iv)

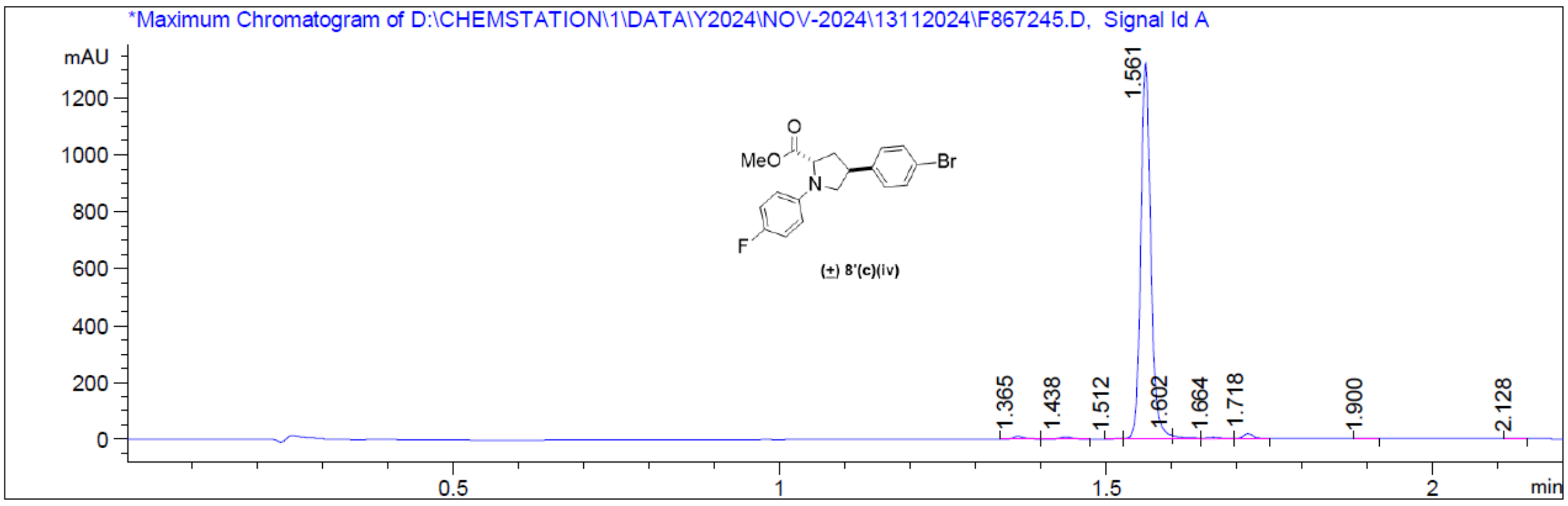

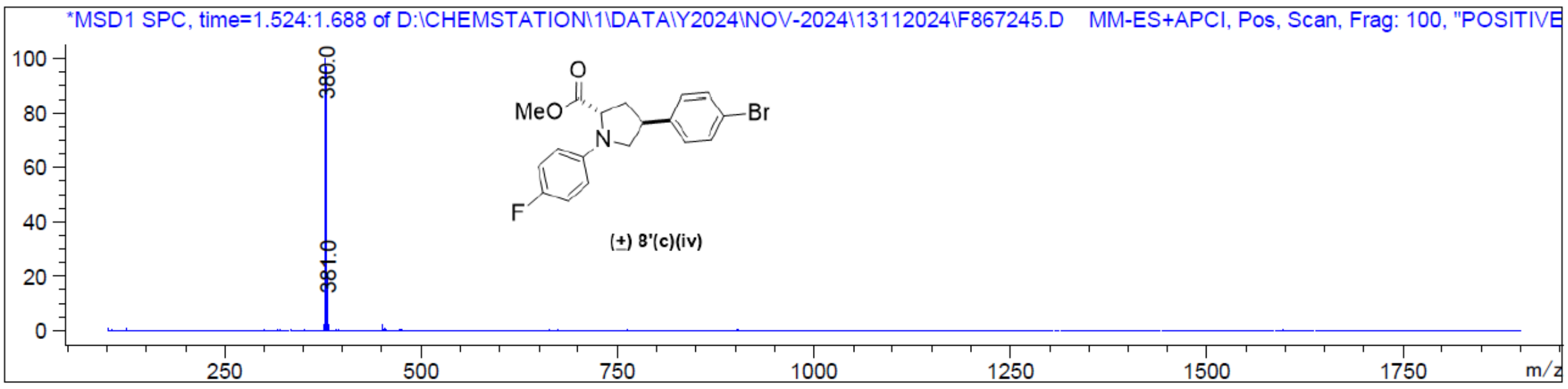

Figure S66. LC-MS data of *(±) trans* 8'(c)(iv)

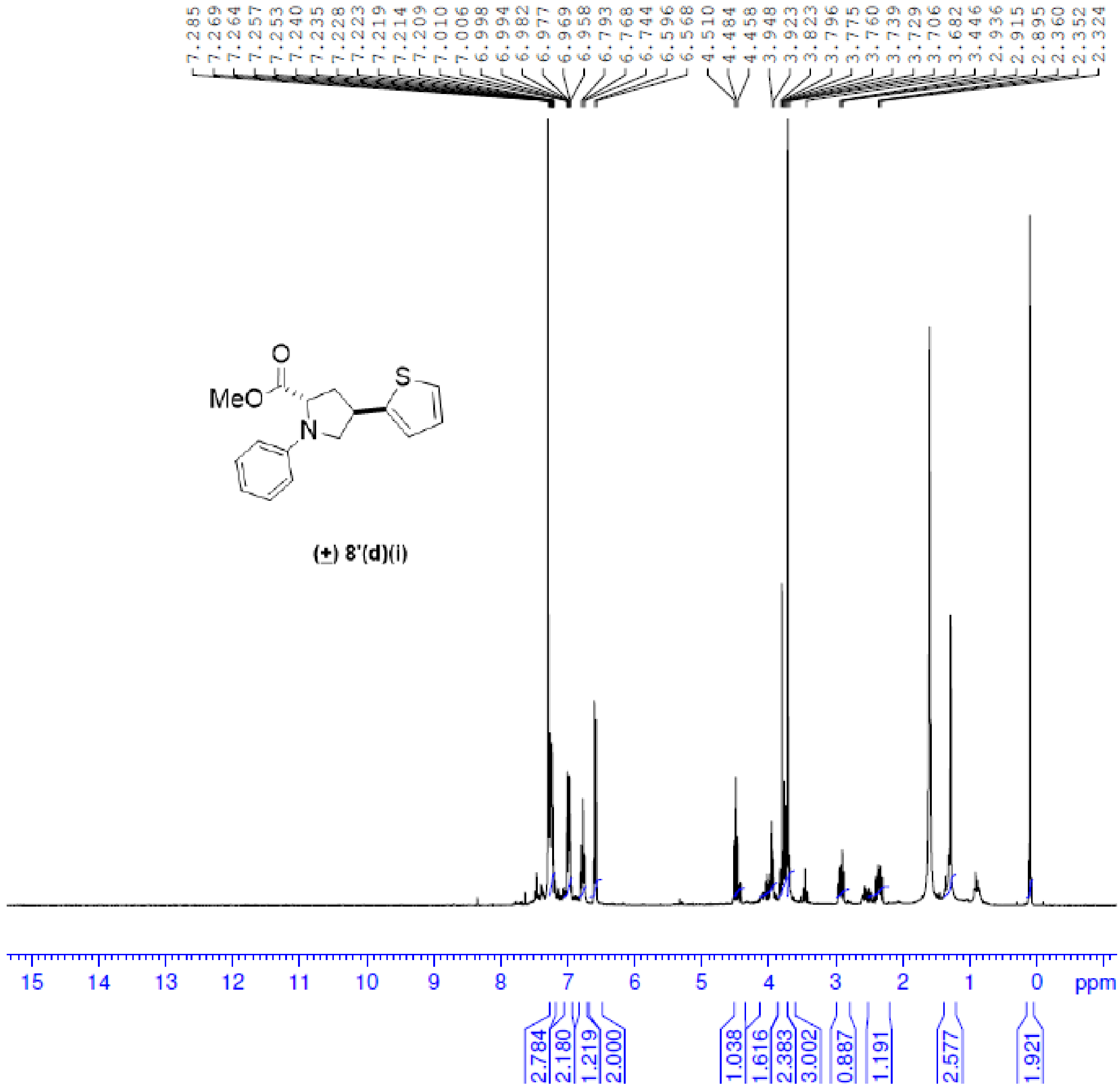

Figure S67. ^{1}H NMR spectrum of *(±) trans* 8'(d)(i)

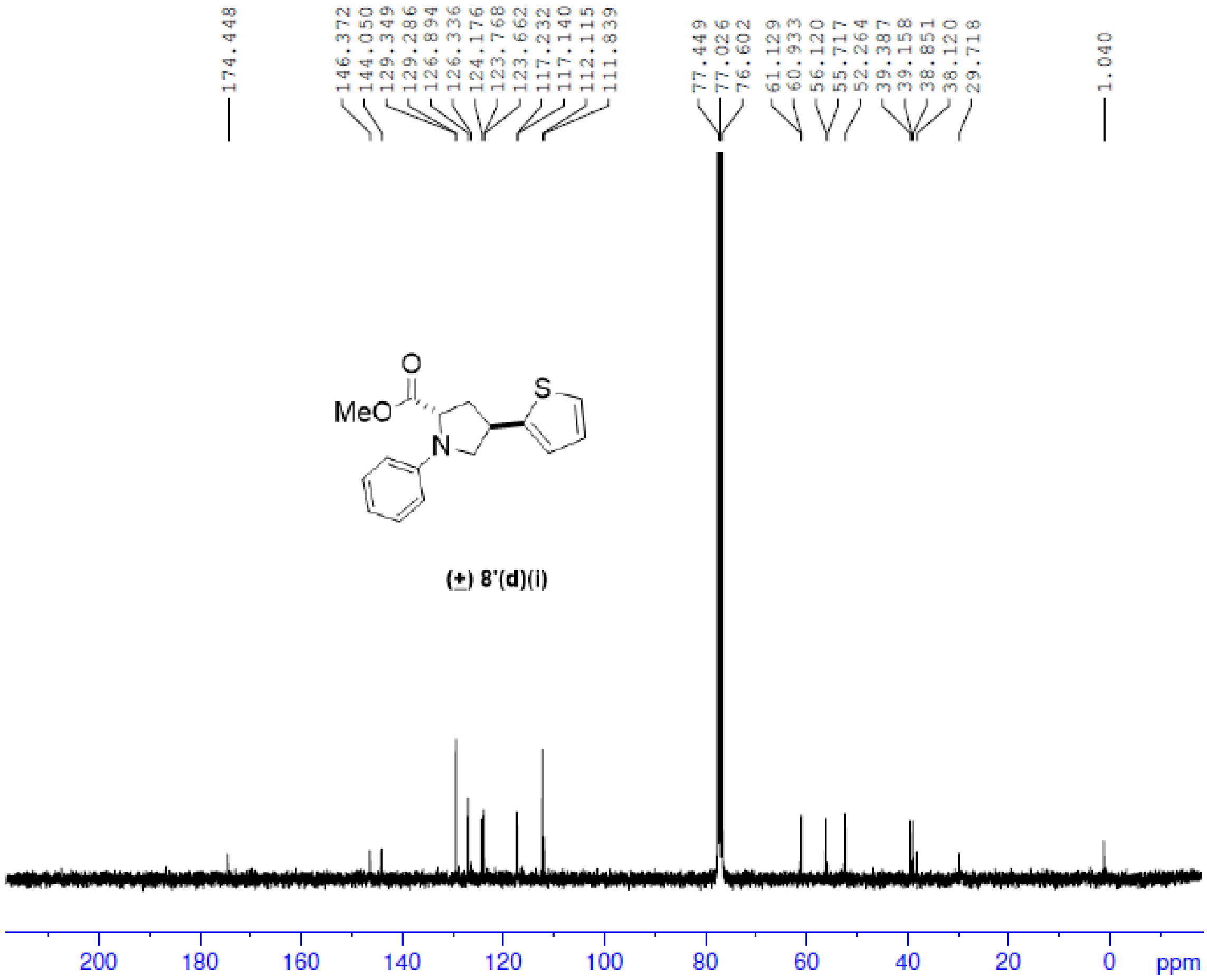

Figure S68. ^{13}C NMR spectrum of *(±) trans* 8'(d)(i)

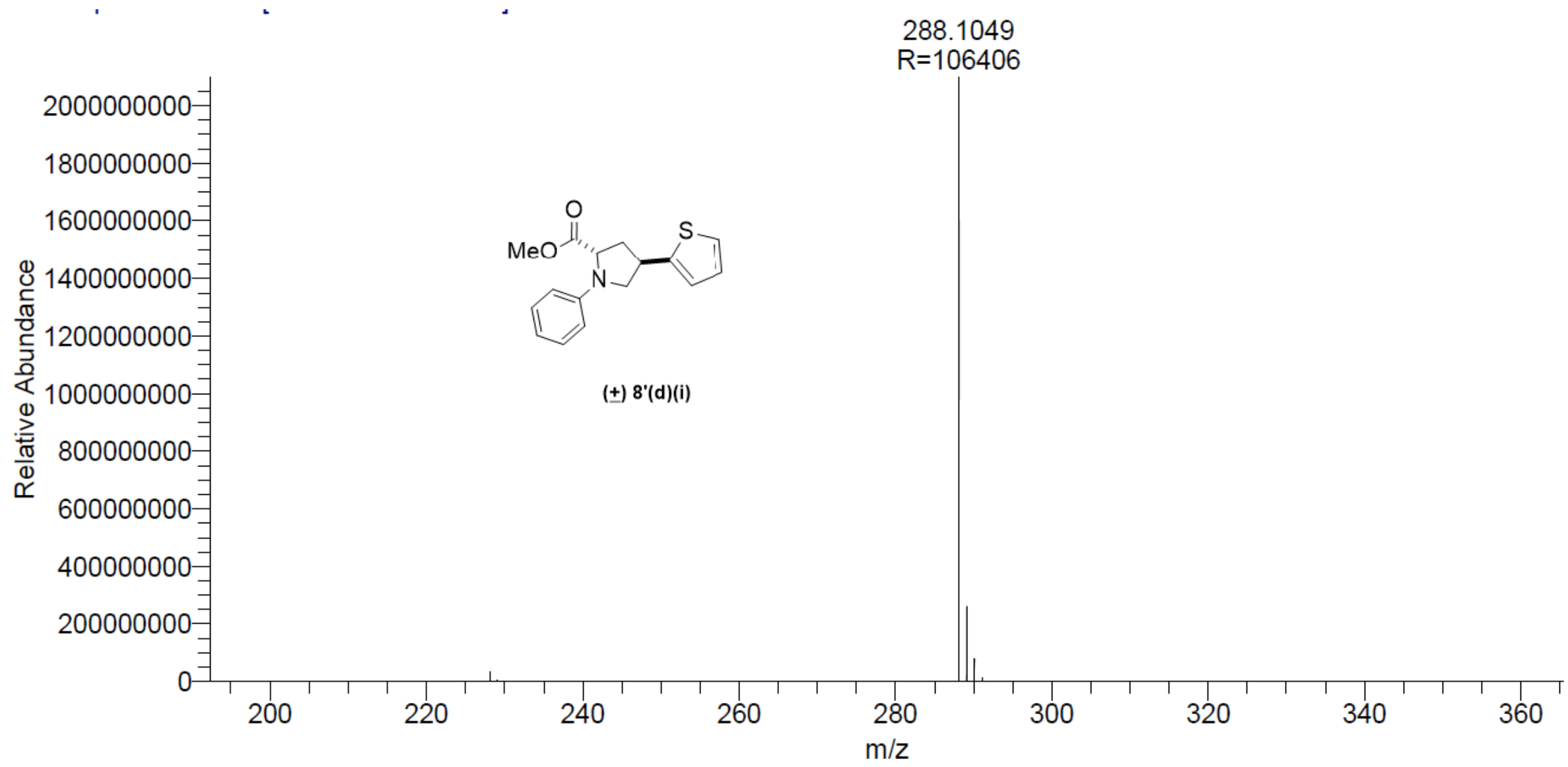

Figure S69. HRMS data of *(±) trans* 8'(d)(i)

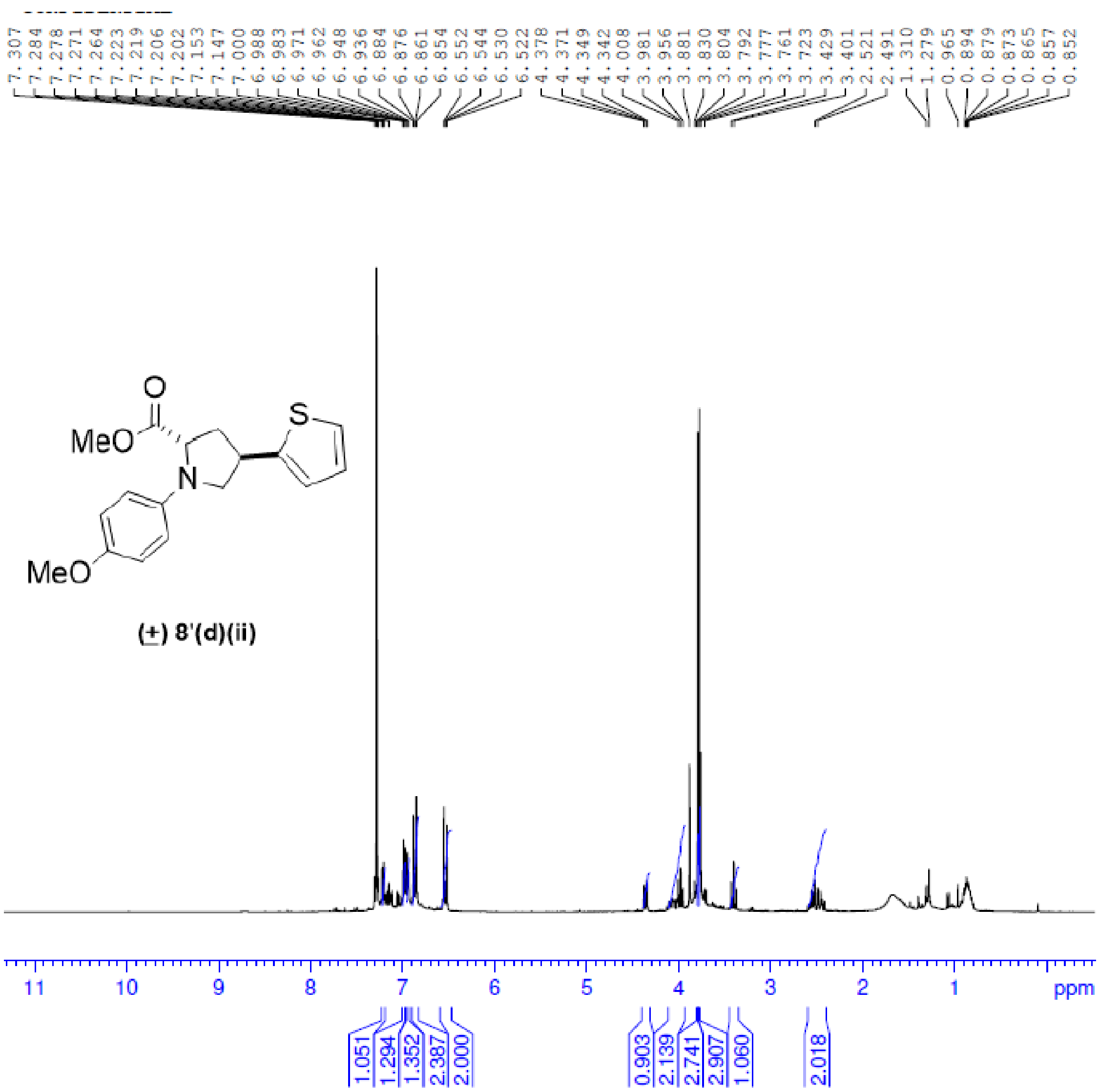

Figure S70. ^{1}H NMR spectrum of *(±) trans* 8'(d)(ii)

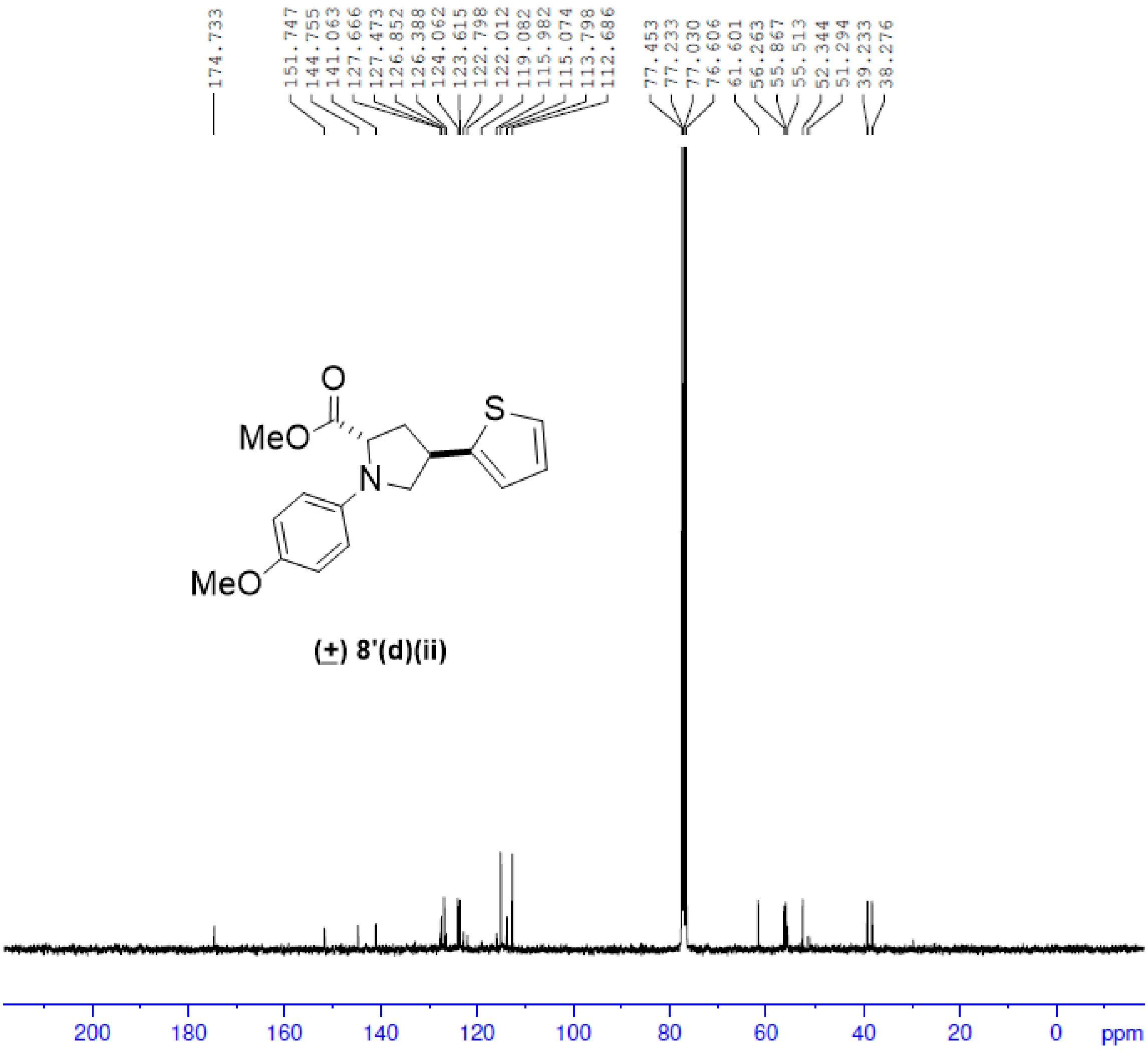

Figure S71. ^{13}C NMR spectrum of *(±) trans* 8'(d)(ii)

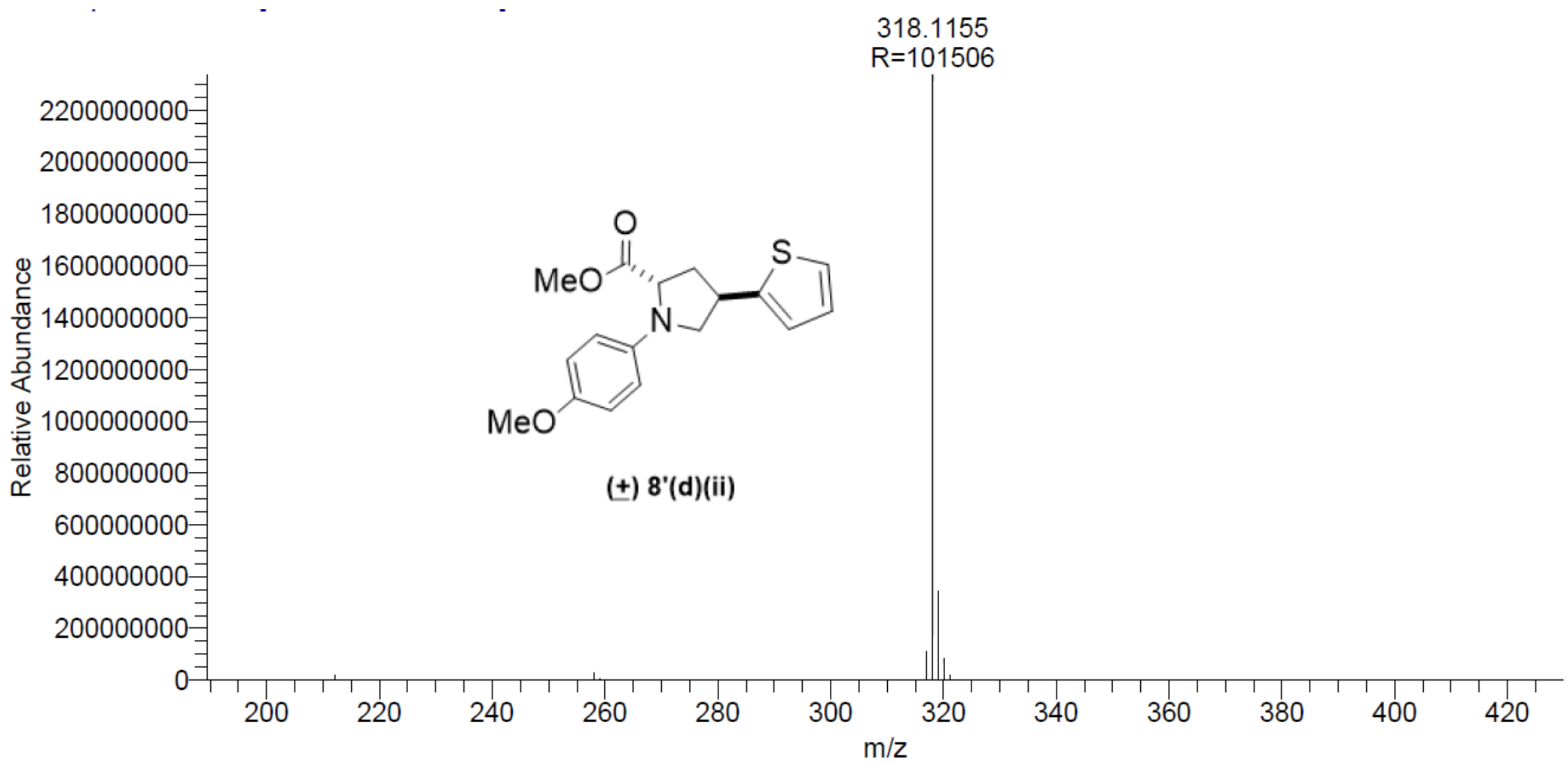

Figure S72. HRMS data of *(±) trans* 8'(d)(ii)

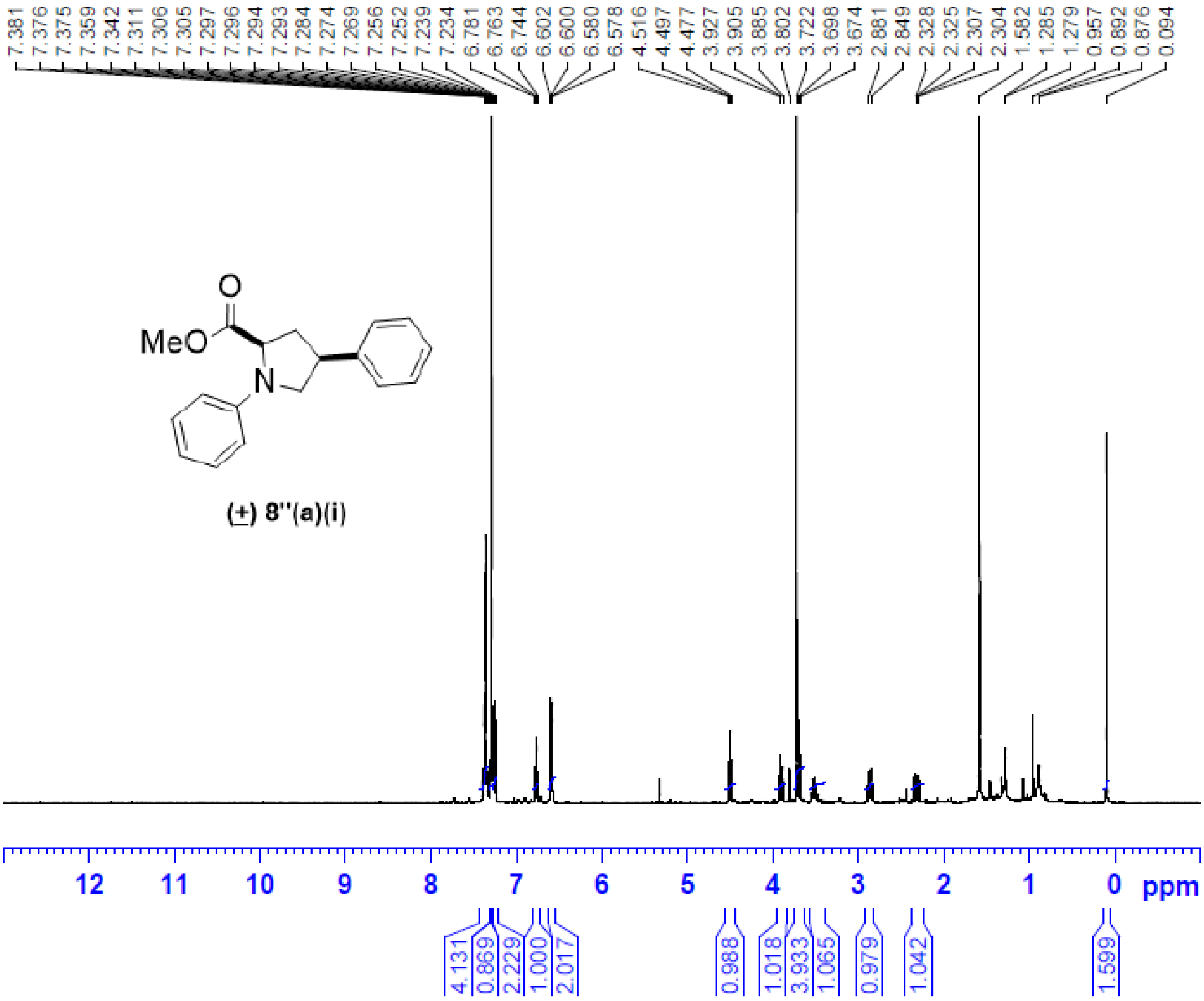

Figure S73. ^{1}H NMR spectrum of *(±) cis* 8’’(a)(i)

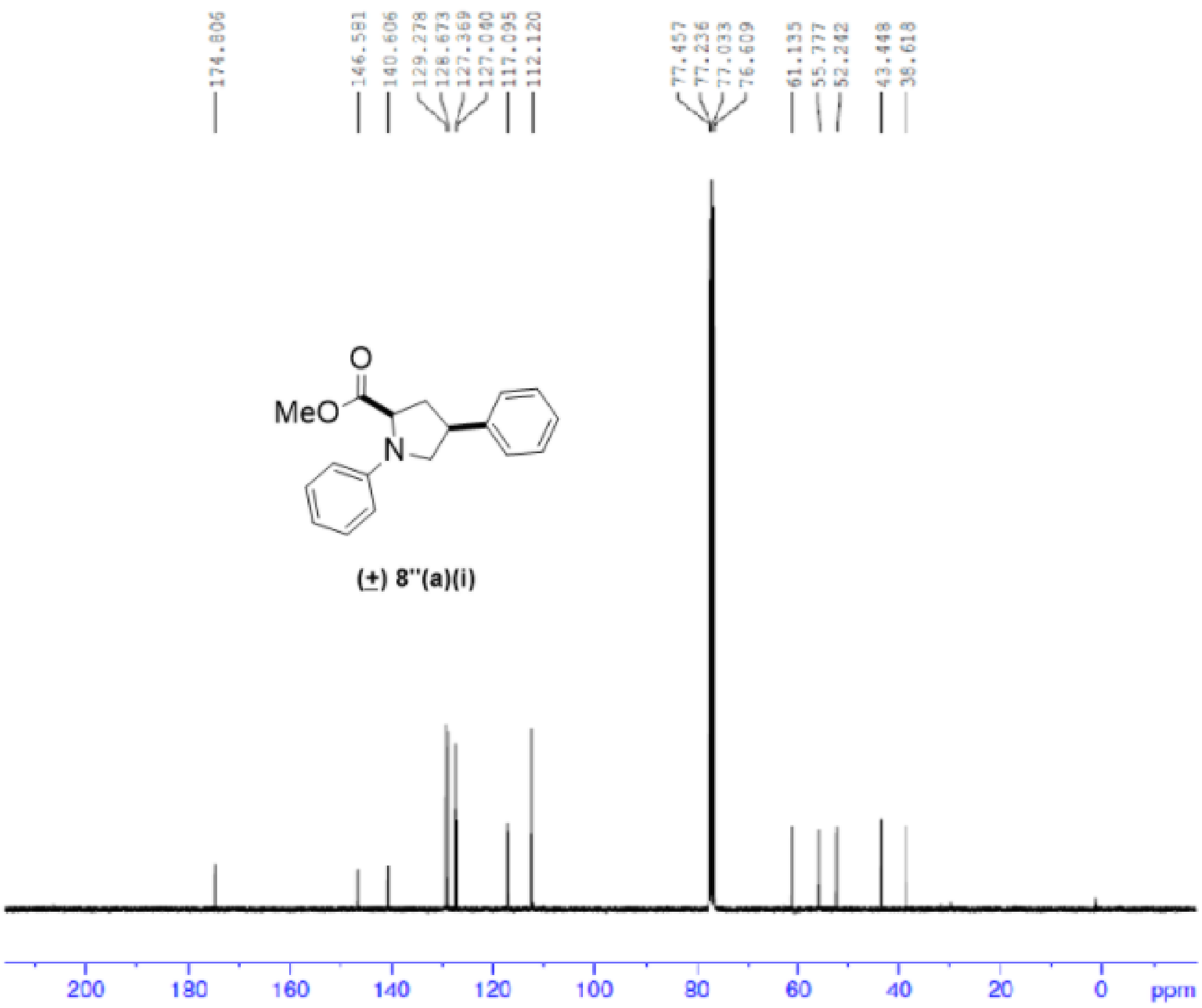

Figure S74. ^{13}C NMR spectrum of *(+) cis* 8''(a)(i)

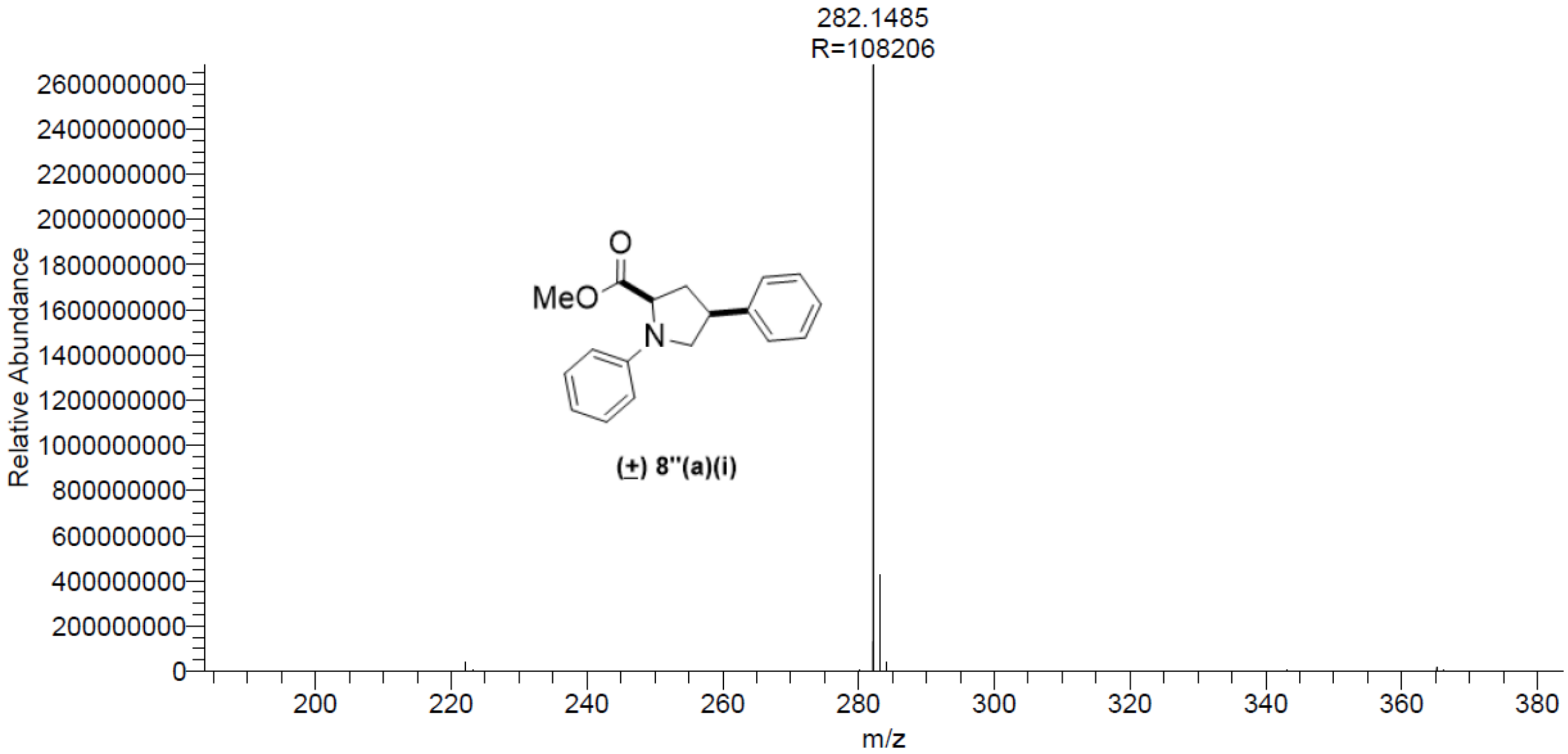

Figure S75. HRMS data of *(±) cis* 8''(a)(i)

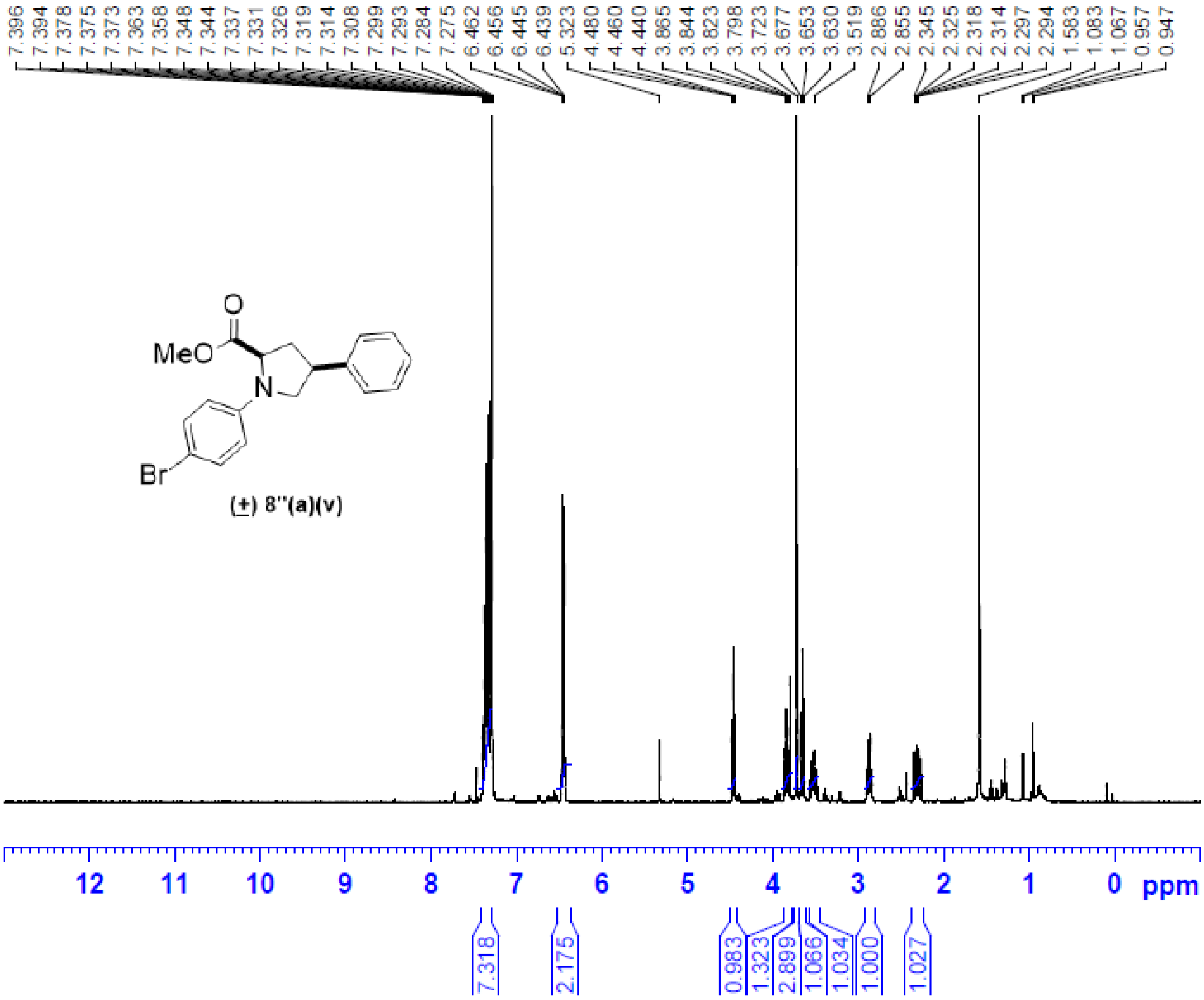

Figure S76. ^{1}H NMR spectrum of *(±) cis* 8''(a)(v)

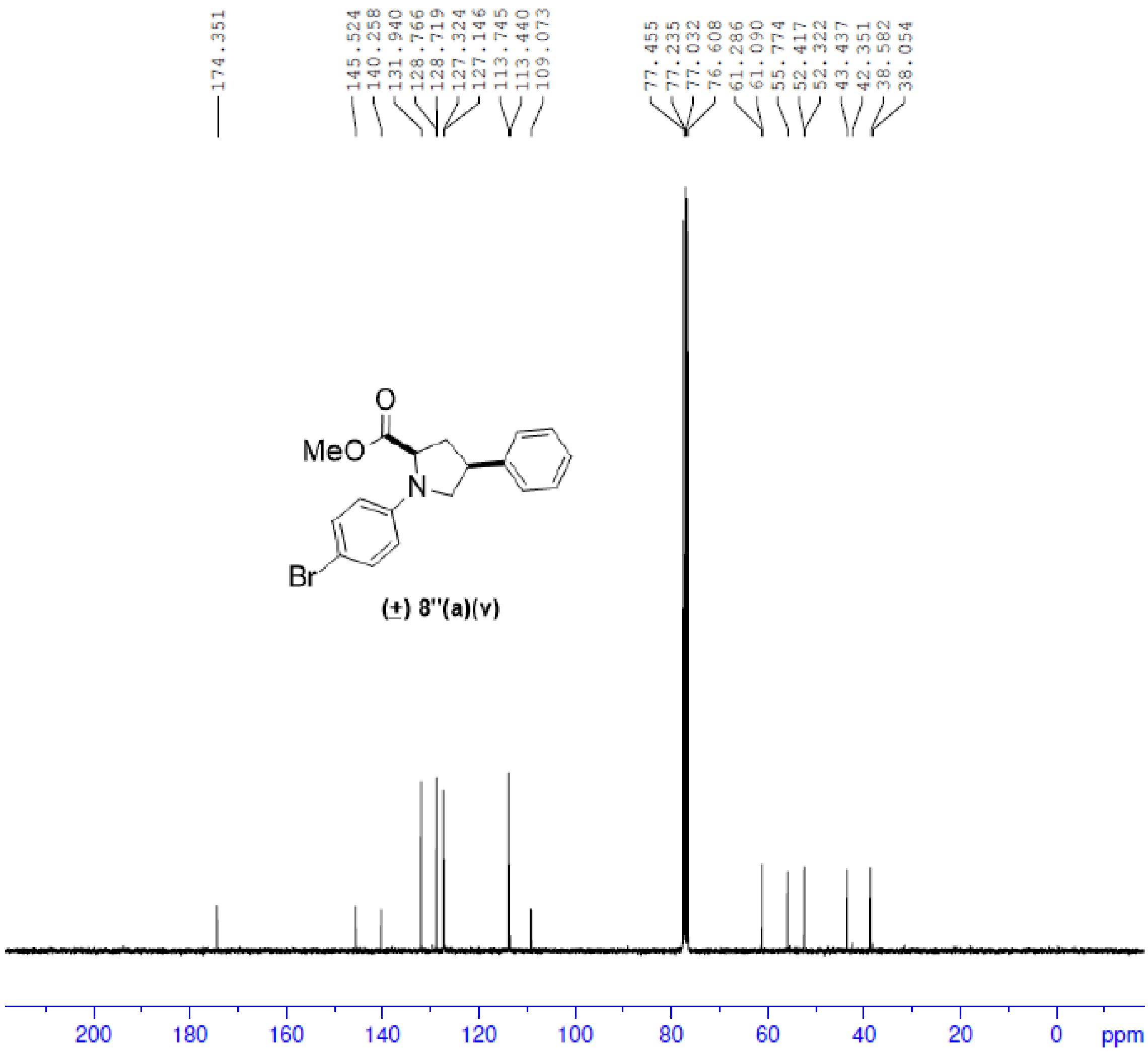

Figure S77. ^{13}C NMR spectrum of *(±) cis* 8''(a)(v)

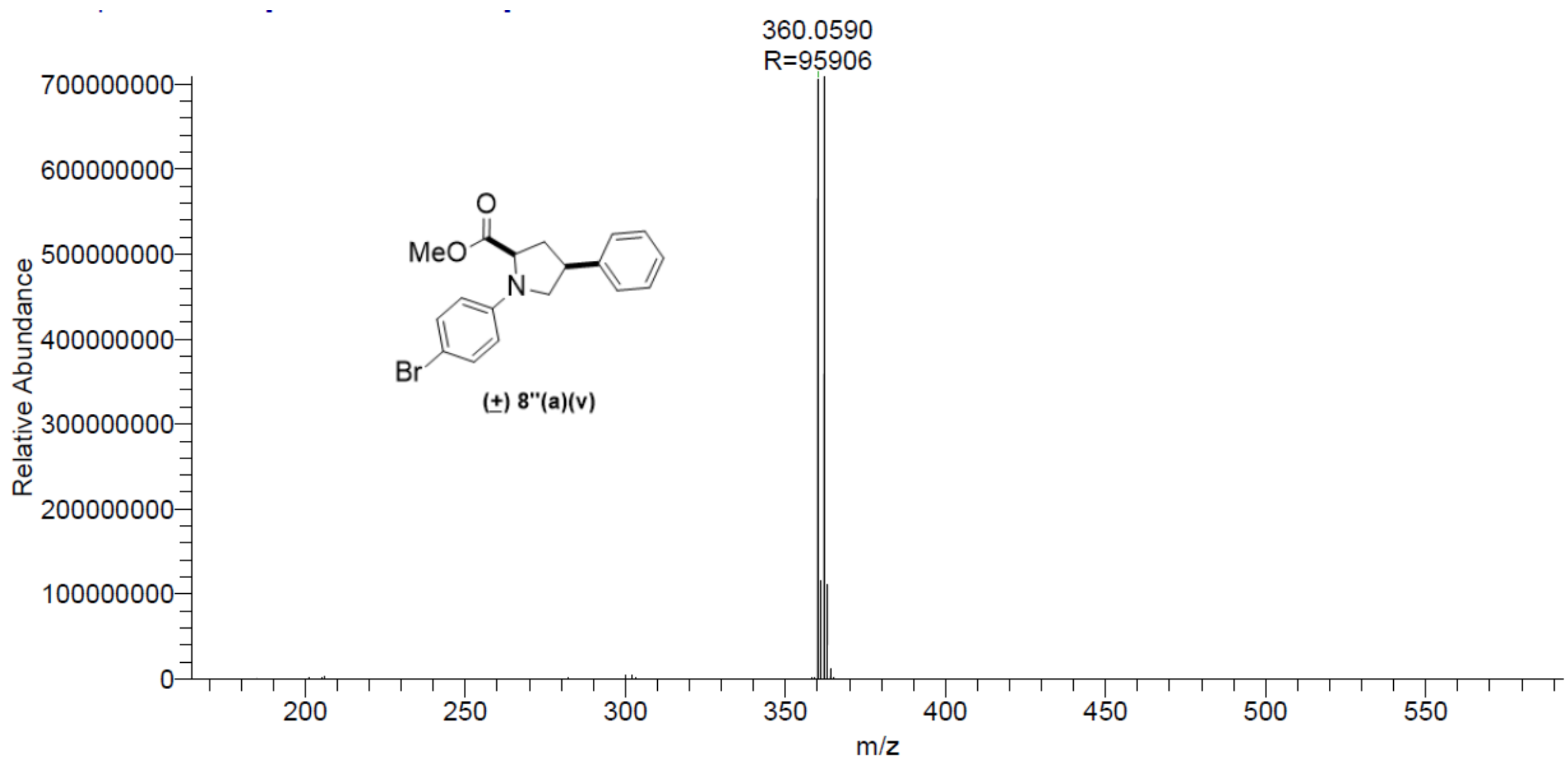

Figure S78. HRMS data of *(±) cis* 8''(a)(v)

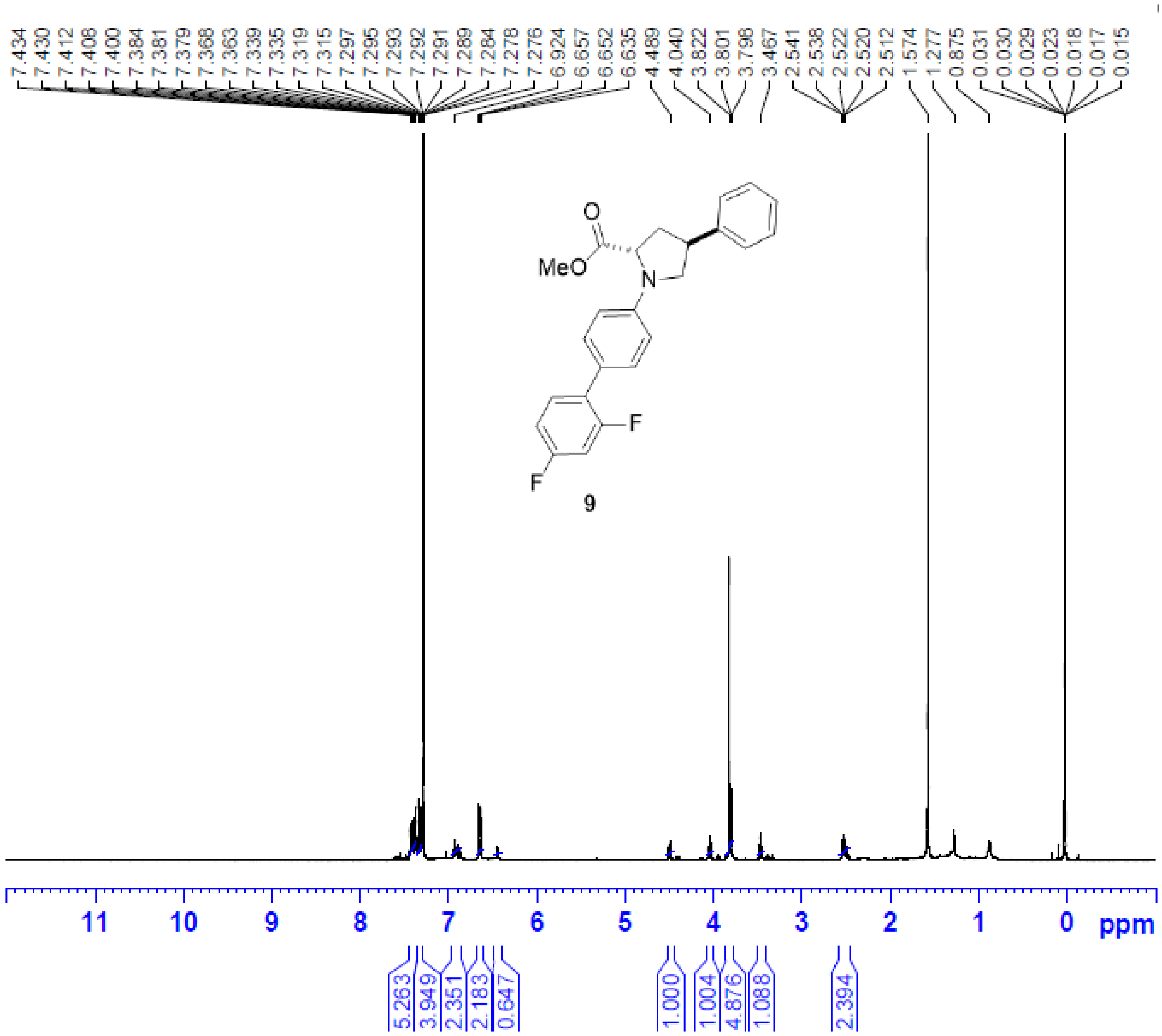

Figure S79. ^{1}H NMR spectrum of *(±) trans* 9

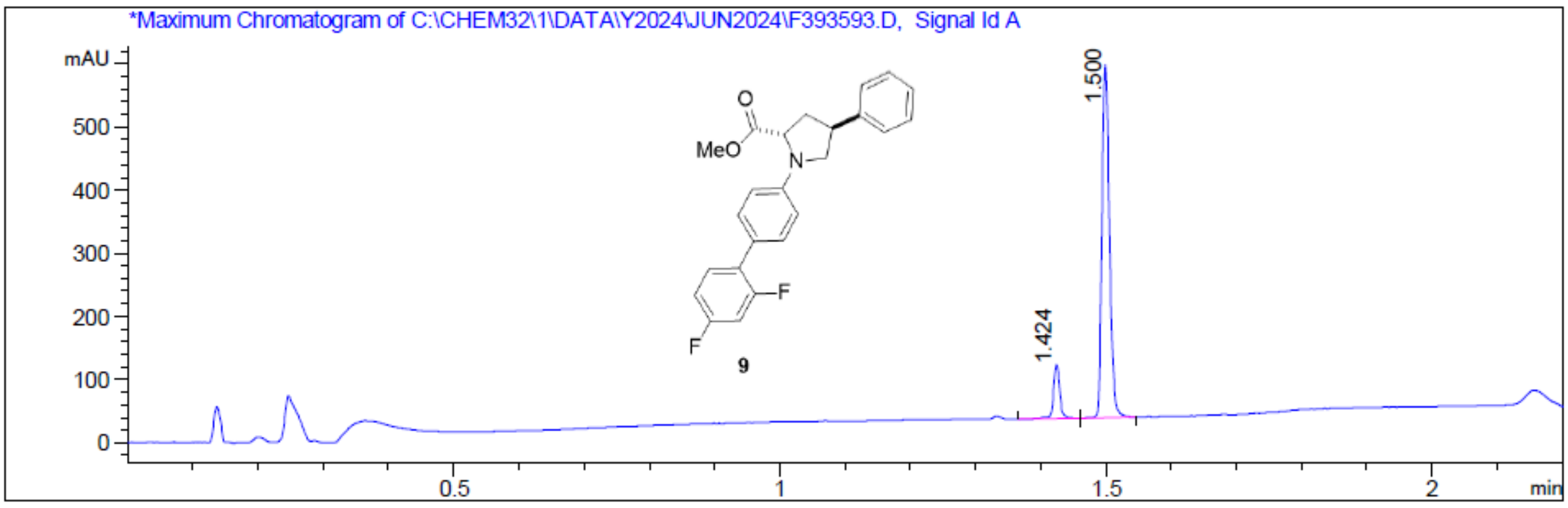

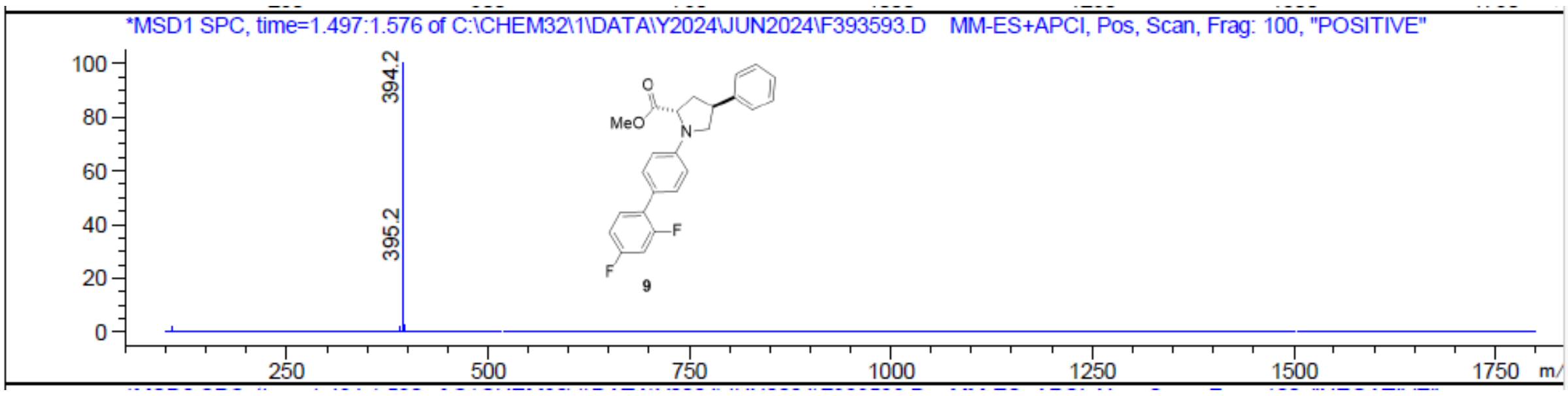

Figure S80. LC-MS data of *(±) trans* 9

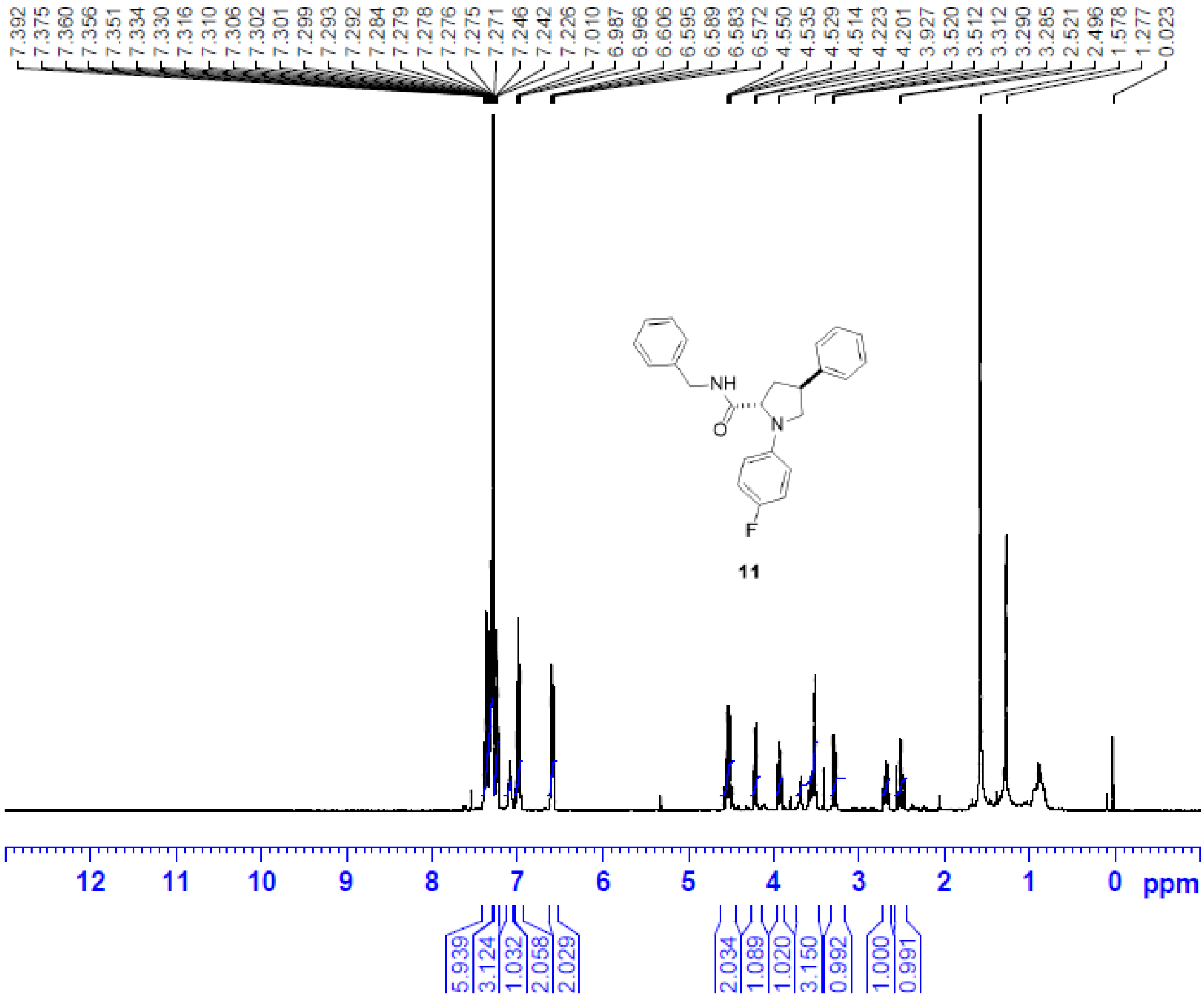

Figure S81. ^{1}H NMR spectrum of *(±) trans 11*

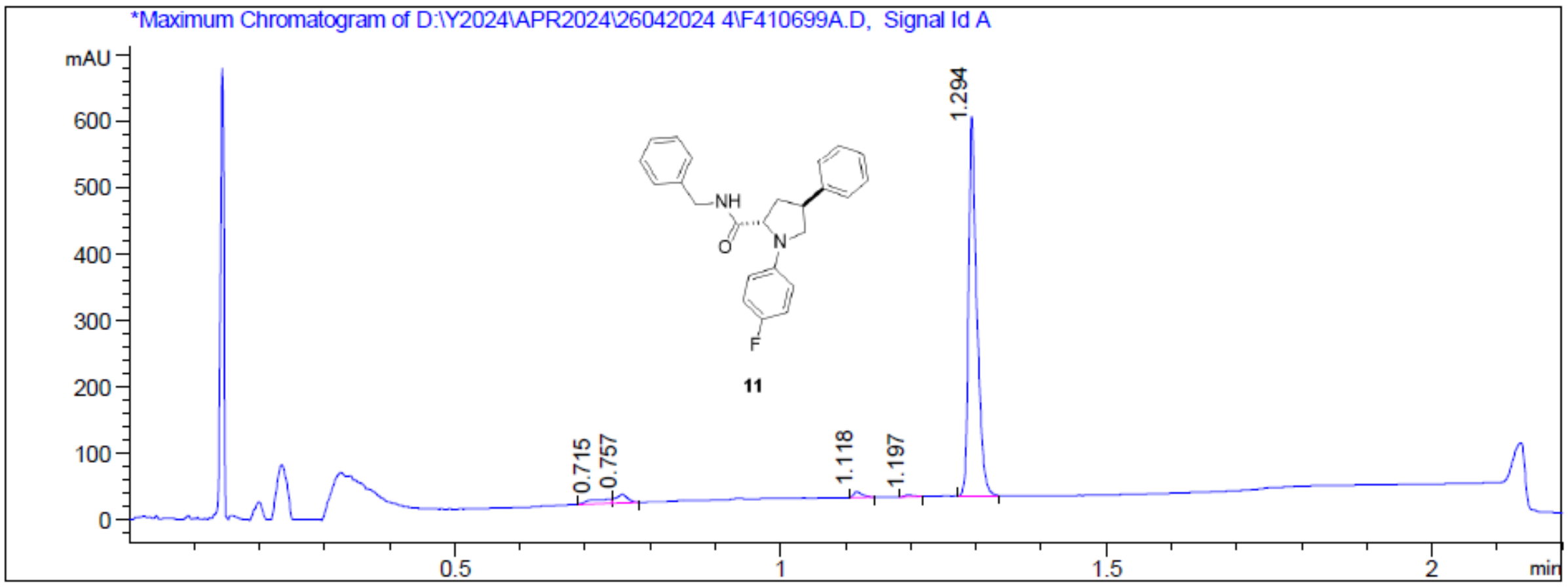

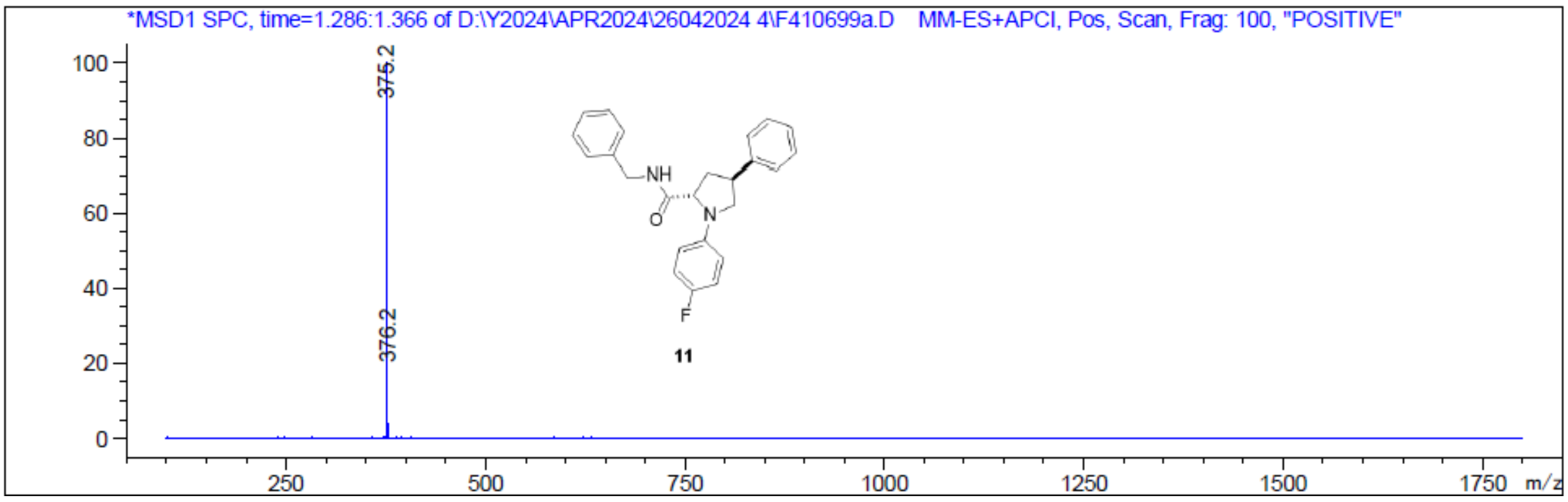

Figure S82. LC-MS data of *(±) trans 11*
